# Voluntary wheel running modulates murine gut microbiome during hyperammonemic stress

**DOI:** 10.1128/spectrum.03194-25

**Published:** 2026-07-15

**Authors:** Annette Bellar, Naseer Sangwan, Aaron Miller, Avinash Kumar, Thomas Jaramillo, Saurabh Mishra, Pugazhendhi Kannan, Amy Attaway, Lopa Mishra, Nicole Welch, Srinivasan Dasarathy

**Affiliations:** 1 Department of Inflammation and Immunity, Lerner Research Institute, Cleveland Clinic 22516, Cleveland, Ohio, USA; 2 Center for Microbiome Research, Lerner Research Institute, Cleveland Clinic 22516, Cleveland, Ohio, USA; 3 Cardiovascular and Metabolic Science, Lerner Research Institute, Cleveland Clinic 22516, Cleveland, Ohio, USA; 4 Department of Gastroenterology and Human Nutrition, All India Institute of Medical Sciences 28730, New Delhi, India; 5 Lerner Research Core Services, Lerner Research Institute, Cleveland Clinic 22516, Cleveland, Ohio, USA; 6 Department of Pulmonary Medicine, Cleveland Clinic 2569, Cleveland, Ohio, USA; 7 Feinstein Institutes of Medical Research, New York, USA; 8 Department of Gastroenterology and Hepatology, Cleveland Clinic 2569, Cleveland, Ohio, USA; University of Nebraska-Lincoln, Lincoln, Nebraska, USA

**Keywords:** gut microbiome, voluntary wheel running, hyperammonemia, cirrhosis, exercise, dysbiosis

## Abstract

Exercise modulates multiple physiological systems, including skeletal muscle and the gut microbiome (GMB). Ammonia, a microbiome-derived cytotoxic metabolite, causes cellular hyperammonemic stress (HAS) in chronic diseases. We investigated the impact of voluntary wheel running (VWR) on GMB during HAS in a mouse model. Male C57BL/6J mice were randomized to treatment with either ammonium acetate (AmAc) (2.5 mmol/kg/day) or vehicle for 6 weeks. Stool 16S rRNA sequencing was performed at baseline, pre-intervention, and post-intervention. GMB diversity, taxa-level abundance, and correlation analyses were performed. Overall GMB composition remained stable between baseline and pre-intervention across groups (*r* > 0.57; *P* < 0.001). Following interventions, VWR or usual activity (UA), alpha-diversity was highest in AmAc-treated, specifically AmAc-VWR, mice. *Eubacterium xylanophilum* was reduced in AmAc-UA vs other groups (*P* < 0.05). *Akkermansia* abundance declined over time in UA mice, but in AmAc-VWR mice, this depletion was reversed (*P* = 0.002). *Clostridium sensu stricto 1* and *Eubacterium ventriosum* were increased in AmAc-VWR mice (*P* < 0.05). Correlation analysis revealed high stability in PBS-UA (*r* = 0.667; *P* < 0.001), moderate restructuring in AmAc-VWR (*r* = 0.566; *P* < 0.001), and PBS-VWR (*r* = 0.385; *P* = 0.0099). HAS-induced GMB instability, with loss of beneficial taxa, including short-chain fatty acid-producing bacteria, was partially ameliorated by VWR. Exercise-mediated GMB modulation may be a strategy to mitigate HAS-induced complications in chronic diseases.

**IMPORTANCE:**

Voluntary exercise is recommended in chronic diseases to improve outcomes, but biological responses in disease are not well characterized. Perturbations in the metabolism of ammonia, a microbiome-generated toxin, occur in chronic diseases that can be compounded by muscle-generated ammonia during exercise. Exercise-induced molecular responses are adversely affected by hyperammonemic stress of chronic diseases, including liver cirrhosis. We investigated gut microbiome changes during voluntary wheel running, which replicates human endurance exercise in a preclinical mouse model of hyperammonemia. Adverse impacts of Hyperammonemic stress included a reduction in short-chain fatty acid producers that were reversed by voluntary wheel running. Our data lay the foundation for future studies on how endurance-type exercise promotes a favorable gut microbial composition and strategies to use exercise as a regulator of hyperammonemic stress via targeting the gut microbiome.

## INTRODUCTION

In chronic diseases, including cirrhosis, sarcopenia and fatigue are related to impaired ammonia disposal with consequent hyperammonemic stress (HAS) in cells (1). In addition to these well-recognized sequelae, patients with cirrhosis and other chronic diseases have an altered gut microbiome (GMB) (2, 3). Gut dysbiosis contributes to the progression of liver disease and may exacerbate extrahepatic complications (4–6). Specifically, altered GMB contributes to lower skeletal muscle mass (7), sarcopenia (8), endothelial dysfunction (9), and hepatic encephalopathy (10). Ammoniagenesis by the GMB is a significant source of hyperammonemia in cirrhosis (11), and HAS causes perturbations in multiple organs in disease (12), supporting a link between microbiome composition, nitrogen metabolism, and systemic complications. Current evidence shows that HAS causes sarcopenia or skeletal muscle loss and contractile dysfunction, which contribute to physical frailty, resulting in greater morbidity and mortality in chronic diseases (1). There is also compelling evidence that dysbiosis in chronic diseases, especially cirrhosis, contributes to increased GMB-derived ammoniagenesis (3, 10, 11). In chronic diseases, exercise increases muscle ammoniagenesis, which can limit beneficial responses and potentially contribute to tissue injury (13–15).

Exercise modulates the GMB and promotes taxa associated with gut and systemic health (13, 16). Unlike forced exercise protocols, voluntary wheel running (VWR) in mice replicates the responses to human endurance exercise (14, 17, 18). A systematic review evaluating human and preclinical data concluded that in humans, exercise lowered the Bacilliota/Bacteroidetes ratio, with consistent increases in *Bacteroides* and *Roseburia* genera. Endurance exercise increases alpha-diversity with expansion of beneficial taxa, including *Faecalibacterium prausnitzii, Oscillospira, Lachnospira, Coprococcus*, *Ruminococcaceae* (19), and members of the Bacilliota phylum in preclinical models and humans (20–23), while sedentary high-fat-fed mice had decreased *Bacteroides* (24). *Faecalibacterium prausnitzii* serves as a key anti-inflammatory butyrate producer and is reduced in inflammatory bowel disease. *Oscillospira* is linked to leanness, favorable plasma glucose and fatty acid profiles, and bile acid regulation (25, 26). *Lachnospira* supports fiber degradation, short-chain fatty acid (SCFA) production, immune tolerance, and metabolic health. *Coprococcus* promotes gut barrier integrity and is linked to mental health via gut-brain axis signaling (27, 28). Thus, multiple metabolite-driven pathways contribute to microbiome-mediated beneficial responses to voluntary endurance exercise (29).

The mode of exercise is also an important determinant of physiological and microbial responses (30). Unlike VWR, forced exercise protocols (swim-to-exhaustion, forced treadmill running) induce stress in rodents that do not consistently recapitulate human endurance adaptations (17, 31, 32). Despite robust data in healthy rodents, there are few data on the GMB responses to VWR in disease models. Recent data show that VWR partially reverses the consequences of HAS in mice (14, 15); however, it is not known if the GMB changes contribute to these beneficial effects. Bacterial taxa can either produce (33, 34) or metabolize ammonia (35), and exercise-induced modulation of the microbiome can influence systemic ammonia handling. In cattle, the types of substrates utilized and free ammonia have been reported to determine GMB (36), but whether such responses occur in rodent models is not known. Therefore, we evaluated the interaction between VWR and HAS in a validated mouse model with constant infusion of ammonium acetate (AmAc) (14).

We observed that in usual activity (UA) and AmAc-treated mice, beneficial taxa that are SCFA producers (*Eubacterium* spp., *Akkermansia* spp.) are less abundant, while VWR partially reversed these changes. Although taxonomic level changes in ammoniagenic bacteria did not change, the abundance of *Clostridium sensu stricto* was higher, suggesting an increase during HAS. These data lay the foundation for evaluating the mechanistic basis of GMB changes in response to exercise, as well as the impact of ammonia-lowering measures (37) to enhance beneficial responses to exercise in chronic diseases with HAS in humans.

## MATERIALS AND METHODS

### Animals and experimental design

Male C57BL/6J mice (Jackson Laboratory, Bar Harbor, ME, USA) aged 8–9 weeks were used for all experiments, with details of the protocol reported earlier (14). Animals were individually housed in specific pathogen-free conditions at the Cleveland Clinic Biological Resources Unit, maintained on a 12:12-h light–dark cycle, and provided *ad libitum* access to standard chow (Teklad Global 18% Protein Rodent Diet, Envigo, 2918) and water. After a 1 week acclimatization period, mice were randomized to receive either AmAc (2.5 mmol/kg/day) or phosphate-buffered saline (PBS, vehicle control) via subcutaneous osmotic mini-pumps (Alzet model 1004, DURECT Corp., Cupertino, CA), which were delivered continuously for 6 weeks. Pumps were implanted under isoflurane anesthesia with buprenorphine analgesia for postoperative pain management. Our mouse model of HAS is characterized by elevated muscle ammonia levels, which replicate those reported in skeletal muscle in patients with cirrhosis (38–40). These mice had normal serum transaminases, plasma glucose, insulin, and urea nitrogen (14), and no visible changes in the liver, allowing isolation of metabolic stress effects independent of primary hepatic injury. Even though cognitive or behavioral studies were not performed, grooming or voluntary activity was not limited (14).

### Voluntary wheel running

Two weeks after pump implantation, mice in each treatment group (AmAc or PBS) were further randomized to VWR or UA. VWR was implemented using cages equipped with low-resistance running wheels (Med Associates, ENV-044, St. Albans, VT, USA). An automated counter continuously recorded wheel revolutions. Mice in UA groups were housed in identical cages with the wheels locked. Running distance and duration were quantified over the 4 week intervention period, and mice were monitored daily for health status. This model allows animals to run during their natural nocturnal cycle without external stressors (14, 17).

### Stool collection and DNA extraction

Stool pellets were collected at three time points: baseline (prior to pump implantation), pre-intervention (2 weeks post-implantation, before wheel running), and post-intervention (after 4 weeks of VWR or UA). Fresh stool was collected directly into sterile tubes. Zymo Research DNA/RNA Shield was added immediately, and the samples were stored at 4°C until processing. DNA extraction was performed using the QIAamp Fast DNA Stool Mini Kit (Qiagen, Hilden, Germany, 51604) following the manufacturer’s instructions with an additional bead-beating step using sterile zirconia/silica beads (0.1 mm; BioSpec, Bartlesville, OK) in a FastPrep-24 instrument (MP Biomedicals). DNA quantity and quality were assessed by NanoDrop spectrophotometry (Thermo Fisher Scientific) and Qubit fluorometry (dsDNA HS Assay Kit, Thermo Fisher, Q32854).

### 16S rRNA gene sequencing

The V4 region of the bacterial 16S rRNA gene was amplified using primers 515F and 806R (41). PCR was carried out with Phusion High-Fidelity DNA Polymerase (New England Biolabs, M0530). Thermocycler conditions were as follows: 98°C for 30 s; 25 cycles of 98°C for 10 s, 55°C for 20 s, and 72°C for 20 s; and a final extension at 72°C for 5 min. Amplicons were purified with AMPure XP beads (Beckman Coulter, A63881) and indexed with the Nextera XT Index Kit (Illumina, FC-131-1002). Libraries were quantified, pooled in equimolar concentrations, and sequenced using 2 × 250 bp paired-end chemistry on the Illumina MiSeq platform at the Cleveland Clinic Microbiome Core with a 20% PhiX spike-in. Extraction blanks and no-template PCRs were processed alongside experimental samples as negative controls. Raw reads were evaluated with FastQC (42).

### Bioinformatics analysis

Sequencing data were processed using QIIME2 (42) (version 2023.2). Reads were denoised, merged, and chimera-checked using DADA2 (43) to generate amplicon sequence variants (ASVs). Taxonomic assignment was performed using a naïve Bayes classifier trained on the SILVA 138 reference database (44). Alpha-diversity was determined by the Shannon index. Beta-diversity was assessed using canonical correspondence analysis (CCA) based on unweighted and weighted UniFrac distances and Bray-Curtis dissimilarity metrics (45, 46), with statistical significance tested using permutational multivariate analysis of variance (PERMANOVA) with 999 permutations.

### Differential abundance and network analysis

Differential taxonomic abundance was assessed using ANCOM-II (Analysis of Composition of Microbiomes) with Benjamini-Hochberg correction for false discovery rate (FDR < 0.05). Relative abundances were visualized at the genus level. Microbial co-occurrence networks were generated using SparCC correlation coefficients and visualized in Cytoscape (version 3.9). Nodes represent bacterial taxa, while edges represent significant correlations (|*r*| > 0.3, *P* < 0.05) after multiple testing correction.

### Machine learning analysis

Supervised machine learning was conducted using the random forest algorithm implemented in the R package randomForest (47). The model was trained to classify samples by treatment and exercise status. Feature importance was estimated using the mean decrease in Gini index. Model performance was assessed with 10-fold cross-validation, and accuracy was compared against null distributions generated by label permutations to assess model robustness. Model stability and performance were evaluated across cross-validation folds. Important taxa identified by the model were further validated by differential abundance testing. To reduce overfitting, model performance was compared against null distributions generated by label permutation.

### Statistical analysis

Statistical analyses were performed in R (version 4.3.0) and GraphPad Prism (version 9.0). Alpha-diversity differences between groups were tested using White’s non-parametric *t*-test with bootstrapped *P*-values. Beta-diversity differences were tested by PERMANOVA and visualized as CCA plots. Differential abundance between taxa was tested using White’s non-parametric *t*-test. For the Bacilliota/Bacteroides ratio, White’s non-parametric test was used for *post hoc* analysis. For functional analysis, differences in SCFA-producing bacteria were tested using White’s *t*-test on ASIN2 permuted data. For ammonia-producing bacteria, Yeo-Johnson-transformed abundances were compared using the Kruskal-Wallis test and Dunn’s test with a Benjamini-Hochberg correction. Taxonomic differences were adjusted for multiple testing using Benjamini-Hochberg FDR correction. Longitudinal changes within groups were analyzed with paired Wilcoxon signed-rank tests. *P*-values <0.05 were considered statistically significant unless otherwise specified. For ASV-level correlation analysis and visualization, ASV-level Spearman correlation matrices were computed within each time point (baseline and post), and the difference in correlation structure was visualized via heatmaps. ASVs were relabeled sequentially (e.g., ASV1, ASV2, etc.) for clarity. Taxonomic identities for each ASV were preserved in a separate mapping file linking ASV number to full taxonomy. Heatmaps were generated using the ComplexHeatmap and pheatmap packages in R. Clustering was performed using hierarchical clustering with complete linkage on distance matrices derived from correlation coefficients. Only high-confidence ASVs with adequate abundance across samples were retained. All pairwise correlation coefficients and statistical comparisons are provided in Table S1 and S2.

Microbial functional profiles were inferred from 16S rRNA gene sequencing data using both PICRUSt2 and Tax4Fun2. ASVs were generated from raw FASTQ files using the DADA2 pipeline in R, including quality filtering, error modeling, dereplication, and chimera removal. A non-chimeric ASV table and representative sequences were exported for downstream analyses. For PICRUSt2 analysis, ASV sequences and abundance tables were converted to BIOM format and processed using the picrust2_pipeline.py workflow with SEPP placement to predict KEGG Ortholog (KO), Enzyme Commission (EC), and MetaCyc pathway abundances. Functional profiles were imported into R and converted to relative abundance or log-transformed values (log_2_[x + 1]) for statistical analysis. Tax4Fun2 was performed using the Ref100NR database, in which ASV sequences were aligned to reference genomes and functional predictions were generated via nearest-neighbor mapping with 16S copy-number normalization. KO abundances were further aggregated into pathway-level profiles for comparison.

Statistical analyses were conducted across four experimental groups (AmAc-UA, AmAc-VWR, PBS-UA, PBS-VWR). Pairwise comparisons between groups were performed using two-sample *t*-tests on log-transformed abundance values to identify differential pathways and KOs. Paired analyses (pre vs post) were conducted using linear modeling with empirical Bayes moderation (limma), incorporating MouseID as a blocking factor to account for repeated measures. Multiple testing correction was applied to functional pathway analyses using Benjamini-Hochberg FDR.

Functional features were ranked by effect size and *P*-value, and the top features were visualized using bar plots and volcano plots. Logistic regression was used to estimate odds ratios and confidence intervals for selected pathways, and k-nearest neighbor networks were constructed from Bray-Curtis similarity matrices to evaluate sample-level functional relationships. All statistical analyses and visualizations were performed in R (v.4.1.3) using packages including ggplot2, vegan, limma, igraph, and pheatmap.

## RESULTS

### Baseline microbiome composition

At baseline, Shannon alpha-diversity (*P* > 0.18) and beta-diversity analyses (PERMANOVA *R*² = 0.14, *P* = 0.43) showed no group clustering (Fig. S1A and S1B) with broadly similar genus-level composition across all groups (Fig. S1C through S1G and Tables 1 and 2). Pairwise ASVs were highly concordant (*r* = 0.90–0.93) across groups and were highly positive (Fig. S2 and S3), indicating a stable and comparable GMB at baseline. These data show that subsequent differences reflect treatment or intervention effects rather than baseline variability.

**TABLE 1:** Percent read abundance of taxa (vs baseline PBS-UA group)^*a*^

Taxa	AmAc-UA	PBS-VWR	AmAc-VWR
*Akkermansia*	Up	Up	Down
*Lachnospiraceae* NK4A136	Down	Down	Down
*Muribaculaceae* ASV1	Down	Down	Down
*Muribaculaceae* ASV2	Down	Down	Down
*Muribaculaceae* ASV4	Down	Down	Down
*Muribaculaceae* ASV6	Down	Down	Down
*Bacteroides*	Down	Down	Up
*Muribaculaceae* ASV9	None	Down	None
*Muribaculaceae* ASV10	None	Down	None
*Muribaculaceae* ASV11	Up	Up	Up
*Muribaculaceae* ASV12	Up	Down	Down
*Parasutterella*	None	Up	Up
*Lachnospiraceae* UCG-001	Down	Down	Down
*Lachnospiraceae* ASV22	Down	Down	Down
*Alistipes*	None	Up	Up
*Lachnospiraceae* ASV26	Down	Down	Down
*Muribaculaceae* ASV34	Down	Down	Up
*Muribaculaceae* ASV16	None	None	Down
*Colidextribacter*	None	Down	None
*Ruminococcus*	Up	None	None

^*a*^ AmAc-UA, ammonium acetate usual activity mice; AmAc-VWR, ammonium acetate voluntary wheel running mice; ASV, amplicon sequence variant; PBS-UA, phosphate-buffered saline usual activity mice; PBS-VWR, phosphate-buffered saline voluntary wheel running mice. *N* = 8 in each group. The numerical after ASV indicates unique identification for mapping but has no biological significance.

**TABLE 2:** Differential abundance of taxa (highest to lowest at baseline)^*a*^

Taxa	Mouse groups
*Akkermansia*	PBS-VWR, AmAc-UA, PBS-UA, AmAc-VWR
*Lachnospiraceae* NK4A136	PBS-UA, PBS-VWR, AmAc-UA, AmAc-VWR
*Bacteroides*	AmAc-VWR, PBS-UA, AmAc-UA, PBS-VWR
*Parasutterella*	AmAc-VWR, PBS-VWR, PBS-UA, AmAc-UA
*Colidextribacter*	AmAc-VWR, PBS-UA, AmAc-UA, PBS-VWR
*Anaeroplasma*	AmAc-VWR, AmAc-UA, PBS-UA, PBS-VWR
*Eubacterium siraeum*	AmAc-VWR, AmAc-UA, PBS-UA, PBS-VWR
*Ruminococcus gnavus*	AmAc-UA, PBS-UA, AmAc-VWR, PBS-VWR
*Eubacterium ventriosum*	PBS-VWR, AmAc-UA, PBS-UA, AmAc-VWR
*Lachnospiraceae* UCG-001	PBS-UA, PBS-VWR, AmAc-UA, AmAc-VWR

^*a*^ AmAc-UA, ammonium acetate usual activity mice; AmAc-VWR, ammonium acetate voluntary wheel running mice; PBS-UA, phosphate-buffered saline usual activity mice; PBS-VWR, phosphate-buffered saline voluntary wheel running mice. *N* = 8 in each group.

### Effect of hyperammonemia before intervention

**Fig 1:**
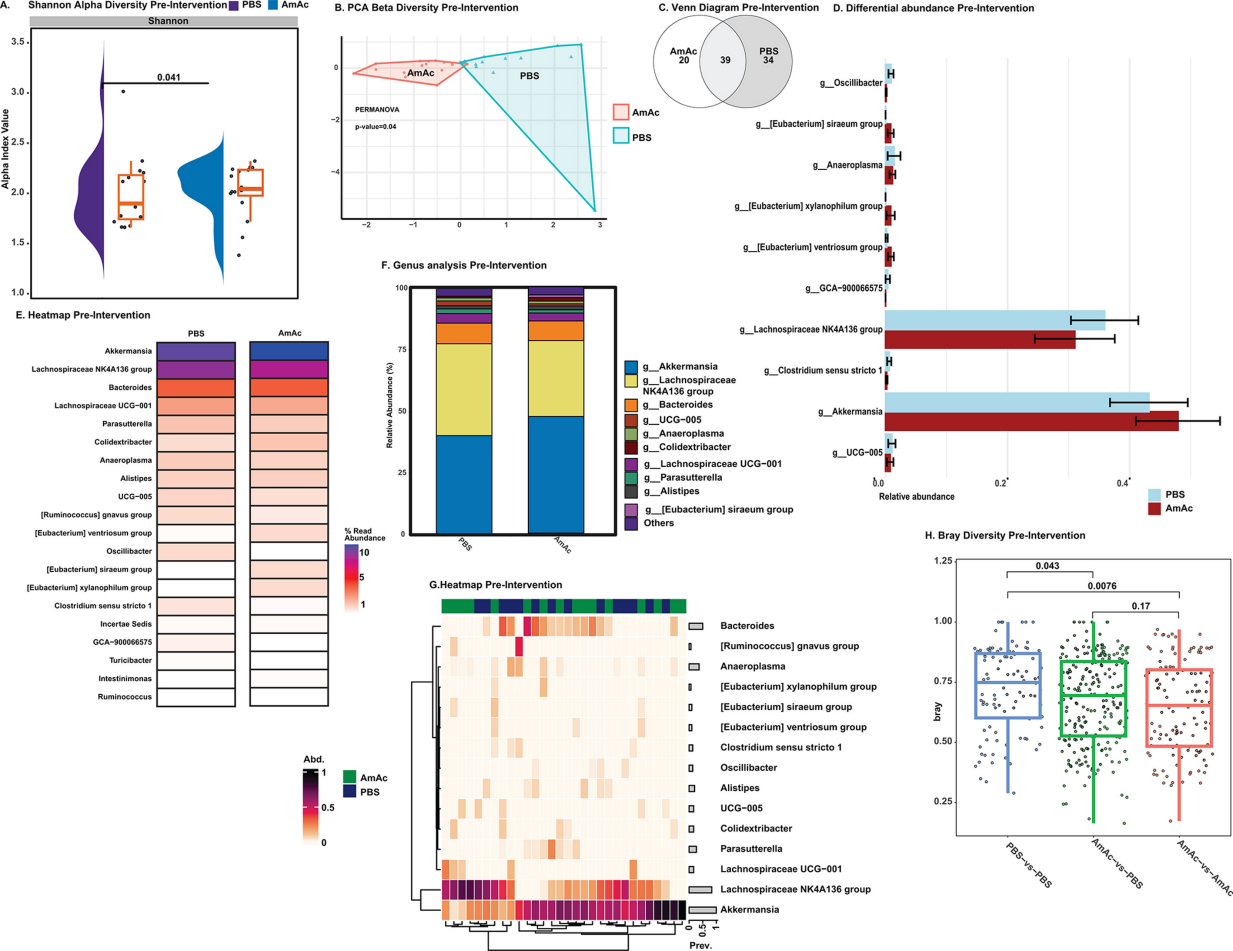
Disrupted gut microbiome architecture by hyperammonemic stress. Studies were performed in male C57BL/6J mice aged 8–10 weeks treated with either sterile PBS (vehicle) or 2.5 mmol/kg/day AmAc for 42 days. Two weeks post-pump placement (and before intervention), stool samples were collected, sequenced, and then analyzed. (**A**) Violin plot depicting alpha-diversity (Shannon) between PBS- and AmAc-treated mice is shown, indicating lower alpha-diversity in AmAc-treated mice (*P* = 0.04) at the pre-intervention time point. (**B**) CCA plot depicting distinct clustering of PBS and AmAc-treated mice (PERMANOVA *R*^2^ = 0.29, *P* = 0.04). (**C**) Venn diagram showing unique (PBS: 34 and AmAc: 20) and shared (39) bacterial taxa. (**D**) Bar graphs showing significantly differentially abundant genera between groups with a *P* < 0.05. (**E**) Heatmap of overall genus abundance. (**F**) Alluvial plot depicting the overall relative abundance of bacterial genera. (**G**) Unsupervised heatmap of significantly differentially abundant genera at baseline. (**H**) Bray-Curtis beta-diversity is shown as boxplots comparing group differences. Data: mean ± SD. Statistical analyses: for comparisons between two groups (panels A, D, and H), White’s non-parametric test with pooled variance and bootstrapped *P*-values was applied. PERMANOVA was used for beta-diversity analysis in panel B. *n* = 8 mice per group.

Two weeks of HAS reduced Shannon diversity compared with PBS controls (*P* = 0.04) (Fig. 1A). There was clear separation in beta-diversity between PBS and AmAc groups (PERMANOVA *R*^2^ = 0.29, *P* = 0.04) (Fig. 1B), indicating an early compositional difference. One sample showed separation on CCA plots but did not differ in sequencing depth or quality metrics and was retained, consistent with expected inter-individual variability in microbiome data sets. Core microbiota analysis revealed 39 shared taxa, while PBS mice had 34 unique taxa vs 20 in AmAc (Fig. 1C). Ten taxa differed significantly, with enrichment of *Eubacterium siraeum, Eubacterium xylanophilum, Eubacterium ventriosum*, and *Akkermansia* in AmAc mice (Fig. 1D and Tables 3 and 4). Heatmaps and alluvial plots supported these compositional differences (Fig. 1E through G). AmAc-treated mice had higher within-group variability based on Bray-Curtis dissimilarity (*P* = 0.04) (Fig. 1H). Together, these data show that HAS drives early disruption of microbial diversity, composition, and stability.

**TABLE 3:** Differential abundance of taxa pre-intervention in PBS vs AmAc mice^*a*^

Taxa	Group comparisons of abundance
*Oscillibacter*	PBS > AmAc
*Eubacterium siraeum* group	AmAc > PBS
*Anaeroplasma*	PBS > AmAc
*Eubacterium xylanophilum*	AmAc > PBS
*Eubacterium ventriosum*	AmAc > PBS
GCA-900066575	PBS > AmAc
*Lachnospiraceae* NK4A136	PBS > AmAc
*Clostridium sensu stricto 1*	PBS > AmAc
*Akkermansia*	AmAc > PBS
UCG-005	PBS > AmAc

^*a*^ AmAc, ammonium acetate; PBS, phosphate-buffered saline.

**TABLE 4:** Percent read abundance of taxa pre-intervention as compared to PBS^*a*^

Taxa	AmAc
*Akkermansia*	Up
*Lachnospiraceae* NK4A136	Down
*Bacteroides*	None
*Lachnospiraceae* UCG-001	None
*Parasutterella*	None
*Colidextribacter*	None
*Anaeroplasma*	None
*Alistipes*	None
UCG-005	None
*Ruminococcus gnavus*	None
*Eubacterium ventriosum*	Up
*Oscillibacter*	Down
*Eubacterium siraeum*	Up
*Eubacterium xylanophilum*	Up
*Clostridium sensu stricto 1*	Down
*Incertae sedis*	None
GCA-900066575	Down
*Turicibacter*	None
*Intestinimonas*	None
*Ruminococcus*	None

^*a*^ AmAc, ammonium acetate; PBS, phosphate-buffered saline.

### Post-intervention changes in microbial composition

**Fig 2:**
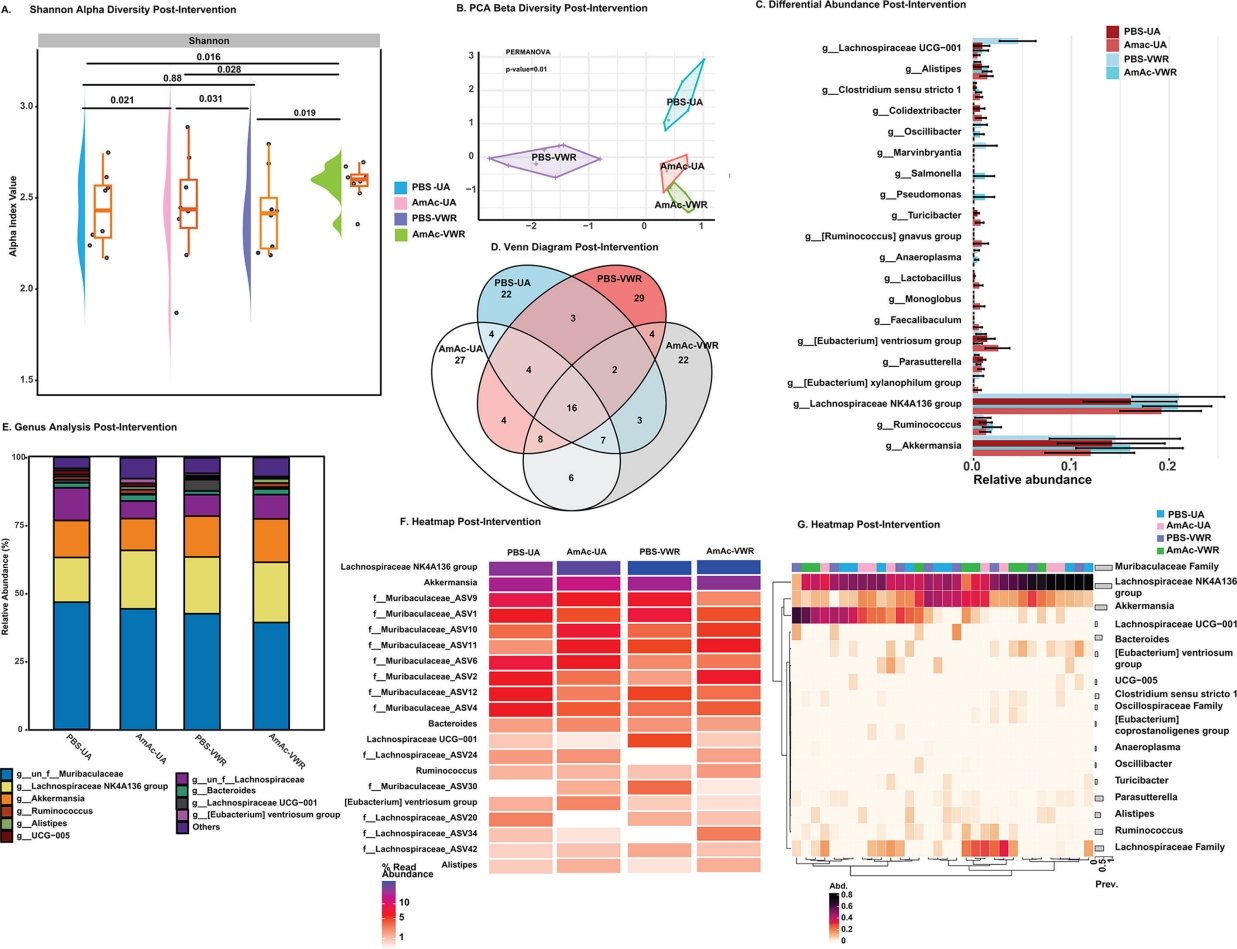
Restoration of hyperammonemic stress-induced gut microbiome alterations by exercise. Studies were performed in male C57BL/6J mice aged 8–10 weeks treated with either PBS or 2.5 mmol/kg/day AmAc for 42 days. Two weeks post-pump placement, mice were randomized to interventions (UA or VWR) for 4 weeks. At the end of the intervention period (post), stool samples were collected, sequenced, and analyzed. (**A**) Violin plot of alpha-diversity (Shannon) across four groups. (**B**) CCA plot showing group clustering (PERMANOVA *R*^2^ = 0.38, *P* = 0.01). (**C**) Bar graphs showing statistically differential genus-level abundance between all groups, with *P* < 0.05. (**D**) Venn diagram showing shared and unique bacterial taxa. (**E**) Alluvial plot of genus-level abundance between groups. (**F**) Heatmap of genus-level abundance. (**G**) Unsupervised heatmap of differentially abundant genera across groups. Data: mean ± SD. Statistical analyses: for comparisons between two groups (panels A and C), White’s non-parametric test with pooled variance and bootstrapped *P*-values was applied. PERMANOVA was used for beta-diversity analysis in panel B. *n* = 8 mice per group.

Following intervention, alpha-diversity differed among groups, with the highest values in AmAc-VWR and the lowest in PBS-UA mice (*P* < 0.05 for both), while PBS-UA and PBS-VWR were similar (*P* = 0.88) (Fig. 2A). Beta-diversity analysis (PERMANOVA *R*^2^ = 0.38, *P* = 0.01) showed significant separation among groups (Fig. 2B). Differential abundance analysis identified group-specific microbial shifts and multiple taxa changes across intervention groups (Fig. 2C and D and Table 5). Notably, HAS was associated with depletion of *Lachnospiraceae*, which was partially restored by VWR (Fig. 2E through G and Table 6). These findings demonstrate that both HAS and VWR remodel the gut microbiome, with exercise partially reversing dysbiosis and promoting microbial diversity in a context-dependent manner.

**TABLE 5:** Differential abundance of taxa post-intervention (most abundant to least abundant)^*a*^

Taxa	Groups
*Lachnospiraceae* UCG-001	PBS-VWR, PBS-UA, AmAc-VWR, AmAc-UA
*Alistipes*	AmAc-VWR, AmAc-UA, PBS-UA, PBS-VWR
*Clostridium sensu stricto 1*	AmAc-UA, AmAc-VWR, PBS-VWR, PBS-UA
*Colidextribacter*	AmAc-UA, PBS-UA, PBS-VWR, AmAc-VWR
*Oscillibacter*	PBS-VWR, AmAc-VWR, PBS-UA, AmAc-UA
*Marvinbryantia*	PBS-VWR (all remaining the same)
*Salmonella*	AmAc-VWR (all remaining the same)
*Pseudomonas*	AmAc-VWR (all remaining the same)
*Turicibacter*	AmAc-UA, PBS-UA, (remaining the same)
*Ruminococcus gnavus* group	AmAc-UA (remaining the same)
*Anaeroplasma*	AmAc-VWR, PBS-VWR, PBS-UA, AmAc-UA
*Lactobacillus*	AmAc-UA, PBS-UA, PBS-VWR, AmAc-VWR
*Monoglobus*	AmAc-UA (remaining the same)
*Faecalibacterium*	AmAc-UA (remaining the same)
*Eubacterium ventriosum* group	AmAc-UA, PBS-UA, PBS-VWR, AmAc-VWR
*Parasutterella*	PBS-UA, AmAc-UA, AmAc-VWR, PBS-VWR
*Eubacterium xylanophilum*	PBS-VWR, AmAc-UA (remaining same)
*Lachnospiraceae* NK4A136	PBS-VWR, AmAc-VWR, AmAc-UA, PBS-UA
*Ruminococcus*	AmAc-VWR, PBS-UA, AmAc-UA, PBS-VWR
*Akkermansia*	AmAc-VWR, PBS-VWR, PBS-UA, AmAc-UA

^*a*^ AmAc-UA, ammonium acetate usual activity mice; AmAc-VWR, ammonium acetate voluntary wheel running mice; PBS-UA, phosphate-buffered saline usual activity mice; PBS-VWR, phosphate-buffered saline voluntary wheel running mice. *N* = 8 in each group.

**TABLE 6:** Percent read abundance post-intervention (compared to PBS-UA)^*a*^

Taxa	AmAc-UA	PBS-VWR	AmAc-VWR
*Lachnospiraceae* NK4A136 group	Up	Up	Up
*Akkermansia*	Down	Up	Up
*Muribaculaceae* ASV9	None	None	Down
*Muribaculaceae* ASV1	None	None	None
*Muribaculaceae* ASV10	Up	None	Up
*Muribaculaceae* ASV11	Up	Up	Up
*Muribaculaceae* ASV6	None	Down	Down
*Muribaculaceae* ASV2	Down	Down	None
*Muribaculaceae* ASV12	Down	None	Down
*Muribaculaceae* ASV4	Down	Down	Down
*Bacteroides*	None	None	None
*Lachnospiraceae* UCG-001	Down	Up	None
*Lachnospiraceae* ASV24	None	Down	None
*Ruminococcus*	None	None	Up
*Muribaculaceae* ASV30	Up	Up	Up
*Eubacterium ventriosum*	Up	Down	Down
*Lachnospiraceae* ASV20	Down	Down	Down
*Lachnospiraceae* ASV34	Down	Down	Up
*Lachnospiraceae* ASV42	None	None	None
*Alistipes*	Up	Down	Up

^*a*^ AmAc-UA, ammonium acetate usual activity mice; AmAc-VWR, ammonium acetate voluntary wheel running mice; ASV, amplicon sequence variant; PBS-UA, phosphate-buffered saline usual activity mice; PBS-VWR, phosphate-buffered saline voluntary wheel running mice. *N* = 8 in each group. The numerical after ASV indicates unique identification for mapping but has no biological significance.

### Longitudinal changes in microbial composition

**Fig 3:**
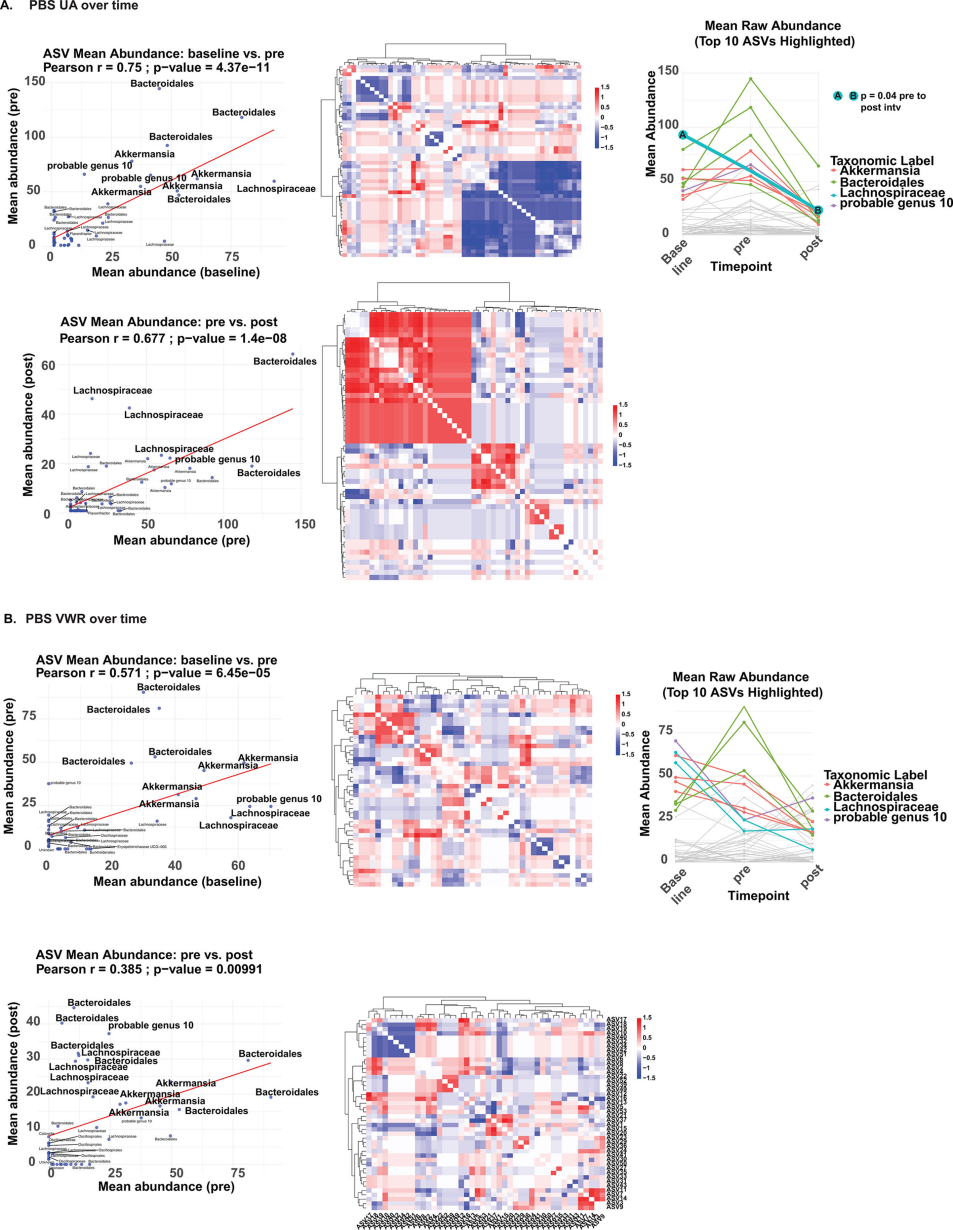
Temporal dynamics of the gut microbiome by exercise. Male C57BL/6J mice aged 8–10 weeks were treated with either PBS or 2.5 mmol/kg/day AmAc for 42 days (treatment period). Two weeks after the osmotic pumps were installed, mice were randomized to UA or VWR for 4 weeks (intervention period). Stool samples were collected at three time points: baseline (prior to treatment or intervention), pre-intervention (2 weeks post-treatment), and post-intervention (4 weeks post-intervention). The samples were then analyzed at the ASV level. (**A**) PBS-UA group: scatter plots of ASV mean abundance (baseline vs pre, pre vs post), ASV-ASV network plots at each time point, and time-course line plots tracking the top 10 most abundant ASVs. (**B**) PBS-VWR group: scatter plots of ASV mean abundance (baseline vs pre, pre vs post), ASV-ASV network plots at each time point, and time-course line plots tracking the top 10 most abundant ASVs. Data: mean ± SD. Statistical analyses: Pearson correlation was used to assess similarity in mean ASV abundance across time points (pre-intervention vs baseline and post-intervention vs pre-intervention) and to construct ASV-ASV co-abundance networks. For time-course plots of the top 10 ASVs (right), White’s non-parametric test with pooled variance and 1,000 bootstraps was used to compare abundance across time points. *P*-values were adjusted using the Benjamini-Hochberg method. *P < 0.05. *n* = 8 mice per group.

**Fig 4:**
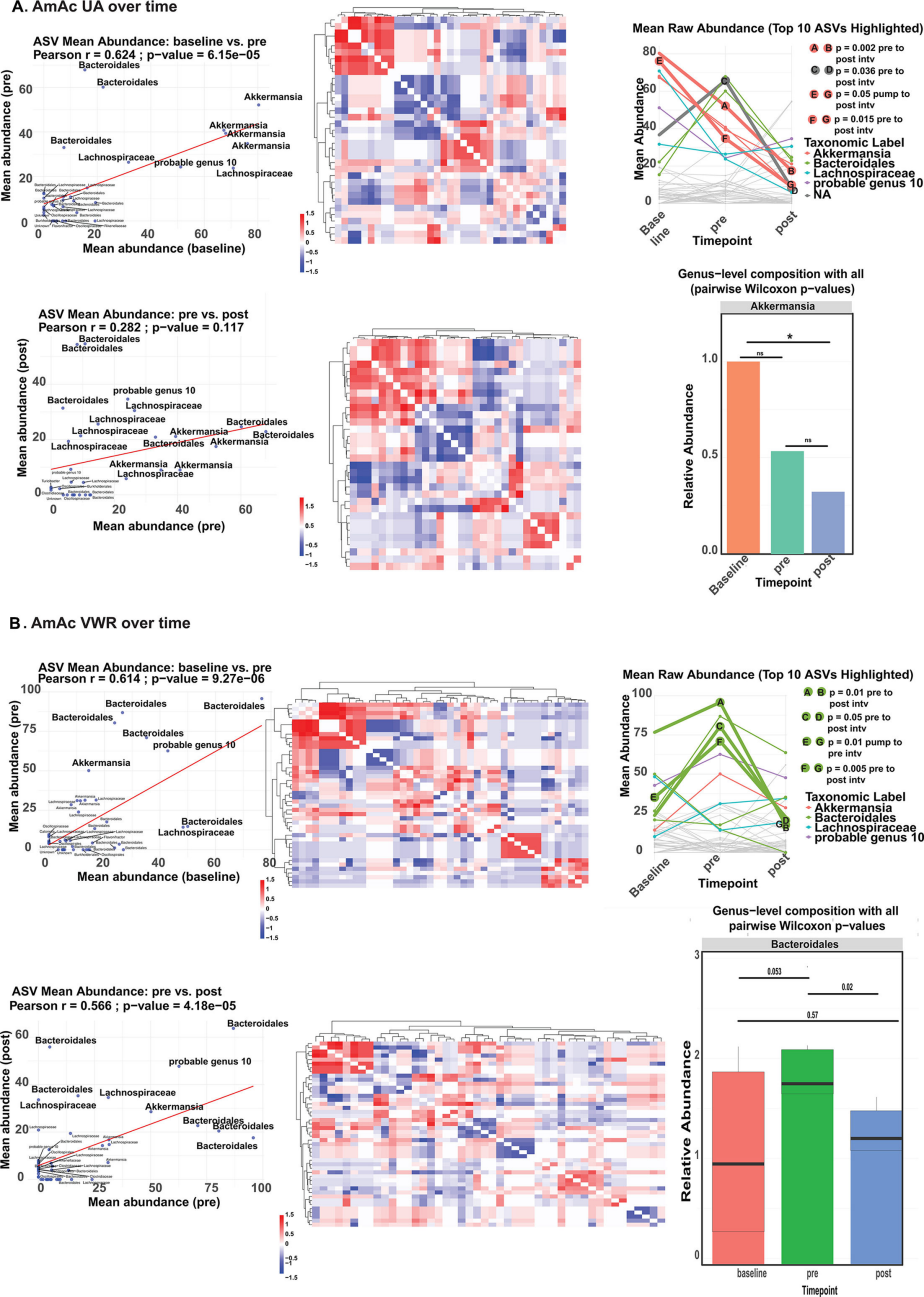
Temporal dynamics of the altered gut microbiome during exercise under hyperammonemic stress. Male C57BL/6J mice aged 8–10 weeks were treated with either PBS or 2.5 mmol/kg/day AmAc for 42 days (treatment period). Two weeks after the osmotic pumps were installed, mice were randomized to UA or VWR for 4 weeks (intervention period). Stool samples were collected at three time points: baseline (prior to treatment or intervention), pre-intervention (2 weeks post-treatment), and post-intervention (4 weeks post-intervention). The samples were then analyzed at the ASV level. (**A**) AmAc-UA group: scatter plots of ASV mean abundance (baseline vs pre, pre vs post), ASV-ASV network plots at each time point, time-course line plots tracking the top 10 most abundant ASVs, and genus-level abundance comparisons across time points for the most abundant genera. (**B**) AmAc-VWR group: scatter plots of ASV mean abundance (baseline vs pre, pre vs post), ASV-ASV network plots at each time point, time-course line plots tracking the top 10 most abundant ASVs, and time-course tracking of the top 10 ASVs and genus-level comparisons of dominant taxa over time. Data: mean ± SD. Statistical analyses: Pearson correlation was used to assess similarity in mean ASV abundance across time points (pre-intervention vs baseline and post-intervention vs pre-intervention) and to construct ASV-ASV co-abundance networks. For time-course plots of the top 10 ASVs, White’s non-parametric test with pooled variance and 1,000 bootstraps was used to compare abundance across time points. *P*-values were adjusted using the Benjamini-Hochberg method. *P<0.05. *n* = 8 mice per group.

In PBS-UA mice, the microbiome remained stable across all time points, with strong correlations (*r* = 0.75–0.67, *P* < 0.001) among baseline, pre-, and post-intervention samples (Fig. 3A and Fig. S4A and B). In contrast, PBS-VWR mice showed stability from baseline to pre-intervention but diverged following exercise (*r* = 0.385, *P* = 0.0091), showing exercise-induced restructuring under physiological conditions (Fig. 3B and Fig. S4C and D). AmAc-UA mice exhibited progressive instability, with reduced correlation over time (*r* = 0.624 to *r* = 0.282), accompanied by compositional shifts including decreased *Akkermansia* (Fig. 4A and Fig. S4E and F). In contrast, AmAc-VWR mice retained moderate correlation (*r* = 0.566, *P* < 0.001) between pre- and post-intervention samples (Fig. 4B and Fig. S4G and H), indicating preservation of microbial structure despite hyperammonemic stress. These findings demonstrate that HAS drives progressive microbiome instability, whereas VWR supports a favorable microbiome and mitigates longitudinal disruption despite HAS.

### Alterations in microbiome in different groups across time points

Pre-intervention comparisons revealed reduced correlation between AmAc-UA and PBS-UA groups (*r* = 0.70, *P* < 0.001), accompanied by disruption of key microbial associations involving *Muribaculaceae*, *Akkermansia*, and *Lachnospiraceae* (Fig. S5A). In contrast, PBS-VWR and PBS-UA groups remained highly correlated (*r* = 0.72, *P* < 0.001), indicating preserved network structure under control conditions (Fig. S5B). Comparisons involving mice with HAS showed consistently lower correlations, including AmAc-VWR vs PBS-VWR (*r* = 0.69, *P* < 0.001) and AmAc-VWR vs AmAc-UA (*r* = 0.71, *P* < 0.001), suggesting persistent network perturbation despite exercise (Fig. S5C and D). Post-intervention, correlations between groups remained reduced, particularly in comparisons involving AmAc-treated mice, indicating sustained disruption of microbial network structure (Fig. S6A through D).

Baseline-to-post-intervention comparisons further reinforced these patterns. PBS-UA mice maintained strong correlation (*r* = 0.90, *P* < 0.001) over time (Fig. S7A), indicating sustained microbial stability. In contrast, AmAc-UA mice showed reduced correlation (*r* = 0.58, *P* < 0.001) with disrupted network structure (Fig. S7B), reflecting progressive instability during HAS. Both PBS-VWR and AmAc-VWR groups exhibited lower correlations (*r* = 0.45–0.50, *P* < 0.001) compared with PBS-UA (Fig. S7C and D), consistent with exercise-induced restructuring of the microbiome. Notably, AmAc-VWR retained greater coherence than AmAc-UA, suggesting partial preservation of microbiome structure despite HAS.

**Fig 5:**
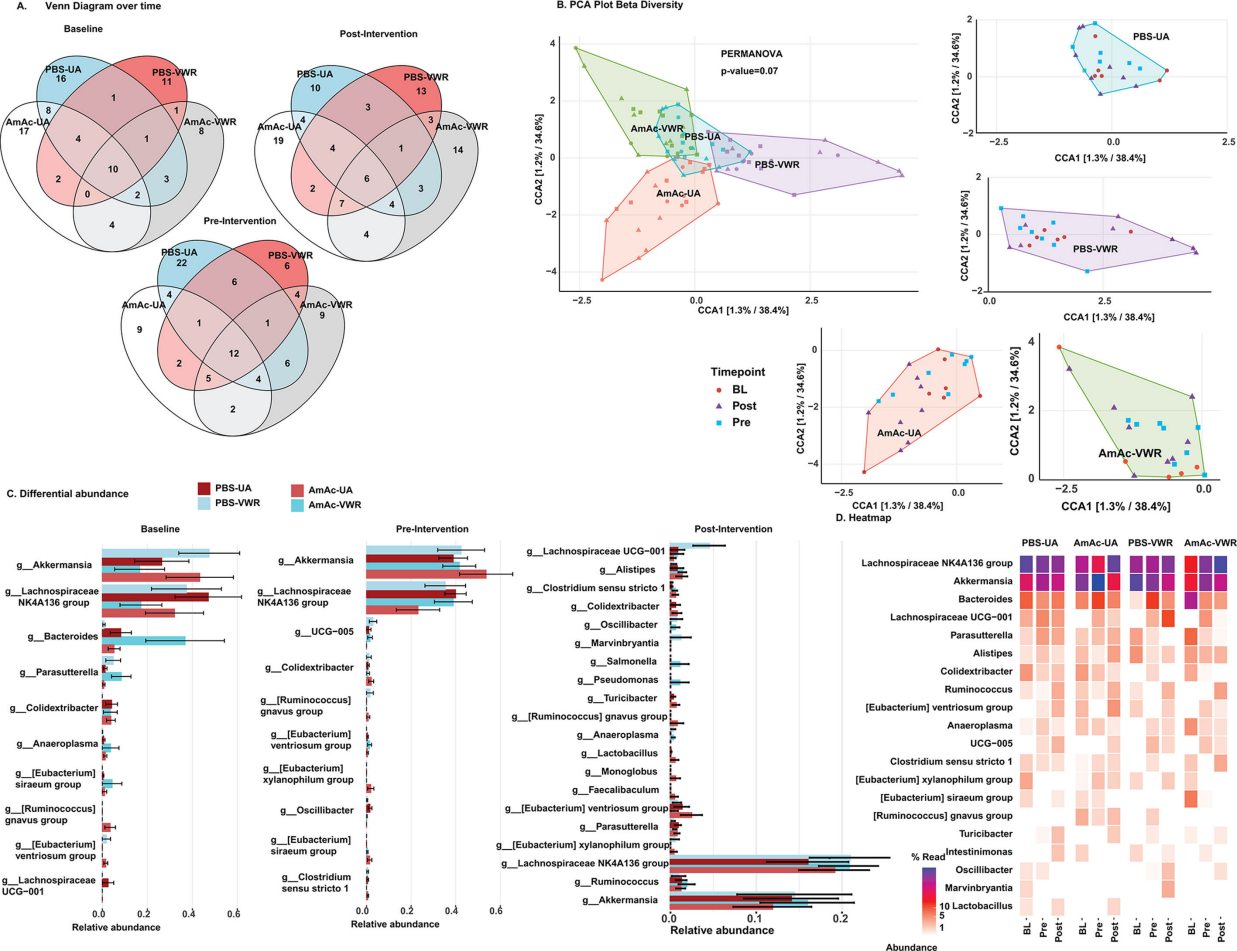
Independent effects of hyperammonemic stress and exercise on the gut microbiome. Male C57BL/6J mice aged 8–10 weeks were treated with either PBS or 2.5 mmol/kg/day AmAc for 42 days (treatment period). Two weeks after the osmotic pumps were installed, mice were randomized to UA or VWR for 4 weeks (intervention period). Stool samples were collected at three time points: baseline (prior to treatment or intervention), pre-intervention (2 weeks post-treatment), and post-intervention (4 weeks post-intervention). The samples were then sequenced and analyzed. (**A**) Venn diagrams of shared and unique genera across time points and groups. (**B**) CCA plot showing group clustering of genus-level beta-diversity across all groups and at all time points, with distribution of each group. PERMANOVA overall *R*^2^ = 0.23, *P* = 0.07, PBS-UA *R*^2^ = 0.19, *P* = 0.03, PBS-VWR *R*^2^ = 0.13, *P* = 0.05, AmAc-UA *R*^2^ = 0.26, *P* = 0.02, AmAc-VWR *R*^2^ = 0.31, *P* = 0.06. (**C**) Bar plot showing group- and time-specific genera. (**D**) Heatmap of genus-level abundance data: mean ± SD. Statistical analyses: PERMANOVA was used to analyze beta-diversity in panel B. For comparisons between two groups (panel C), White’s non-parametric test with pooled variance and bootstrapped *P*-values was applied. *n* = 8 mice per group.

The number of shared core taxa at baseline (*n* = 10 taxa) decreased post-intervention (*n* = 6) across all four groups, indicating reduced microbial overlap over time (Fig. 5A and Tables 7–9). This reduction was more pronounced during HAS, with fewer shared taxa between AmAc-UA and PBS-UA groups post-intervention. In contrast, VWR partially preserved shared taxa, with increased overlap observed between PBS-VWR and AmAc-VWR groups post-intervention. Consistent with these findings, beta-diversity analysis (Fig. 5B) showed clustering in PBS-UA mice, with greater divergence in the VWR and HAS groups PERMANOVA Overall *R*^2^ = 0.23, *P* = 0.07; PBS-UA *R*^2^ = 0.19, *P* = 0.03; PBS-VWR *R*^2^ = 0.13, *P* = 0.05; AmAc-UA *R*^2^ = 0.26, *P* = 0.02; AmAc-VWR *R*^2^ = 0.31, *P* = 0.06). Persistently abundant taxa across time points included *Akkermansia*, *Lachnospiraceae* NK4A136, *Colidextribacter*, *Ruminococcus gnavus*, and *Eubacterium ventriosum* (Fig. 5C and Tables 10–13). Taxa such as *Lachnospiraceae* UCG-005, *Clostridium sensu stricto 1*, and *Turicibacter* were altered during HAS, while VWR partially restored the abundance of several taxa. Together, these longitudinal analyses show that HAS drives progressive microbiome destabilization with lower shared microbial features over time, while VWR preserves or restores core taxa, partially stabilizing microbiome composition under metabolic stress (Fig. 5D).

**TABLE 7:** Differential abundance baseline (most abundant to least abundant taxa)^*a*^

Taxa	Groups
*Akkermansia*	PBS-VWR, AmAc-UA, PBS-UA, AmAc-VWR
*Lachnospiraceae* NK4A136	PBS-UA, PBS-VWR, AmAc-UA, AmAc-VWR
*Bacteroides*	AmAc-VWR, PBS-UA, AmAc-UA, PBS-VWR
*Parasutterella*	AmAc-VWR, PBS-VWR, PBS-UA, AmAc-UA
*Colidextribacter*	PBS-UA, AmAc-UA, AmAc-VWR, PBS-VWR
*Anaeroplasma*	AmAc-VWR, AmAc-UA, PBS-UA, PBS-VWR
*Eubacterium siraeum* group	AmAc-VWR, AmAc-UA, PBS-UA, PBS-VWR
*Ruminococcus gnavus* group	AmAc-UA (all others low)
*Eubacterium ventriosum*	PBS-VWR, AmAc-UA (all others same)
*Lachnospiraceae* UCG-001	PBS-UA (all others same)

^*a*^ AmAc-UA, ammonium acetate usual activity mice; AmAc-VWR, ammonium acetate voluntary wheel running mice; PBS-UA, phosphate-buffered saline usual activity mice; PBS-VWR, phosphate-buffered saline voluntary wheel running mice. *N* = 8 in each group.

**TABLE 8:** Differential abundance pre-intervention (most abundant to least abundant taxa)^*a*^

Taxa	Groups
*Akkermansia*	AmAc-UA, PBS-VWR, AmAc-VWR, PBS-UA
*Lachnospiraceae* NK4A136	PBS-UA, AmAc-VWR, PBS-VWR, AmAc-UA
UCG-005	PBS-VWR, AmAc-VWR, PBS-UA, AmAc-UA
*Colidextribacter*	AmAc-UA, PBS-VWR (others same)
*Ruminococcus gnavus*	PBS-VWR, AmAc-UA (others same)
*Eubacterium ventriosum*	AmAc-VWR, PBS-UA, AmAc-UA, PBS-VWR
*Eubacterium xylanophilum*	AmAc-UA (all others same)
*Oscillibacter*	PBS-UA, PBS-VWR, AmAc-VWR, AmAc-UA
*Eubacterium siraeum*	AmAc-UA, AmAc-VWR (all others same)
*Clostridium sensu stricto*	PBS-VWR, PBS-UA, AmAc-UA, AmAc-VWR

^*a*^ AmAc-UA, ammonium acetate usual activity mice; AmAc-VWR, ammonium acetate voluntary wheel running mice; PBS-UA, phosphate-buffered saline usual activity mice; PBS-VWR, phosphate-buffered saline voluntary wheel running mice. *N* = 8 in each group.

**TABLE 9:** Differential abundance post-intervention (most abundant to least abundant taxa)^*a*^

Taxa	Groups
*Lachnospiraceae* UCG-001	PBS-VWR, PBS-UA, AmAc-VWR, AmAc-UA
*Eubacterium ventriosum*	AmAc-UA, PBS-UA, PBS-VWR, AmAc-VWR
*Alistipes*	AmAc-VWR, AmAc-UA, PBS-UA, PBS-VWR
*Parasutterella*	PBS-UA, AmAc-UA, AmAc-VWR, PBS-VWR
*Clostridium sensu stricto 1*	AmAc-VWR, AmAc-UA, PBS-UA, PBS-VWR
*Turicibacter*	PBS-UA, AmAc-UA, AmAc-VWR, PBS-VWR
*Oscillibacter*	PBS-VWR, AmAc-VWR, PBS-UA, AmAc-UA
*Marvinbryantia*	PBS-VWR (all others same)
*Colidextribacter*	PBS-UA, AmAc-UA, (all others same)
*Ruminococcus gnavus*	Am- (all others same)

^*a*^ AmAc-UA, ammonium acetate usual activity mice; AmAc-VWR, ammonium acetate voluntary wheel running mice; PBS-UA, phosphate-buffered saline usual activity mice; PBS-VWR, phosphate-buffered saline voluntary wheel running mice. *N* = 8 in each group.

**TABLE 10:** Differential abundance of taxa over time^*a*^

Taxa	Baseline	Pre-intervention	Post-intervention
*Akkermansia*	PBS-VWR AmAc-UA PBS-UA AmAc-VWR	AmAc-UA PBS-VWR AmAc-VWR PBS-UA	N/A^*b*^
*Lachnospiraceae* NK4A136	PBS-UA PBS-VWR AmAc-UA AmAc-VWR	PBS-UA AmAc-VWR PBS-VWR AmAc-UA	N/A
*Bacteroides*	AmAc-VWR PBS-UA AmAc-UA PBS-VWR	N/A	N/A
*Parasutterella*	AmAc-VWR PBS-VWR PBS-UA AmAc-UA	N/A	PBS-UA AmAc-UA AmAc-VWR PBS-VWR
*Colidextribacter*	PBS-UA AmAc-UA AmAc-VWR PBS-VWR	AmAc-UA PBS-VWR Others same	PBS-UA AmAc-UA Others same
*Anaeroplasma*	AmAc-VWR AmAc-UA PBS-UA PBS-VWR	N/A	N/A
*Eubacterium siraeum* group	AmAc-VWR AmAc-UA PBS-UA PBS-VWR	AmAc-UA AmAc-VWR Others same	N/A
*Ruminococcus gnavus* group	AmAc-UA Others same	PBS-VWR AmAc-UA Others same	AmAc-UA Others same
*Eubacterium ventriosum*	PBS-VWR AmAc-UA Others same	AmAc-VWR PBS-UA AmAc-UA PBS-VWR	AmAc-UA PBS-UA PBS-VWR AmAc-VWR
*Lachnospiraceae* UCG-001	PBS-UA Others same	N/A	PBS-VWR PBS-UA AmAc-VWR AmAc-UA
UCG-005	N/A	PBS-VWR AmAc-VWR PBS-UA AmAc-UA	N/A
*Eubacterium xylanophilum*	N/A	AmAc-UA Others same	N/A
*Oscillibacter*	N/A	PBS-UA PBS-VWR AmAc-VWR AmAc-UA	PBS-VWR AmAc-VWR PBS-UA AmAc-UA
*Clostridium sensu stricto 1*	N/A	PBS-VWR PBS-UA AmAc-UA AmAc-VWR	AmAc-VWR AmAc-UA PBS-UA PBS-VWR
*Alistipes*	N/A	N/A	AmAc-VWR AmAc-UA PBS-UA PBS-VWR
*Turicibacter*	N/A	N/A	PBS-UA AmAc-UA AmAc-VWR PBS-VWR
*Marvinbryantia*	N/A	N/A	PBS-VWR Others same

^*a*^ AmAc-UA, ammonium acetate usual activity mice; AmAc-VWR, ammonium acetate voluntary wheel running mice; PBS-UA, phosphate-buffered saline usual activity mice; PBS-VWR, phosphate-buffered saline voluntary wheel running mice. *N* = 8 in each group.

^*b*^ N/A, not applicable.

**TABLE 11:** Percent read abundance over time^*a*^

Taxa		PBS-UA	AmAc-UA	PBS-VWR	AmAc-VWR
*Lachnospiraceae* NK4A136 group	BL to pre BL to post Pre to post	Down Down Down	Down Up Up	None Up Down	Up Up Up
*Akkermansia*	BL to pre BL to post Pre to post	Up Up Up	Up Down Down	Down Down Down	Up Up Down
*Bacteroides*	BL to pre BL to post Pre to post	None None None	Up None Down	Up Up Down	Down Down Up
*Lachnospiraceae* UCG-001	BL to pre BL to post Pre to post	Up None Down	Up Up Down	Up Up Down	Up Up Down
*Parasutterella*	BL to pre BL to post Pre to post	Up Up None	Up Up Up	Up Up None	Down Down Down
*Alistipes*	BL to pre BL to post Pre to post	Up None Down	Down Up Up	Down Down Up	None None None
*Colidextribacter*	BL to pre BL to post Pre to post	Down Down None	Down Down Down	Down Down None	Up None Down
*Ruminococcus*	BL to pre BL to post Pre to post	Down Up Up	Down Up Up	Down Up Up	Up Down Down
*Eubacterium ventriosum*	BL to pre BL to post Pre to post	Up Up Up	Down Up Up	Up Up Up	Down None Up
*Anaeroplasma*	BL to pre BL to post Pre to post	Up None Down	None Down Down	Up Up None	Down Down None
UCG-005	BL to pre BL to post Pre to post	Up Up Up	None Up Up	Up Up Up	Up Up Down
*Clostridium sensu stricto 1*	BL to pre BL to post Pre to post	None None None	Up Up None	Up None Down	Down Up Up
*Eubacterium xylanophilum*	BL to pre BL to post Pre to post	Down Down None	Up Up Down	Down Up Up	Down Down None
*Eubacterium siraeum*	BL to pre BL to post Pre to post	Down Down Up	None Down Down	None None None	Down Down None
*Ruminococcus gnavus*	BL to pre BL to post Pre to post	None None None	Down None Up	Up None Down	Down None Up
*Turicibacter*	BL to pre BL to post Pre to post	None Up Up	None Up Up	Up None Down	None Up Up
*Intestinimonas*	BL to pre BL to post Pre to post	None Up Up	Down Down None	Down Down Up	Up None Down
*Oscillibacter*	BL to pre BL to post Pre to post	None Down Down	None None None	None Up Up	None Up Up
*Marvinbryantia*	BL to pre BL to post Pre to post	Down Down None	None None None	None Up Up	None None None
*Lactobacillus*	BL to pre BL to post Pre to post	Down Up Up	None Up Up	None None None	None None None

^*a*^ AmAc-UA, ammonium acetate usual activity mice; AmAc-VWR, ammonium acetate voluntary wheel running mice; BL, baseline; PBS-UA, phosphate-buffered saline usual activity mice; PBS-VWR, phosphate-buffered saline voluntary wheel running mice; Pre, pre-intervention; post, post-intervention. *N* = 8 in each group.

**TABLE 12:** Percent read abundance of taxa (post-intervention vs PBS-UA)^*a*^

Taxa	BL to Pre	BL to Post	Pre to Post
*Ruminococcus*	Down	Up	Up
*Eubacterium ventriosum*	Down	Up	Up
UCG-005	Up	Up	Down
*Lachnospiraceae* UCG-001	Up	Up	Up
*Parasutterella*	Down	Down	Down
*Colidextribacter*	Down	Down	Up
*Eubacterium xylanophilum*	Down	Up	Up
*Eubacterium siraeum*	Down	Down	Up
*Bacteroides*	Down	Down	Down
*Alistipes*	Down	Down	Up
*Oscillibacter*	Up	Up	Down
*Intestinimonas*	Down	Down	Up
*Turicibacter*	Down	Up	Up
*Akkermansia*	Down	Down	Down
*Clostridium sensu stricto*	Down	Up	Up
*Marvinbryantia*	Down	Up	Up
*Pseudomonas*	None	Up	Up
*Ruminococcus gnavus*	Up	Down	Down
*Lachnospiraceae* NK4A136	Down	Down	Up
*Anaeroplasma*	Up	Down	Down

^*a*^ BL, baseline; PBS-UA, phosphate-buffered saline usual activity; Post, post-intervention; Pre, pre-intervention.

**TABLE 13:** Percent read abundance over time across groups^*a*^

Taxa		PBS-UA	AmAc-UA	PBS-VWR	AmAc-VWR
*Akkermansia*	BL to pre BL to post Pre to post	None None None	Down Down Down	Down Down Down	None None None
*Lachnospiraceae* NK4A136	BL to pre BL to post Pre to post	Down Down None	Up None Down	Down None Up	None Up Up
*Muribaculaceae* ASV1	BL to pre BL to post Pre to post	None None None	Up None Down	Up Up None	None Up Up
*Muribaculaceae* ASV2	BL to pre BL to post Pre to post	None None None	Up Down Down	Down Up Up	Up Up Down
*Muribaculaceae* ASV4	BL to pre BL to post Pre to post	None None None	Up Up Down	None None None	Up Up Down
*Muribaculaceae* ASV6	BL to pre BL to post Pre to post	None Up Up	Up Up None	Up Up Down	Up Up Down
*Bacteroides*	BL to pre BL to post Pre to post	Down Down Down	Up None Down	Up None Down	Down Down Down
*Muribaculaceae* ASV9	BL to pre BL to post Pre to post	Up Up Up	Up Up Up	Up Up Up	Up Up None
*Muribaculaceae* ASV10	BL to pre BL to post Pre to post	Down None Up	None Up Up	Up Up None	Up Up None
*Muribaculaceae* ASV11	BL to pre BL to post Pre to post	Up Up Up	None Up Up	None Up Up	None Up Up
*Muribaculaceae* ASV12	BL to pre BL to post Pre to post	None Up Up	Down None Up	Up Up Up	Up Up Up
*Parasutterella*	BL to pre BL to post Pre to post	None None None	None None None	Down Down Down	Down Down Down
*Lachnospiraceae* UCG-001	BL to pre BL to post Pre to post	Up None Down	Up Up Down	Up Up Up	Up Up Down
*Lachnospiraceae* ASV22	BL to pre BL to post Pre to post	None None None	Up None Down	Up Up None	Up Up None
*Alistipes*	BL to pre BL to post Pre to post	Down None Up	None None None	Down Down Down	Down Down Up
*Lachnospiraceae* ASV26	BL to pre BL to post Pre to post	Up Up None	Up Up Up	Up None Down	None Up Up
*Muribaculaceae* ASV34	BL to pre BL to post Pre to post	Down Down Down	Down Up Up	Up Up Up	None Down Down
*Muribaculaceae* ASV16	BL to pre BL to post Pre to post	Down Down Down	Up Down Down	Up Up None	None None None
*Colidextribacter*	BL to pre BL to post Pre to post	Down Down Up	Up None Down	None None None	Down Down Down
*Ruminococcus*	BL to pre BL to post Pre to post	None Up Up	Down None Up	Down Up Up	None Up Up

^*a*^ AmAc-UA, ammonium acetate usual activity mice; AmAc-VWR, ammonium acetate voluntary wheel running mice; BL, baseline; PBS-UA, phosphate-buffered saline usual activity mice; PBS-VWR, phosphate-buffered saline voluntary wheel running mice; Pre, pre-intervention; post, post-intervention. *N* = 8 in each group.

### Predictive taxa using machine learning

**Fig 6:**
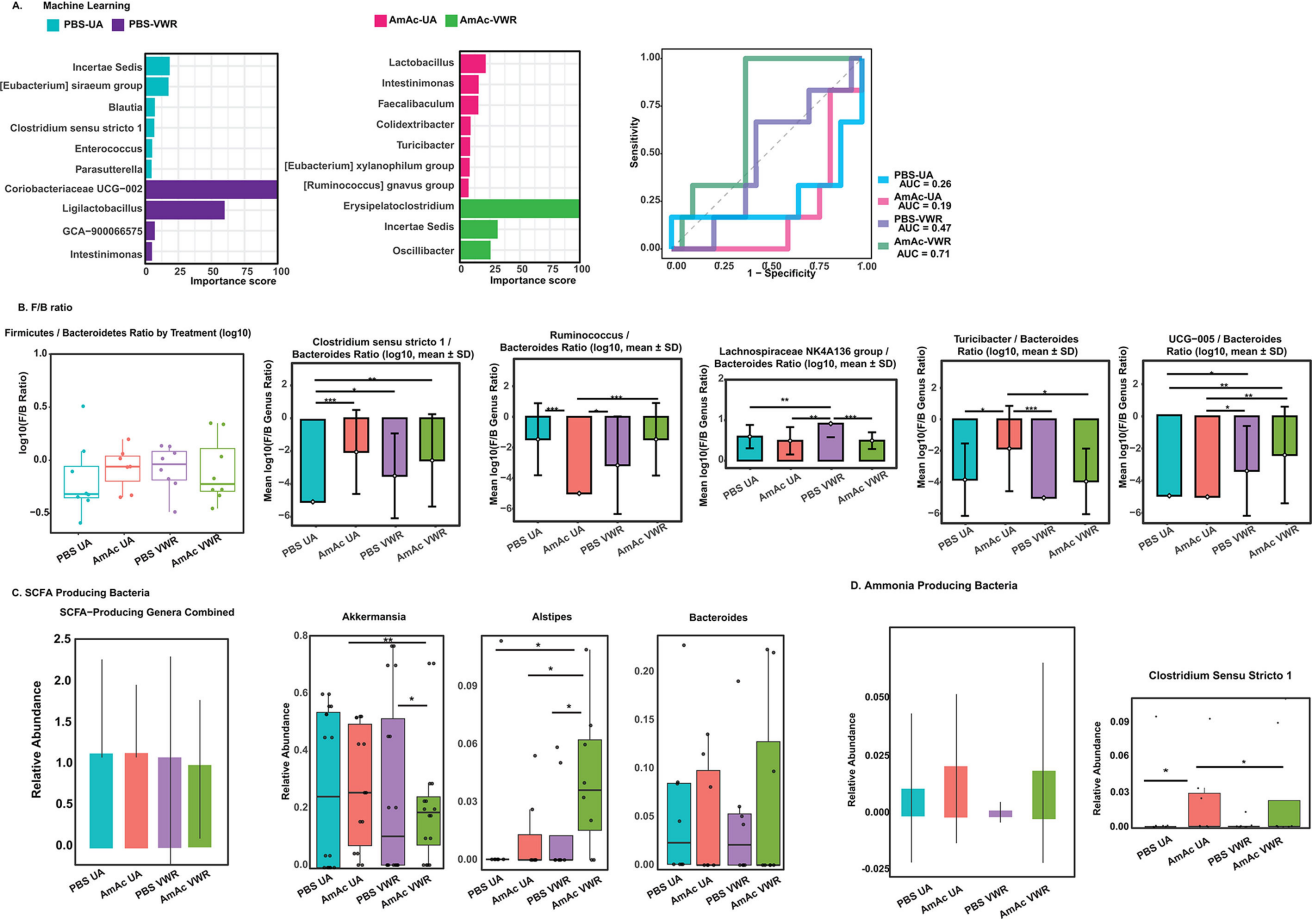
Machine learning approaches show changes in the gut microbiome during exercise. Male C57BL/6J mice aged 8–10 weeks were treated with either PBS or 2.5 mmol/kg/day AmAc for 42 days (treatment period). Two weeks after the osmotic pumps were installed, mice were randomized to UA or VWR for 4 weeks (intervention period). Stool samples were collected at three time points: baseline (prior to treatment or intervention), pre-intervention (2 weeks post-treatment), and post-intervention (4 weeks post-treatment). Post-intervention samples were sequenced and analyzed, and machine learning was performed. (**A**) Random Forest classifier using genus-level features to distinguish groups. (**B**) Log_10_-transformed Bacillota/Bacteroides ratios and genus-specific ratios across groups. (**C**) Relative abundance of SCFA-producing genera. (**D**) Abundance of ammonia-producing genera, including *Clostridium sensu stricto 1*. Data: mean ± SD. Statistical analyses: Random Forest model performance in panel A was evaluated using the area under the curve (AUC). For panel B, genus-level Bacillota/Bacteroides and genus/*Bacteroides* ratios were log_10_-transformed and analyzed using White’s non-parametric test with pooled variance and 1,000 bootstraps. For panels C and D, genus-level relative abundance of SCFA- and ammonia-producing taxa was arcsine square root-transformed and analyzed using White’s test with pooled variance and 1,000 bootstraps. *P*-values were corrected using the Benjamini-Hochberg method. *P < 0.05; **P < 0.01; ***P < 0.001. *n* = 8 mice per group.

Random forest analysis identified *Coriobacteriaceae* UCG-002 as a strong predictor of PBS-VWR and *Erysipelatoclostridium* as predictive of AmAc-VWR (Fig. 6A). Prediction accuracy was higher for AmAc-VWR (71%) than PBS-VWR (47%), indicating stronger classification during HAS. While overall *Bacillota*/*Bacteroidetes* ratios were unchanged (Fig. 6B and Fig. S8A), genus-level ratios differed across groups. *Clostridium sensu stricto 1*/Bacteroides and Turicibacter/Bacteroides were increased in AmAc-UA, whereas *Ruminococcus*/*Bacteroides* was reduced. In contrast, *Dubosiella*/*Bacteroides* ratios were preserved in the VWR groups. Consistent with these findings, the SCFA-associated taxon *Akkermansia* was reduced during HAS, while *Alistipes* was increased in AmAc-VWR (Fig. 6C). The ammonia-associated taxon *Clostridium sensu stricto 1* was highest in AmAc-UA mice (Fig. 6D). Together, these results demonstrate that exercise modifies predictive microbial features and partially reverses HAS-associated compositional and functional shifts.

### Predicted microbial functional pathways

To assess baseline functional stability, predicted microbial pathways were examined in PBS-UA control mice using PICRUSt2 MetaCyc analysis. No significant differences were observed between pre- and post-intervention samples (*P* > 0.05), indicating stable functional capacity over time (Fig. S9A). Although a small number of KO pathways reached statistical significance, these changes were limited and did not represent coordinated functional shifts (Fig. S9B). Consistent with these findings, Tax4Fun2 analysis also did not identify significant pathway differences between time points (Fig. S9). These analyses showed that microbial functional profiles remain stable in the absence of HAS or exercise.

In AmAc-UA mice, predicted functional pathway analysis revealed limited and inconsistent changes following intervention. PICRUSt2 MetaCyc and KO analyses did not identify significant pathway alterations between pre- and post-intervention samples (Fig. S10A and B), suggesting minimal coordinated functional shifts during HAS. In contrast, Tax4Fun2 analysis showed modest changes (*P* < 0.05) in pathways related to glycan degradation, branched-chain amino acid biosynthesis, and glycolysis (Fig. S10C). However, these changes were limited in magnitude and not broadly consistent across pathways. Together, these findings suggest that HAS alone induces modest alterations in predicted microbial metabolic function without major reorganization of functional capacity.

In PBS-VWR mice, predicted functional pathway analysis demonstrated exercise-induced remodeling of microbial metabolism. PICRUSt2 MetaCyc analysis identified alterations in pathways including histidine metabolism, and network analyses showed marked separation between pre- and post-intervention samples, indicating substantial functional restructuring (Fig. S11A and B). Tax4Fun2 analysis revealed enrichment of pathways related to bile acid metabolism and secondary metabolite biosynthesis following exercise (Fig. S11C). In AmAc-VWR mice, VWR induced pronounced functional changes as noted by PICRUSt2 MetaCyc analysis with significant increases (*P* < 0.01) in biosynthetic pathways, including serine and glycine biosynthesis, pyrimidine metabolism, and the S-adenosylmethionine cycle (Fig. S1,2A), with reductions in glycolysis and DNA repair pathways (Fig. S1,2B). Network analyses further showed clear separation between pre- and post-intervention samples, indicating substantial shifts in microbial functional capacity during HAS with VWR. In contrast, Tax4Fun2 analysis did not detect significant pathway changes in AmAc-VWR mice (Fig. S1,2C), suggesting differences in sensitivity between prediction methods. These observations show that exercise is associated with substantial changes in predicted microbial metabolic function, especially during HAS.

To evaluate consistency between functional prediction approaches, KEGG ortholog abundances derived from PICRUSt2 and Tax4Fun2 were compared across groups. Moderate positive correlations (*r* = 0.42–0.57) were observed between methods (Fig. S13), with the strongest agreement in AmAc-UA mice (*r* = 0.573). Lower but consistent correlations were observed in PBS-UA (*r* = 0.424), PBS-VWR (*r* = 0.511), and AmAc-VWR mice (*r* = 0.482). Our findings show that while PICRUSt2 and Tax4Fun2 identify broadly similar functional trends, PICRUSt2 shows greater sensitivity to functional shifts in our data. Although both approaches showed broadly similar directional trends, statistical concordance between methods was limited.

Comparisons of predicted microbial functional pathways across groups revealed distinct effects of HAS and VWR (Fig. S14A and B). During HAS (AmAc-UA vs PBS-UA), pathways related to central metabolism, transcriptional regulation, and methionine biosynthesis were enriched, whereas PBS controls showed enrichment of pathways associated with gene regulation and amino acid metabolism (*P* < 0.05). These functional profiles were modified by VWR. In AmAc-VWR vs PBS-VWR mice, HAS was associated with enrichment of pathways related to nutrient transport, intracellular processing, octane oxidation, and GABA degradation. In the AmAc group (AmAc-VWR vs AmAc-UA), VWR was associated with pathways involved in oxidative metabolism and nitrogen-related processes (*P* < 0.05). In PBS mice, VWR was associated with enrichment of pathways linked to fatty acid metabolism, tRNA charging, and broader metabolic and transcriptional processes compared to the UA group (*P* < 0.05).

Network analysis showed that the greatest distinction in functional organization occurred between PBS-UA and AmAc-UA mice, indicating a major effect of HAS, whereas VWR resulted in greater overlap among groups, suggesting partial convergence of functional network structure (Fig. S15). Tax4Fun2 analysis identified generally consistent directional trends but fewer statistically significant differences between groups. The most pronounced changes (*P* < 0.02) were observed in AmAc-VWR versus AmAc-UA comparisons, with alterations in pathways related to transport regulation, amino acid metabolism, and biosynthesis (Fig. S16), whereas minimal differences were observed in other comparisons. Direct comparison of PICRUSt2 and Tax4Fun2 outputs (Fig. S17) showed generally weak concordance across pathway-level analyses (*r* = −0.02 to 0.27), despite moderate directional agreement (53%–62%), indicating that both approaches identify similar trends but differ in sensitivity and pathway resolution.

Correlation analyses between microbial functional groups and host physiological parameters revealed distinct, context-dependent association patterns across treatment groups (Fig. S18 and Tables S1 and S2). In control mice (PBS-UA), SCFA-associated taxa correlated positively with muscle mass and negatively with ammonia-related measures, whereas urease-associated taxa showed opposing relationships. During HAS (AmAc-UA), SCFA-associated taxa were related to markers of metabolic stress, while urease-associated taxa correlated with increased ammonia and impaired muscle function. These relationships were altered, particularly in AmAc-VWR mice, where SCFA-associated taxa correlated with improved functional outcomes, such as grip strength, whereas urease-associated taxa correlated with exercise capacity and mitochondrial function. Together, these findings indicate that microbial functional groups exhibit distinct, context-dependent associations with host metabolism, supporting an association between microbiome composition and host adaptation under HAS.

## DISCUSSION

The present longitudinal study evaluates the impact of metabolic stress and voluntary exercise on the GMB. Destabilization of the GMB was characterized by reduced diversity, altered community structure, and depletion of beneficial taxa, including *Akkermansia* and *Eubacterium xylanophilum*. In contrast, VWR promoted restructuring of the microbial community, restoring *Akkermansia* and increasing the abundance of *Clostridium sensu stricto 1* and *Eubacterium* spp., thereby supporting a more stable and beneficial GMB during metabolic stress. These compositional changes were accompanied by enrichment of SCFA-producing bacteria. These data are consistent with prior literature on exercise-microbiome interactions and show that beneficial responses are maintained under metabolic stress.

At baseline, before any intervention, the GMB of all mice displayed similar alpha- and beta-diversity metrics, consistent with prior reports that C57BL/6J mice share a common core microbiome but demonstrate inter-individual variability (48). During HAS, microbial diversity decreased and community structure was altered, with enrichment of taxa such as *Eubacterium siraeum*, *Eubacterium ventriosum*, and *Akkermansia* suggesting ammonia as a potential driver of dysbiosis. Our data are consistent with altered GMB in decompensated cirrhosis, a condition associated with HAS (49, 50). However, the GMB signature of cirrhosis-related complications includes both shared and unique taxa; *Veillonella bacteria, Ruminococcus gnavus*, and *Streptococcus pneumoniae* are enriched in cirrhosis-related microbiota compared with the non-cirrhosis controls (49). *Bacteroides ovatus, Clostridium symbiosum, Emergencia timonensis, Fusobacterium varium*, and *Hungatella* were associated with complications in the cirrhosis group (49). These data suggest that multiple factors contribute to the GMB alterations in chronic diseases (51). Although increases in SCFA-associated taxa such as Eubacterium were observed, specific microbial patterns may also reflect disease severity rather than isolated metabolic effects (52).

Endurance exercise in both rodents and humans has been consistently associated with increased microbial diversity, enrichment of SCFA-producing bacteria, and stabilization of microbial networks (20, 21). In particular, VWR in rodents has been reported to increase the abundance of *Lachnospiraceae* and *Ruminococcaceae,* and expand the populations of *Lactobacillus* and *Bifidobacterium*. Consistent with these observations, our post-intervention findings show that VWR modifies HAS-driven microbial alterations. AmAc-UA mice had alpha-diversity and greatest intra-group variability, reflecting ecological instability during HAS. In contrast, AmAc-VWR mice displayed partial restoration of diversity and reduced variability with more consistent microbial networks. Notably, *Akkermansia* abundance was preserved in the AmAc-VWR group, consistent with exercise-induced enrichment of this genus in prior studies of healthy rodents and humans (23, 29). These observations show that VWR restores diversity and preserves longitudinal stability of the GMB during metabolic stress. The beneficial restructuring of GMB with VWR during HAS reflects the recognized benefits of continued exercise and is consistent with prior reports that “detraining” reverses the beneficial responses with exercise (53).

In our longitudinal analyses, GMB changes during VWR are consistent with prior reports (30). PBS-VWR mice showed modest restructuring of microbial co-occurrence patterns without major taxa-level shifts, consistent with endurance exercise exerting subtle but significant effects on microbial interactions (19, 24). In contrast, AmAc-UA mice exhibited progressive instability over time, including reductions in *Akkermansia* and *Lachnospiraceae*, suggesting disruption of both diversity and network stability during HAS. Random Forest classifier analyses further demonstrated that VWR drives distinct microbial signatures with taxa such as *Erysipelatoclostridium* and *Coriobacteriaceae* UCG-002 distinguishing AmAc-VWR and PBS-VWR mice, respectively. These findings support a context-dependent GMB response to exercise, varying between healthy and metabolically stressed states. Although overall *Bacillota*-to-*Bacteroidetes* ratios did not differ significantly, genus-level ratios (e.g., *Clostridium sensu stricto 1*/*Bacteroides*, *Ruminococcus/Bacteroides*) showed shifts in AmAc-UA mice, consistent with dysbiosis under HAS. Mechanistically, *Akkermansia* is associated with gut barrier function, anti-inflammatory activity, and improved metabolic health (54, 55), and its reduction during HAS may contribute to gut barrier disruption, endotoxemia, and systemic inflammation (11). Restoration of *Akkermansia* abundance with VWR may therefore contribute to the beneficial responses to exercise. Similarly, *Eubacterium xylanophilum* contributes to SCFA production and epithelial integrity (56, 57), and VWR-associated increases in these taxa may reflect adaptive responses to metabolic stress, including HAS. However, further functional metagenomic and metabolomic analyses are needed to confirm these interpretations. Our findings complement our prior work, demonstrating that VWR improves skeletal muscle oxidative capacity and protein homeostasis during HA (14), extending these observations to include microbiome-mediated adaptations.

Strengths of our study include the integration of longitudinal 16S rRNA sequencing with diversity, abundance, and network-based analyses, as well as machine-learning classifiers. The machine learning approach identified taxa such as *Eubacterium xylanophilum, Akkermansia* spp., and *Clostridium sensu stricto 1* as predictive features of treatment groups, suggesting that microbial signatures of exercise and hyperammonemia may represent candidate biomarkers for future validation. Given known differences between the mouse and human microbiomes (58), our findings require future validation in humanized microbiome models and human subjects. Mice were obtained from a single vendor (JAX) and maintained under standardized housing conditions, which minimizes but does not eliminate vendor- and facility-specific effects on microbial composition (59). Stool-based 16S rRNA sequencing cannot capture strain-level variation or fully characterize microbial metabolites such as SCFA, trimethylamine (TMA), and trimethylamine N-oxide (TMAO). To address this limitation, we incorporated predictive functional analyses using PICRUSt2 and Tax4Fun2, along with correlation analyses linking microbial features to host physiological parameters. Future studies incorporating metagenomics, metatranscriptomics, and metabolomics will enable more comprehensive evaluation of microbiome functional activity independent of compositional changes. The duration of VWR was relatively short, though our molecular and phenotypic responses to the protocol (14) suggest that it was sufficient to induce measurable changes in the GMB. Only male mice were included because HAS responses, including myostatin expression, are best characterized in males (60). However, given the recognized sex differences in microbiome responses to exercise (61), future studies should include both sexes. Notwithstanding these limitations, our findings provide a foundation for targeting the GMB to improve exercise responses in HAS and chronic diseases.

Our data also showed that predicted microbial functional capacity is differentially altered during HAS and to a greater extent by exercise. AmAc treatment alone induced only modest functional shifts, suggesting that hyperammonemia, while sufficient to alter microbial composition, has a more limited effect on predicted microbial metabolic function. In contrast, VWR resulted in greater functional remodeling. Exercise in PBS-treated mice resulted in substantial reorganization of predicted microbial pathways, with near-complete separation of functional network structure between pre- and post-intervention samples. During HAS, VWR induced distinct functional adaptations, including enrichment of biosynthetic pathways, alongside reductions in glycolysis and DNA repair pathways. These coordinated changes suggest altered microbial metabolic priorities in response to ammonia exposure and increased host metabolic demand. Importantly, functional grouping of taxa revealed divergence between SCFA-producing and urease-associated bacteria. SCFA-producing taxa were reduced under hyperammonemic conditions but were partially restored with exercise, consistent with the known role of SCFAs in maintaining gut barrier integrity and metabolic homeostasis (62, 63). In contrast, urease-associated taxa were increased in AmAc-treated mice and were not reversed by exercise, suggesting persistent enrichment of ammonia-generating functional potential. Correlation analyses further support these functional distinctions and show associations between microbial features and host physiological parameters, including muscle function and metabolic markers. SCFA-producing taxa were associated with markers of muscle function and metabolic activity, whereas urease-associated taxa were linked to ammonia-related measures and reduced functional outcomes. These associations link microbiome changes to systemic metabolic alterations during HAS and exercise. These findings suggest that predicted microbial functional capacity, rather than taxonomy alone, may play a critical role in mediating host responses during metabolic stress and exercise. However, causality between microbiome changes and host metabolic outcomes cannot be established from the present study.

PICRUSt2 and Tax4Fun2 are both predictive tools that infer microbial functional potential from 16S rRNA data, but they differ in their underlying approaches (Table S3). PICRUSt2 uses phylogenetic placement to estimate gene content based on evolutionary relationships, enabling functional prediction even for less well-characterized taxa (64), whereas Tax4Fun2 relies on direct mapping to reference genomes and is therefore more dependent on database coverage (65). As a result, PICRUSt2 tends to be more sensitive in detecting functional differences, while Tax4Fun2 provides a more conservative estimate of pathway changes with fewer statistically significant findings (66, 67). Previous comparative studies have reported only moderate concordance between these methods, with both approaches identifying overlapping core metabolic functions while also detecting distinct functional features unique to each method (66, 67). In addition, PICRUSt2 predictions show greater agreement with shotgun metagenomic profiles, although both Tax4Fun2 and PICRUSt2 are inference-based and may not fully identify all functional pathways (64). Despite these methodological differences, both Tax4Fun2 and PICRUSt2 identified similar overall functional trends in the present study. These predicted functional pathways were complemented by correlation analyses linking microbial functional groups with host phenotypes, providing independent support for biological relevance. These findings support our interpretations of the observed biological patterns and the complementary use of these tools for 16S-based functional inference (66, 67).

In conclusion, we show that HAS destabilizes the GMB and depletes key beneficial taxa, whereas VWR partially restores microbial stability (Graphical Abstract), enhances *Akkermansia*, and drives restructuring of microbial networks. Integration of machine-learning analyses identified taxa that distinguish exercise and disease states, suggesting potential relevance as biomarkers. Our findings have high translational relevance in cirrhosis and other chronic diseases with HAS, where gut dysbiosis includes reductions in *Akkermansia, Faecalibacterium*, and *Ruminococcaceae,* and an expansion of potentially pathogenic taxa (2, 3). These results also support a role for exercise in modulating host-GMB interactions during metabolic stress, with implications for managing hyperammonemia and related complications in chronic diseases. Future mechanistic studies should focus on how exercise-induced microbial changes influence ammonia metabolism, skeletal muscle function, and neurocognitive outcomes in models of chronic diseases, including cirrhosis.

## Data Availability

All sequencing data generated in this study have been deposited in the Zenodo repository and are publicly available at https://doi.org/10.5281/zenodo.17021627.

## SUPPLEMENTAL MATERIAL

Supplemental figure legends spectrum.03194-25-s0001.docx Legends for Figures S1 to S18.

Supplemental figures spectrum.03194-25-s0002.pdf Figures S1 to S18.

Graphical Abstract spectrum.03194-25-s0003.tif Graphical Abstract

Table S1 spectrum.03194-25-s0004.docx Correlations between microbial functional groups and host measures.

Table S2 spectrum.03194-25-s0005.xlsx Correlation matrix.

Table S3 spectrum.03194-25-s0006.docx Comparison of PICRUSt2 and Tax4Fun2 for prediction of microbial functional potential from 16S rRNA data.

ASM does not own the copyrights to Supplemental Material that may be linked to, or accessed through, an article. The authors have granted ASM a non-exclusive, world-wide license to publish the Supplemental Material files. Please contact the corresponding author directly for reuse.
