# Supplementary material for: Voluntary wheel running modulates murine gut microbiome during hyperammonemic stress
Source: Microbiol Spectr. 2026 Jul 15;14(8):e03194-25. doi: 10.1128/spectrum.03194-25 (PMC13435939; doi:10.1128/spectrum.03194-25)
Supplement: Supplementary file 1 [file spectrum.03194-25-s0002.pdf]

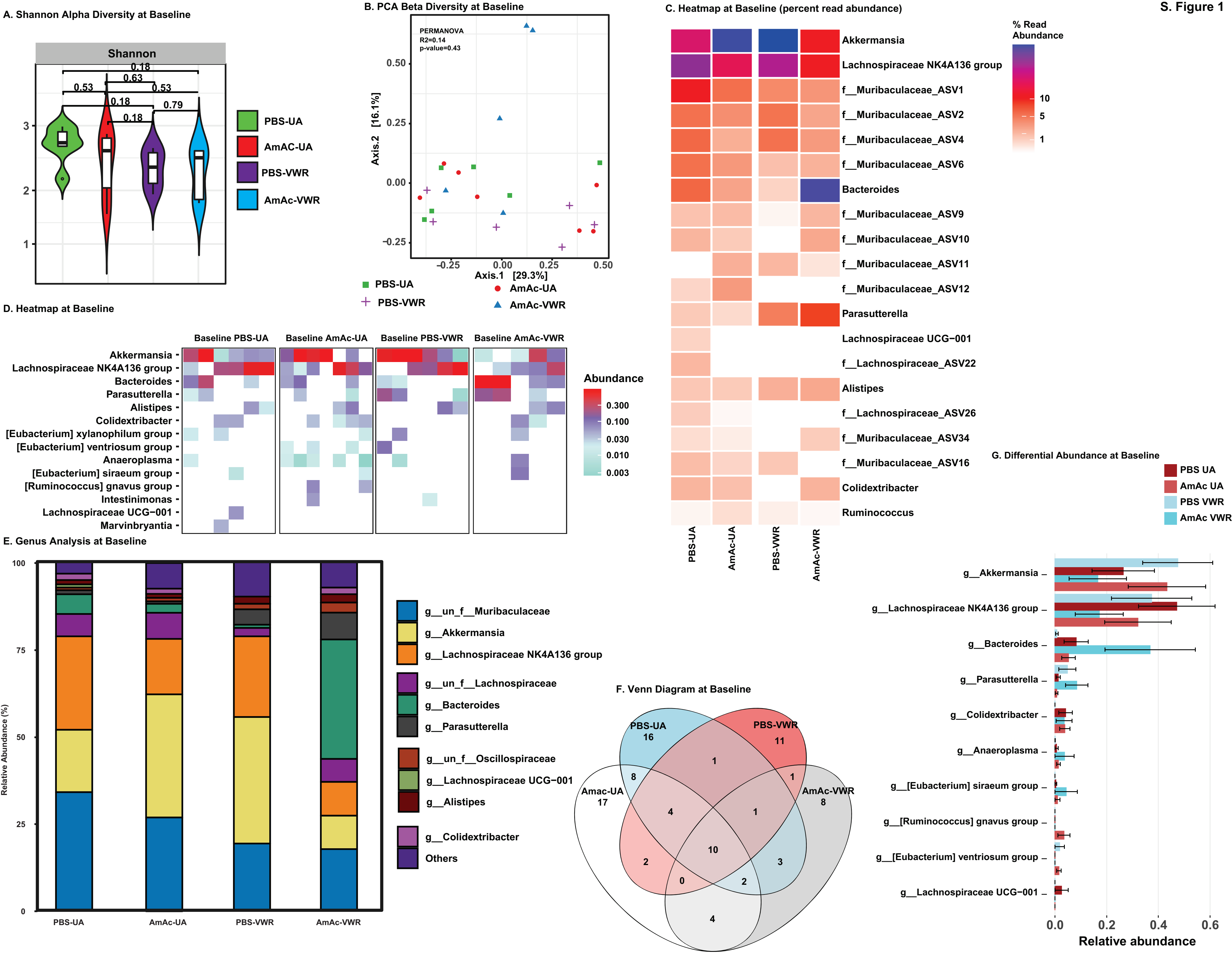
S. Figure 1
A. Shannon Alpha Diversity at Baseline
Shannon
PBS-UA
AmAC-UA
PBS-VWR
AmAc-VWR
B. PCA Beta Diversity at Baseline
PERMANOVA
R2=0.14
p-value=0.43
Axis.1 [29.3%]
Axis.2 [16.1%]
PBS-UA
PBS-VWR
AmAc-UA
AmAc-VWR
C. Heatmap at Baseline (percent read abundance)
% Read Abundance
Akkermansia
Lachnospiraceae NK4A136 group
f__Muribaculaceae_ASV1
f__Muribaculaceae_ASV2
f__Muribaculaceae_ASV4
f__Muribaculaceae_ASV6
Bacteroides
f__Muribaculaceae_ASV9
f__Muribaculaceae_ASV10
f__Muribaculaceae_ASV11
f__Muribaculaceae_ASV12
Parasutterella
Lachnospiraceae UCG−001
f__Lachnospiraceae_ASV22
Alistipes
f__Lachnospiraceae_ASV26
f__Muribaculaceae_ASV34
f__Muribaculaceae_ASV16
Colidextribacter
Ruminococcus
D. Heatmap at Baseline
Baseline PBS-UA
Baseline AmAc-UA
Baseline PBS-VWR
Baseline AmAc-VWR
Abundance
Akkermansia
Lachnospiraceae NK4A136 group
Bacteroides
Parasutterella
Alistipes
Colidextribacter
[Eubacterium] xylanophilum group
[Eubacterium] ventriosum group
Anaeroplasma
[Eubacterium] siraeum group
[Ruminococcus] gnavus group
Intestinimonas
Lachnospiraceae UCG−001
Marvinbryantia
E. Genus Analysis at Baseline
Relative Abundance (%)
g__un_f__Muribaculaceae
g__Akkermansia
g__Lachnospiraceae NK4A136 group
g__un_f__Lachnospiraceae
g__Bacteroides
g__Parasutterella
g__un_f__Oscillospiraceae
g__Lachnospiraceae UCG−001
g__Alistipes
g__Colidextribacter
Others
F. Venn Diagram at Baseline
PBS-UA 16
PBS-VWR 11
Amac-UA 17
AmAc-VWR 8
G. Differential Abundance at Baseline
PBS UA
AmAc UA
PBS VWR
AmAc VWR
g__Akkermansia
g__Lachnospiraceae NK4A136 group
g__Bacteroides
g__Parasutterella
g__Colidextribacter
g__Anaeroplasma
g__[Eubacterium] siraeum group
g__[Ruminococcus] gnavus group
g__[Eubacterium] ventriosum group
g__Lachnospiraceae UCG−001
Relative abundance

S. Figure 2

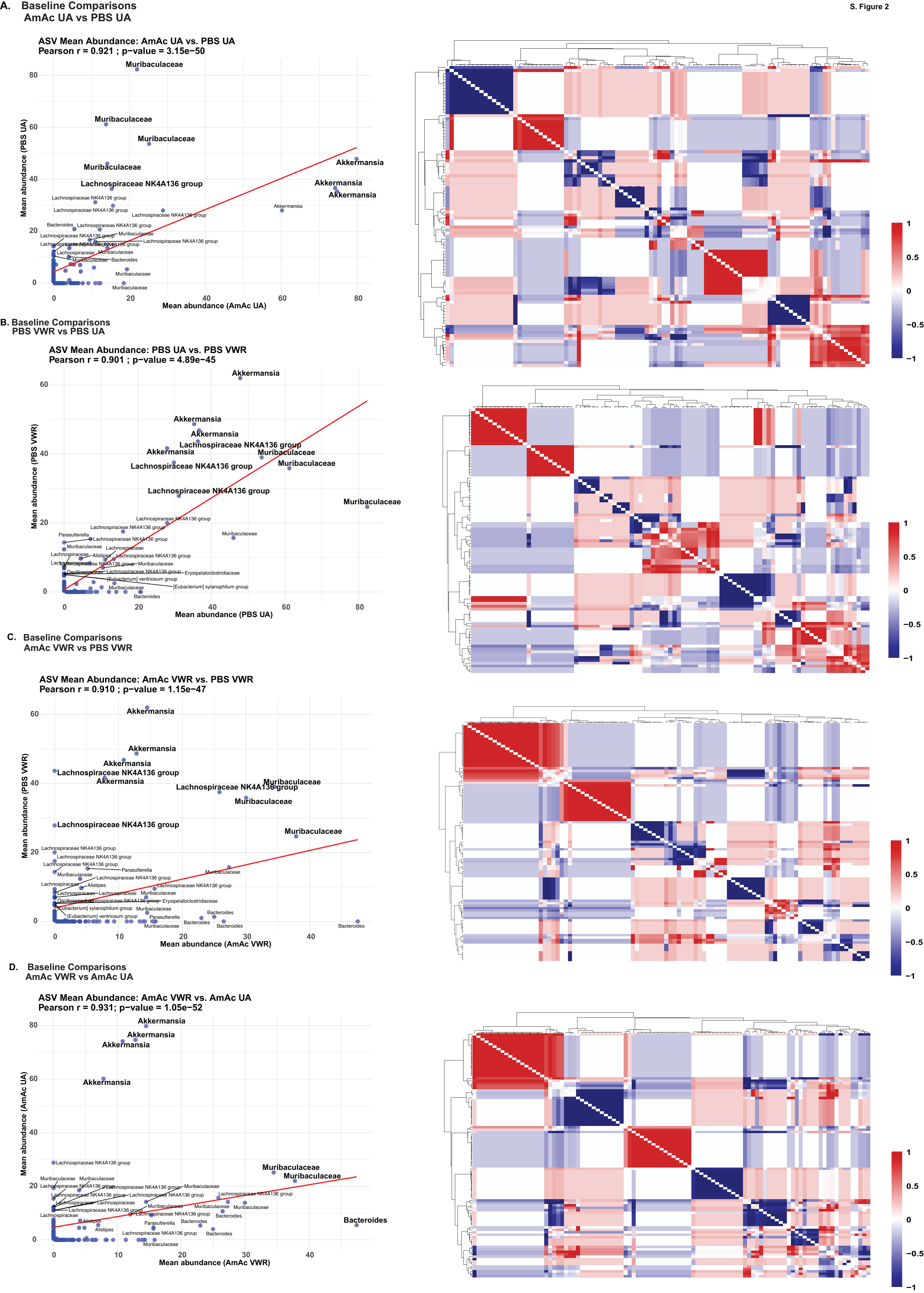
A. Baseline Comparisons
AmAc UA vs PBS UA
ASV Mean Abundance: AmAc UA vs. PBS UA
Pearson r = 0.921 ; p-value = 3.15e−50
Mean abundance (PBS UA)
Mean abundance (AmAc UA)
Muribaculaceae
Lachnospiraceae NK4A136 group
Akkermansia
B. Baseline Comparisons
PBS VWR vs PBS UA
ASV Mean Abundance: PBS UA vs. PBS VWR
Pearson r = 0.901 ; p-value = 4.89e−45
Mean abundance (PBS VWR)
Mean abundance (PBS UA)
Akkermansia
Lachnospiraceae NK4A136 group
Muribaculaceae
C. Baseline Comparisons
AmAc VWR vs PBS VWR
ASV Mean Abundance: AmAc VWR vs. PBS VWR
Pearson r = 0.910 ; p-value = 1.15e−47
Mean abundance (PBS VWR)
Mean abundance (AmAc VWR)
Akkermansia
Lachnospiraceae NK4A136 group
Muribaculaceae
D. Baseline Comparisons
AmAc VWR vs AmAc UA
ASV Mean Abundance: AmAc VWR vs. AmAc UA
Pearson r = 0.931; p-value = 1.05e−52
Mean abundance (AmAc UA)
Mean abundance (AmAc VWR)
Akkermansia
Muribaculaceae
Bacteroides

A.

S. Figure 3

Correlation Difference (AmAc UA minus PBS UA) Baseline

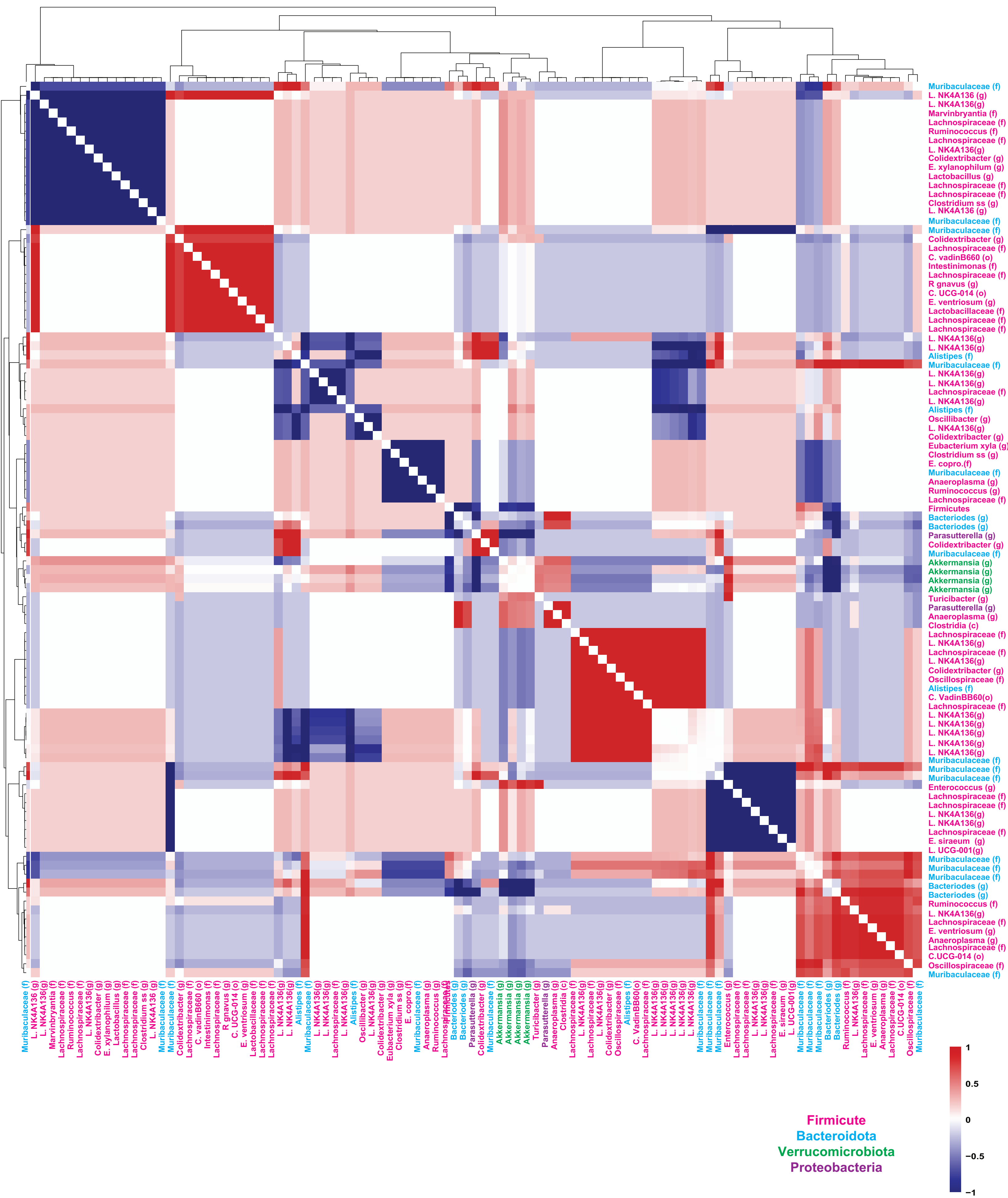

B.

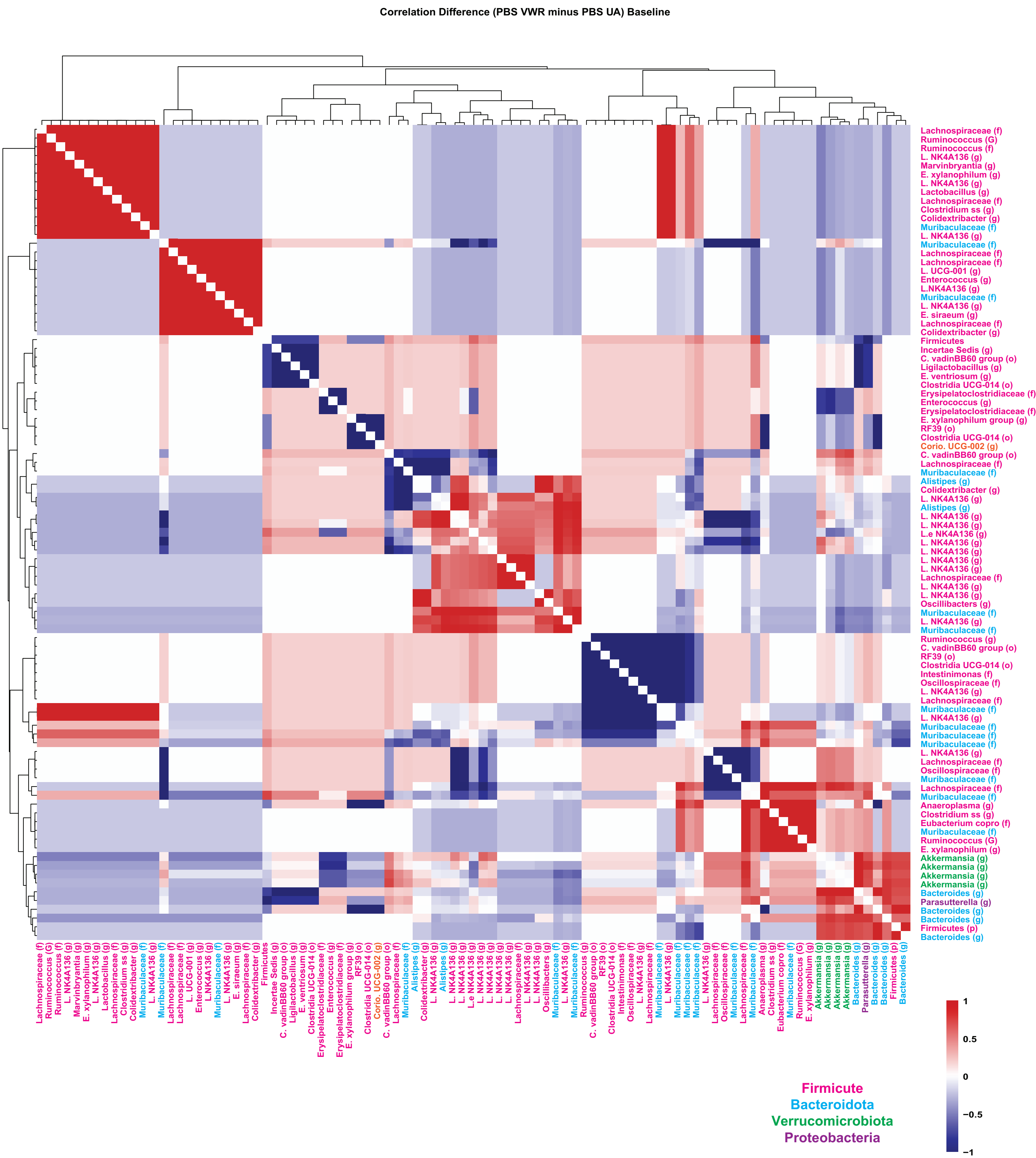

Correlation Difference (PBS VWR minus PBS UA) Baseline
Lachnospiraceae (f)
Ruminococcus (G)
Ruminococcus (f)
L. NK4A136 (g)
Marvinbryantia (g)
E. xylanophilum (g)
L. NK4A136 (g)
Lactobacillus (g)
Lachnospiraceae (f)
Clostridium ss (g)
Colidextribacter (g)
Muribaculaceae (f)
L. NK4A136 (g)
Muribaculaceae (f)
Lachnospiraceae (f)
Lachnospiraceae (f)
L. UCG-001 (g)
Enterococcus (g)
L.NK4A136 (g)
Muribaculaceae (f)
L. NK4A136 (g)
E. siraeum (g)
Lachnospiraceae (f)
Colidextribacter (g)
Firmicutes
Incertae Sedis (g)
C. vadinBB60 group (o)
Ligilactobacillus (g)
E. ventriosum (g)
Clostridia UCG-014 (o)
Erysipelatoclostridiaceae (f)
Enterococcus (g)
Erysipelatoclostridiaceae (f)
E. xylanophilum group (g)
RF39 (o)
Clostridia UCG-014 (o)
Corio. UCG-002 (g)
C. vadinBB60 group (o)
Lachnospiraceae (f)
Muribaculaceae (f)
Alistipes (g)
Colidextribacter (g)
L. NK4A136 (g)
Alistipes (g)
L. NK4A136 (g)
L. NK4A136 (g)
L.e NK4A136 (g)
L. NK4A136 (g)
L. NK4A136 (g)
L. NK4A136 (g)
L. NK4A136 (g)
Lachnospiraceae (f)
L. NK4A136 (g)
L. NK4A136 (g)
Oscillibacters (g)
Muribaculaceae (f)
L. NK4A136 (g)
Muribaculaceae (f)
Ruminococcus (g)
C. vadinBB60 group (o)
RF39 (o)
Clostridia UCG-014 (o)
Intestinimonas (f)
Oscillospiraceae (f)
L. NK4A136 (g)
Lachnospiraceae (f)
Muribaculaceae (f)
L. NK4A136 (g)
Muribaculaceae (f)
Muribaculaceae (f)
Muribaculaceae (f)
L. NK4A136 (g)
Lachnospiraceae (f)
Oscillospiraceae (f)
Muribaculaceae (f)
Lachnospiraceae (f)
Muribaculaceae (f)
Anaeroplasma (g)
Clostridium ss (g)
Eubacterium copro (f)
Muribaculaceae (f)
Ruminococcus (G)
E. xylanophilum (g)
Akkermansia (g)
Akkermansia (g)
Akkermansia (g)
Akkermansia (g)
Bacteroides (g)
Parasutterella (g)
Bacteroides (g)
Bacteroides (g)
Firmicutes (p)
Bacteroides (g)
1
0.5
0
−0.5
−1
Firmicute
Bacteroidota
Verrucomicrobiota
Proteobacteria

c.

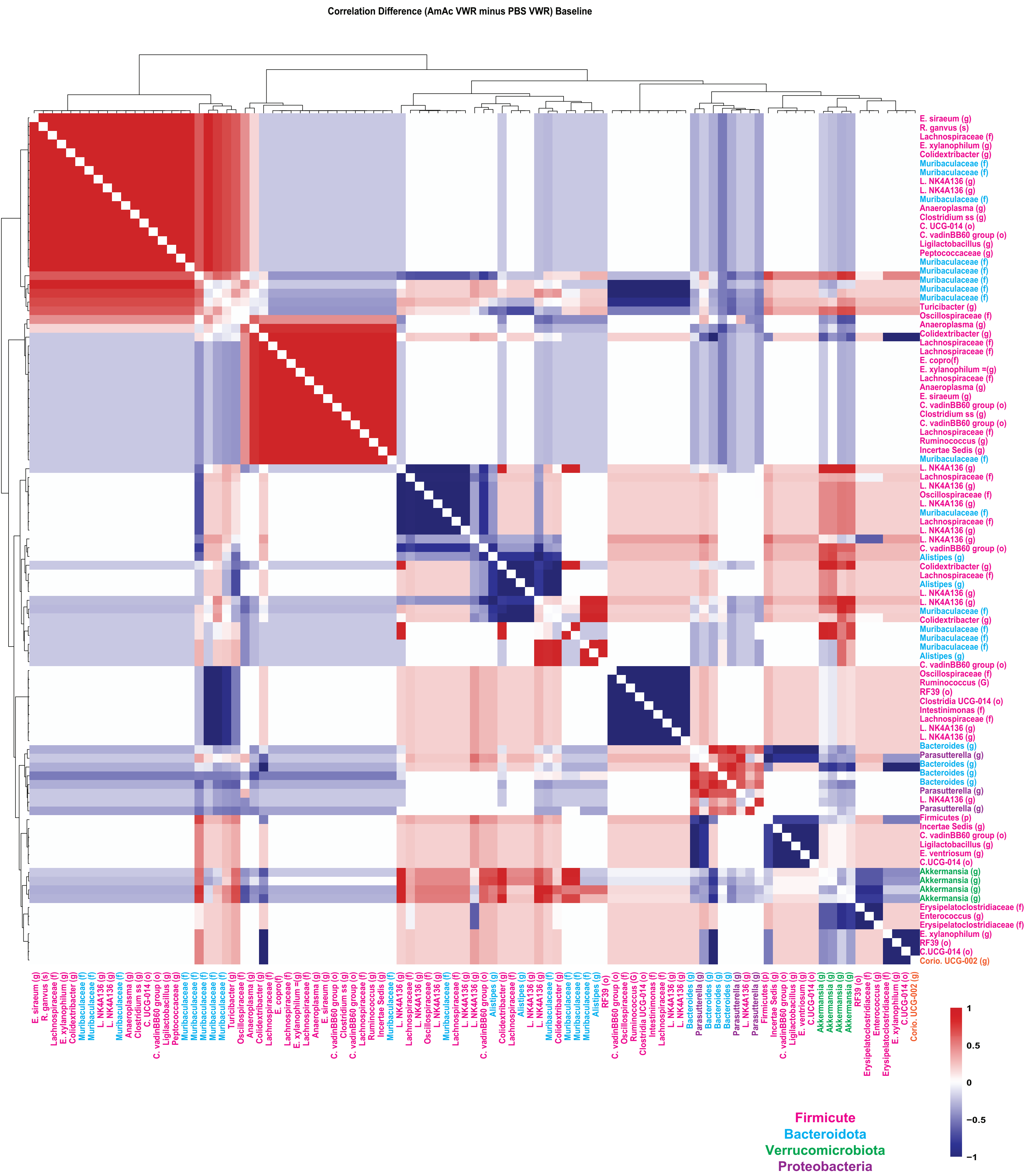
Correlation Difference (AmAc VWR minus PBS VWR) Baseline
E. siraeum (g)
R. ganvus (s)
Lachnospiraceae (f)
E. xylanophilum (g)
Colidextribacter (g)
Muribaculaceae (f)
Muribaculaceae (f)
L. NK4A136 (g)
L. NK4A136 (g)
Muribaculaceae (f)
Anaeroplasma (g)
Clostridium ss (g)
C. UCG-014 (o)
C. vadinBB60 group (o)
Ligilactobacillus (g)
Peptococcaceae (g)
Muribaculaceae (f)
Muribaculaceae (f)
Muribaculaceae (f)
Muribaculaceae (f)
Muribaculaceae (f)
Turicibacter (g)
Oscillospiraceae (f)
Anaeroplasma (g)
Colidextribacter (g)
Lachnospiraceae (f)
Lachnospiraceae (f)
E. copro(f)
E. xylanophilum =(g)
Lachnospiraceae (f)
Anaeroplasma (g)
E. siraeum (g)
C. vadinBB60 group (o)
Clostridium ss (g)
C. vadinBB60 group (o)
Lachnospiraceae (f)
Ruminococcus (g)
Incertae Sedis (g)
Muribaculaceae (f)
L. NK4A136 (g)
Lachnospiraceae (f)
L. NK4A136 (g)
Oscillospiraceae (f)
L. NK4A136 (g)
Muribaculaceae (f)
Lachnospiraceae (f)
L. NK4A136 (g)
L. NK4A136 (g)
C. vadinBB60 group (o)
Alistipes (g)
Colidextribacter (g)
Lachnospiraceae (f)
Alistipes (g)
L. NK4A136 (g)
L. NK4A136 (g)
Muribaculaceae (f)
Colidextribacter (g)
Muribaculaceae (f)
Muribaculaceae (f)
Muribaculaceae (f)
Alistipes (g)
C. vadinBB60 group (o)
Oscillospiraceae (f)
Ruminococcus (G)
RF39 (o)
Clostridia UCG-014 (o)
Intestinimonas (f)
Lachnospiraceae (f)
L. NK4A136 (g)
L. NK4A136 (g)
Bacteroides (g)
Parasutterella (g)
Bacteroides (g)
Bacteroides (g)
Bacteroides (g)
Parasutterella (g)
L. NK4A136 (g)
Parasutterella (g)
Firmicutes (p)
Incertae Sedis (g)
C. vadinBB60 group (o)
Ligilactobacillus (g)
E. ventriosum (g)
C.UCG-014 (o)
Akkermansia (g)
Akkermansia (g)
Akkermansia (g)
Akkermansia (g)
Erysipelatoclostridiaceae (f)
Enterococcus (g)
Erysipelatoclostridiaceae (f)
E. xylanophilum (g)
RF39 (o)
C.UCG-014 (o)
Corio. UCG-002 (g)
1
0.5
0
−0.5
−1
Firmicute
Bacteroidota
Verrucomicrobiota
Proteobacteria

D.

S. Figure 3

Correlation Difference (AmAc VWR minus AmAc UA) Baseline

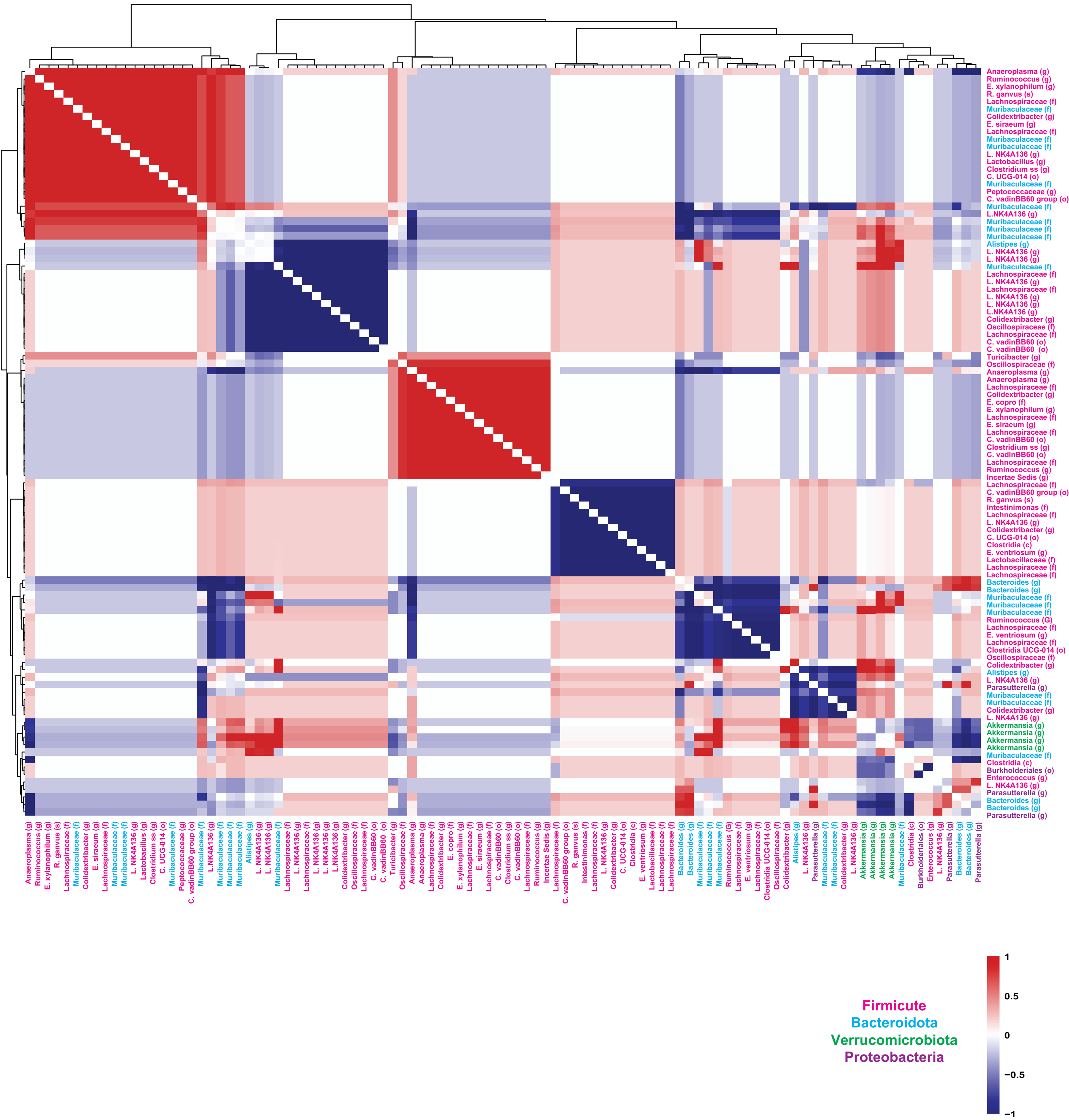

S. Figure 4

A. PBS UA Correlation Difference (Baseline minus Pre)

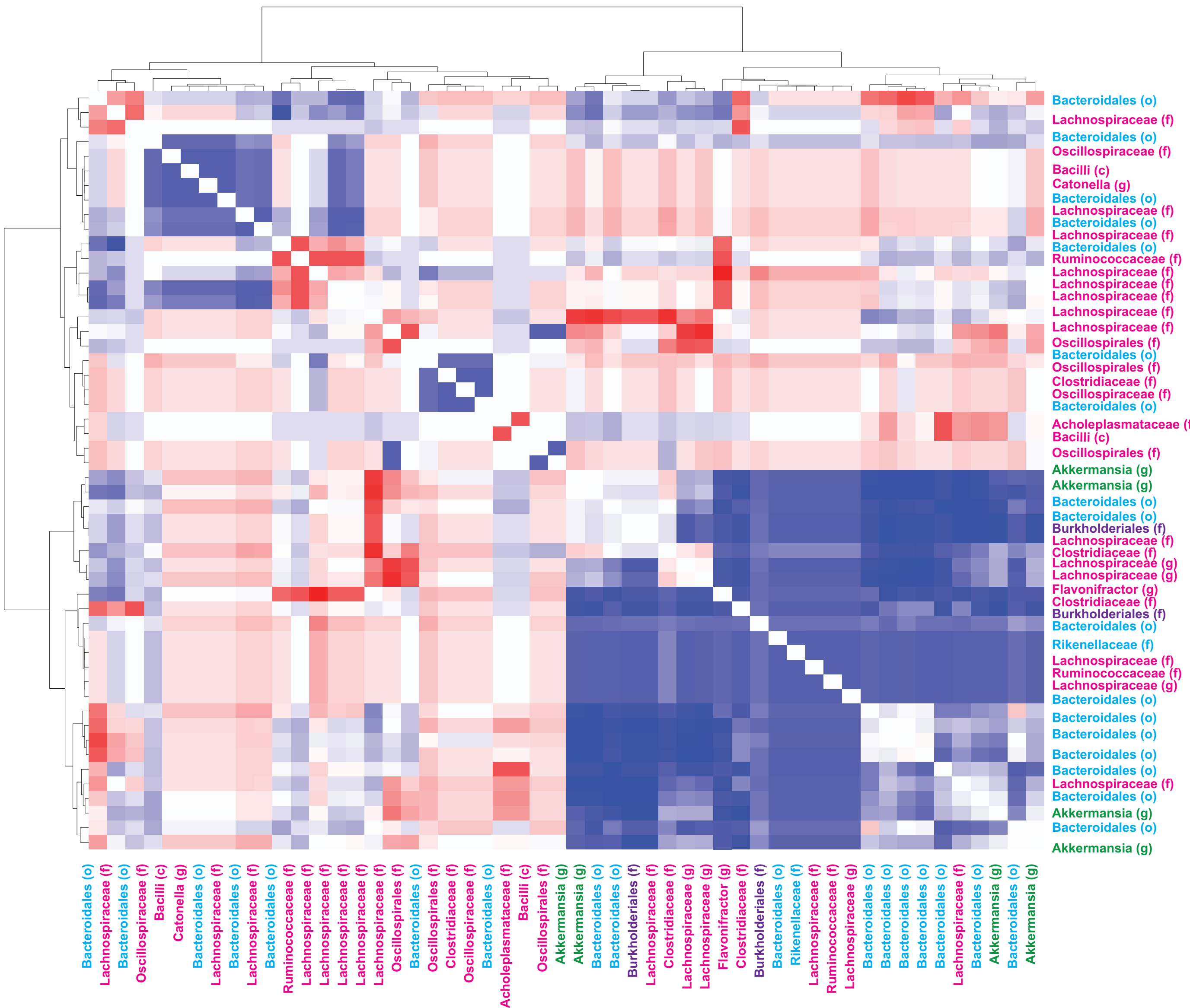

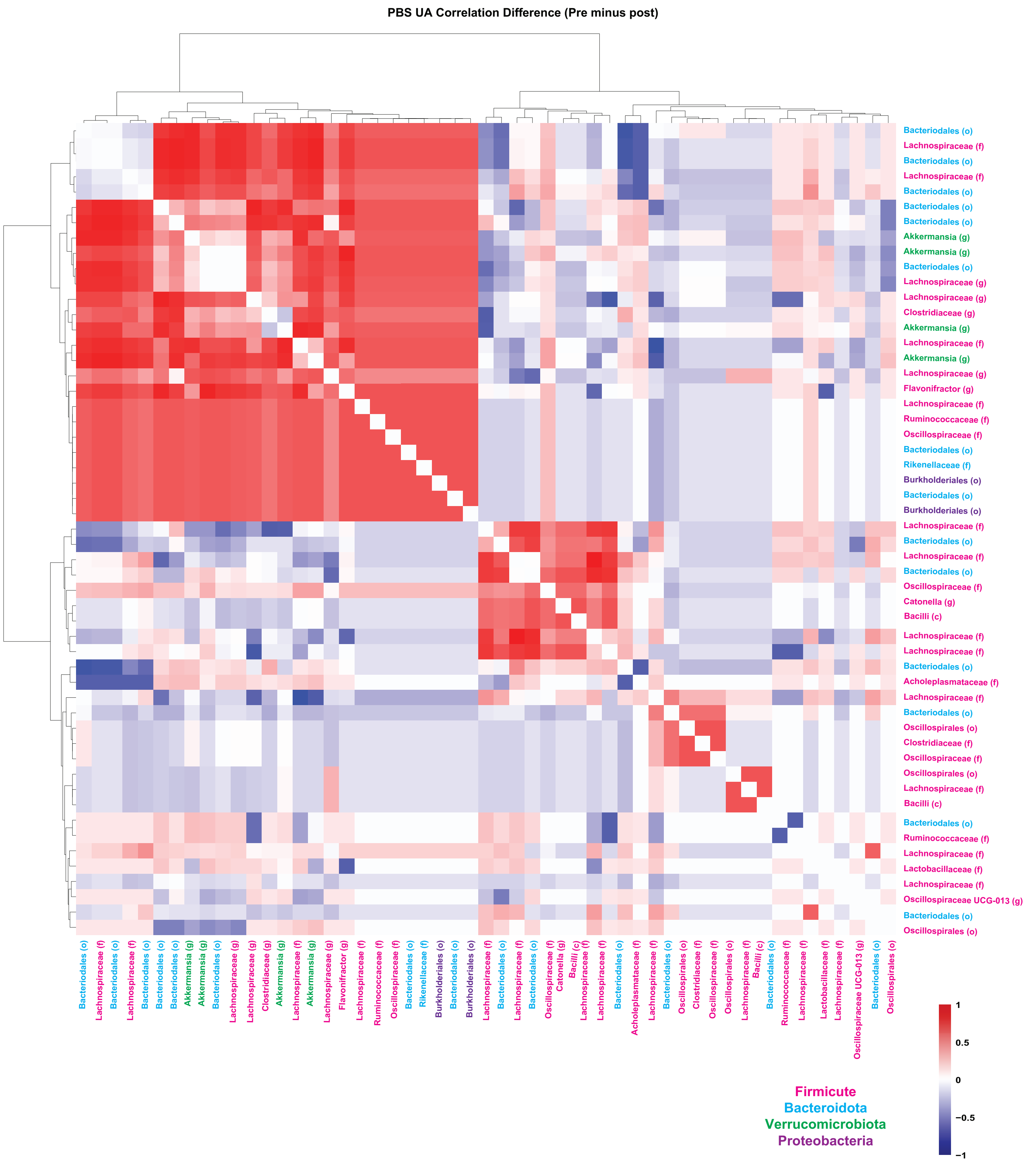
PBS UA Correlation Difference (Pre minus post)
Bacteriodales (o)
Lachnospiraceae (f)
Bacteriodales (o)
Lachnospiraceae (f)
Bacteriodales (o)
Bacteriodales (o)
Bacteriodales (o)
Akkermansia (g)
Akkermansia (g)
Bacteriodales (o)
Lachnospiraceae (g)
Lachnospiraceae (g)
Clostridiaceae (g)
Akkermansia (g)
Lachnospiraceae (f)
Akkermansia (g)
Lachnospiraceae (g)
Flavonifractor (g)
Lachnospiraceae (f)
Ruminococcaceae (f)
Oscillospiraceae (f)
Bacteriodales (o)
Rikenellaceae (f)
Burkholderiales (o)
Bacteriodales (o)
Burkholderiales (o)
Lachnospiraceae (f)
Bacteriodales (o)
Lachnospiraceae (f)
Bacteriodales (o)
Oscillospiraceae (f)
Catonella (g)
Bacilli (c)
Lachnospiraceae (f)
Lachnospiraceae (f)
Bacteriodales (o)
Acholeplasmataceae (f)
Lachnospiraceae (f)
Bacteriodales (o)
Oscillospirales (o)
Clostridiaceae (f)
Oscillospiraceae (f)
Oscillospirales (o)
Lachnospiraceae (f)
Bacilli (c)
Bacteriodales (o)
Ruminococcaceae (f)
Lachnospiraceae (f)
Lactobacillaceae (f)
Lachnospiraceae (f)
Oscillospiraceae UCG-013 (g)
Bacteriodales (o)
Oscillospirales (o)
1
0.5
0
−0.5
−1
Firmicute
Bacteroidota
Verrucomicrobiota
Proteobacteria

S. Figure 4

C.

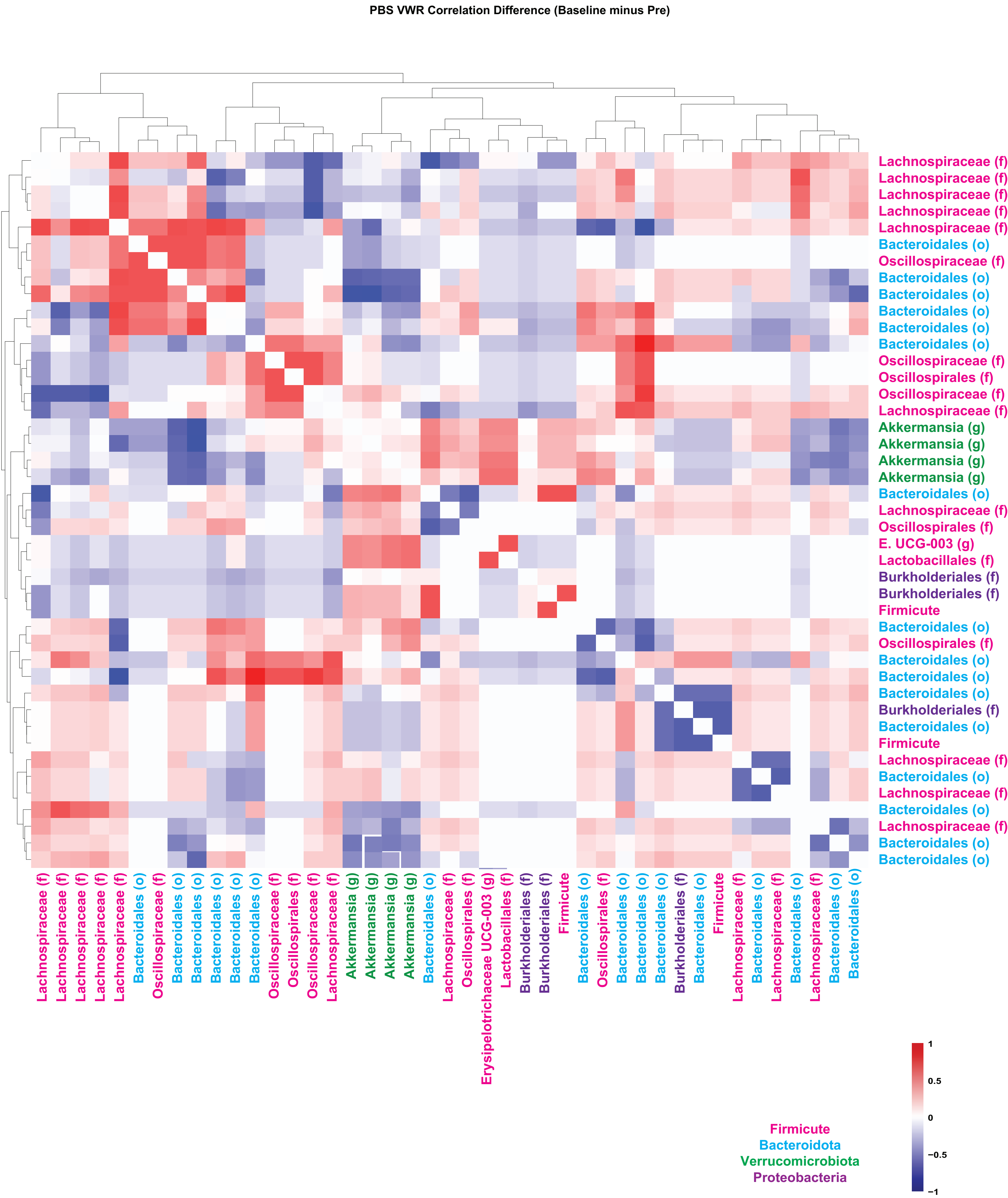
PBS VWR Correlation Difference (Baseline minus Pre)
Lachnospiraceae (f)
Lachnospiraceae (f)
Lachnospiraceae (f)
Lachnospiraceae (f)
Lachnospiraceae (f)
Bacteroidales (o)
Oscillospiraceae (f)
Bacteroidales (o)
Bacteroidales (o)
Bacteroidales (o)
Bacteroidales (o)
Bacteroidales (o)
Oscillospiraceae (f)
Oscillospirales (f)
Oscillospiraceae (f)
Lachnospiraceae (f)
Akkermansia (g)
Akkermansia (g)
Akkermansia (g)
Akkermansia (g)
Bacteroidales (o)
Lachnospiraceae (f)
Oscillospirales (f)
E. UCG-003 (g)
Lactobacillales (f)
Burkholderiales (f)
Burkholderiales (f)
Firmicute
Bacteroidales (o)
Oscillospirales (f)
Bacteroidales (o)
Bacteroidales (o)
Bacteroidales (o)
Burkholderiales (f)
Bacteroidales (o)
Firmicute
Lachnospiraceae (f)
Bacteroidales (o)
Lachnospiraceae (f)
Bacteroidales (o)
Lachnospiraceae (f)
Bacteroidales (o)
Bacteroidales (o)
Erysipelotrichaceae UCG-003 (g)
1
0.5
0
−0.5
−1
Firmicute
Bacteroidota
Verrucomicrobiota
Proteobacteria

D.

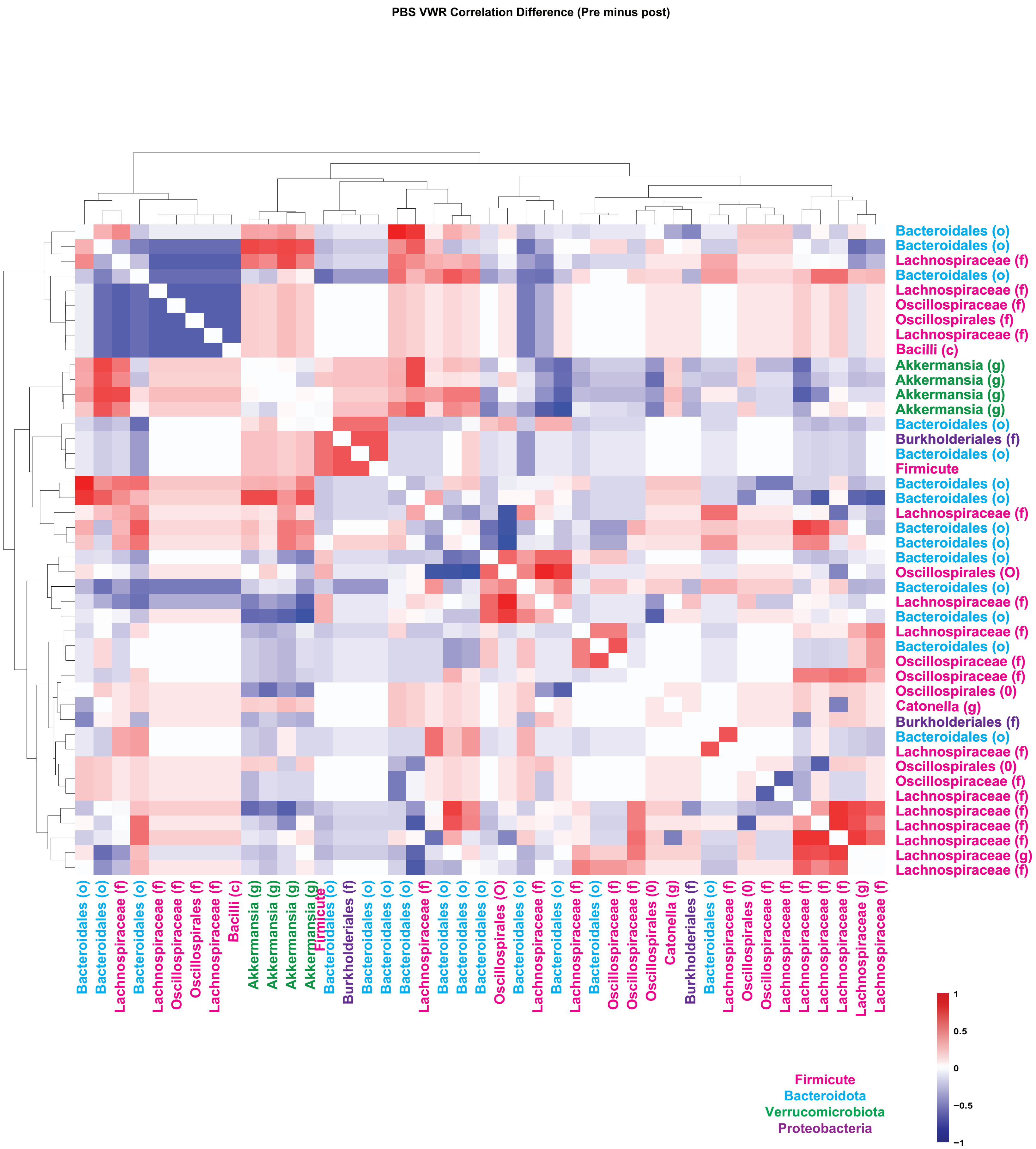
PBS VWR Correlation Difference (Pre minus post)
Bacteroidales (o)
Bacteroidales (o)
Lachnospiraceae (f)
Bacteroidales (o)
Lachnospiraceae (f)
Oscillospiraceae (f)
Oscillospirales (f)
Lachnospiraceae (f)
Bacilli (c)
Akkermansia (g)
Akkermansia (g)
Akkermansia (g)
Akkermansia (g)
Bacteroidales (o)
Burkholderiales (f)
Bacteroidales (o)
Firmicute
Bacteroidales (o)
Bacteroidales (o)
Lachnospiraceae (f)
Bacteroidales (o)
Bacteroidales (o)
Bacteroidales (o)
Oscillospirales (O)
Bacteroidales (o)
Lachnospiraceae (f)
Bacteroidales (o)
Lachnospiraceae (f)
Bacteroidales (o)
Oscillospiraceae (f)
Oscillospiraceae (f)
Oscillospirales (0)
Catonella (g)
Burkholderiales (f)
Bacteroidales (o)
Lachnospiraceae (f)
Oscillospirales (0)
Oscillospiraceae (f)
Lachnospiraceae (f)
Lachnospiraceae (f)
Lachnospiraceae (f)
Lachnospiraceae (f)
Lachnospiraceae (g)
Lachnospiraceae (f)
1
0.5
0
−0.5
−1
Firmicute
Bacteroidota
Verrucomicrobiota
Proteobacteria

E.

S. Figure 4

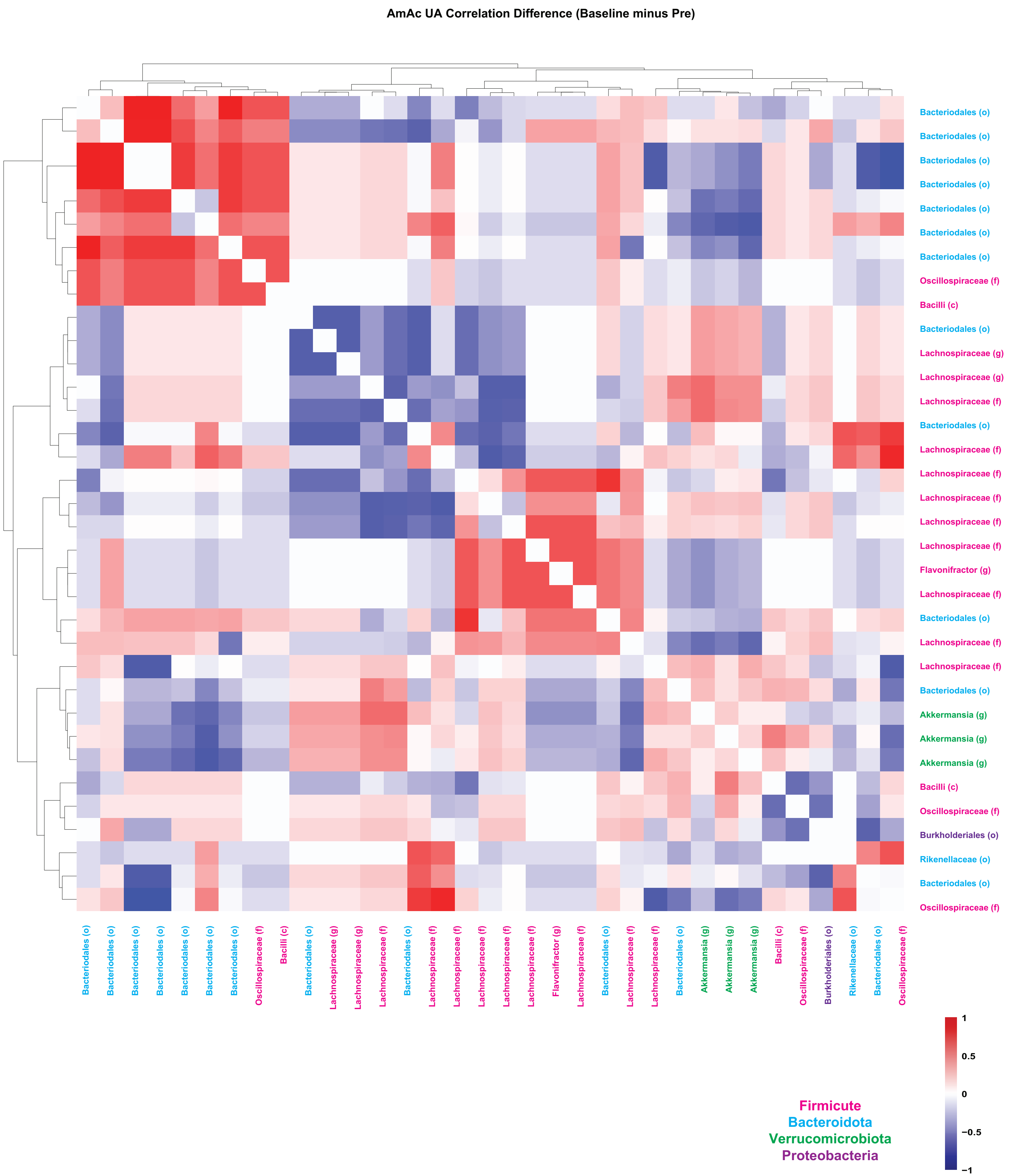
AmAc UA Correlation Difference (Baseline minus Pre)
Bacteriodales (o)
Bacteriodales (o)
Bacteriodales (o)
Bacteriodales (o)
Bacteriodales (o)
Bacteriodales (o)
Bacteriodales (o)
Oscillospiraceae (f)
Bacilli (c)
Bacteriodales (o)
Lachnospiraceae (g)
Lachnospiraceae (g)
Lachnospiraceae (f)
Bacteriodales (o)
Lachnospiraceae (f)
Lachnospiraceae (f)
Lachnospiraceae (f)
Lachnospiraceae (f)
Lachnospiraceae (f)
Flavonifractor (g)
Lachnospiraceae (f)
Bacteriodales (o)
Lachnospiraceae (f)
Lachnospiraceae (f)
Bacteriodales (o)
Akkermansia (g)
Akkermansia (g)
Akkermansia (g)
Bacilli (c)
Oscillospiraceae (f)
Burkholderiales (o)
Rikenellaceae (o)
Bacteriodales (o)
Oscillospiraceae (f)
1
0.5
0
−0.5
−1
Firmicute
Bacteroidota
Verrucomicrobiota
Proteobacteria

AmAc UA Correlation Difference (Pre minus post)

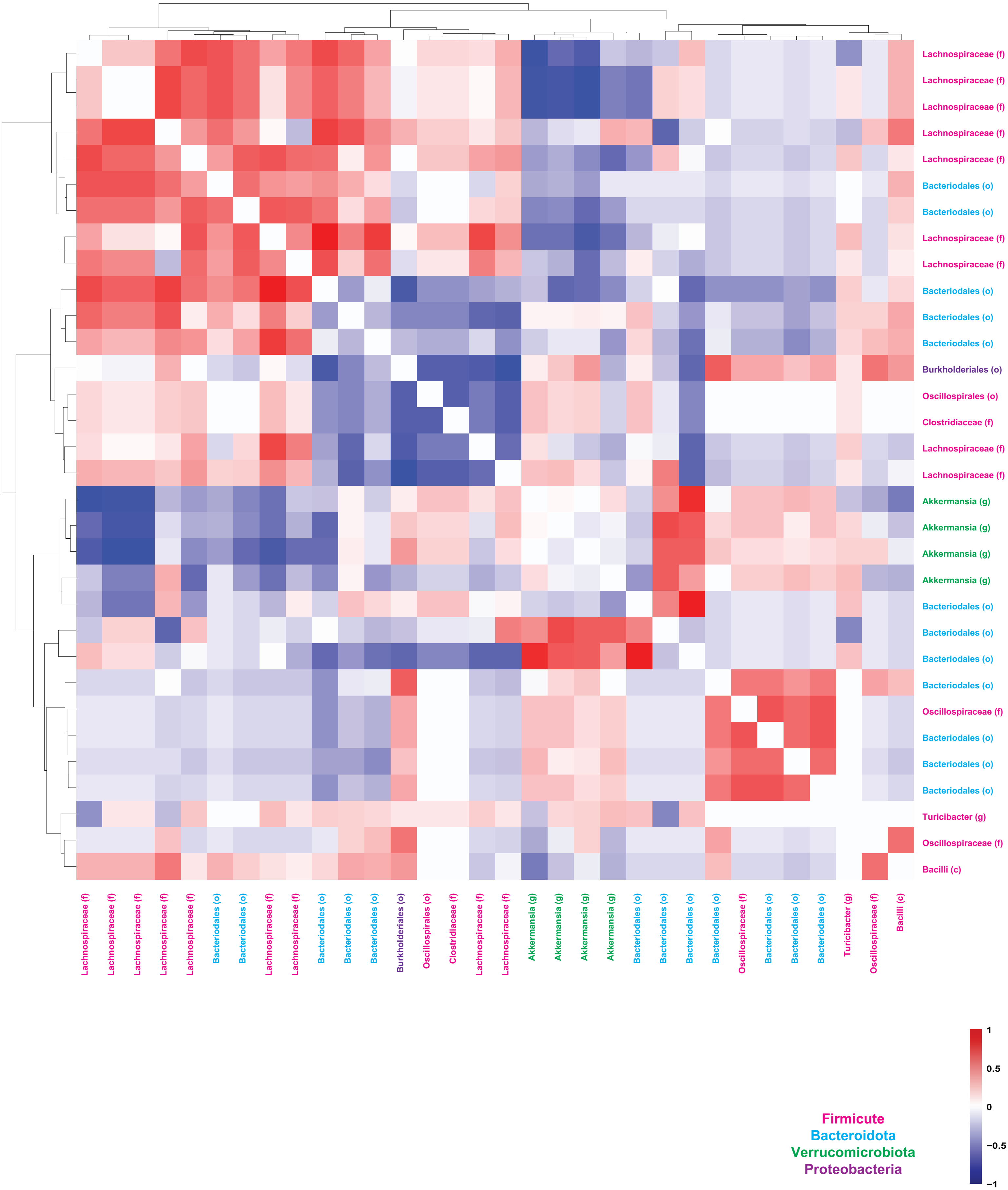

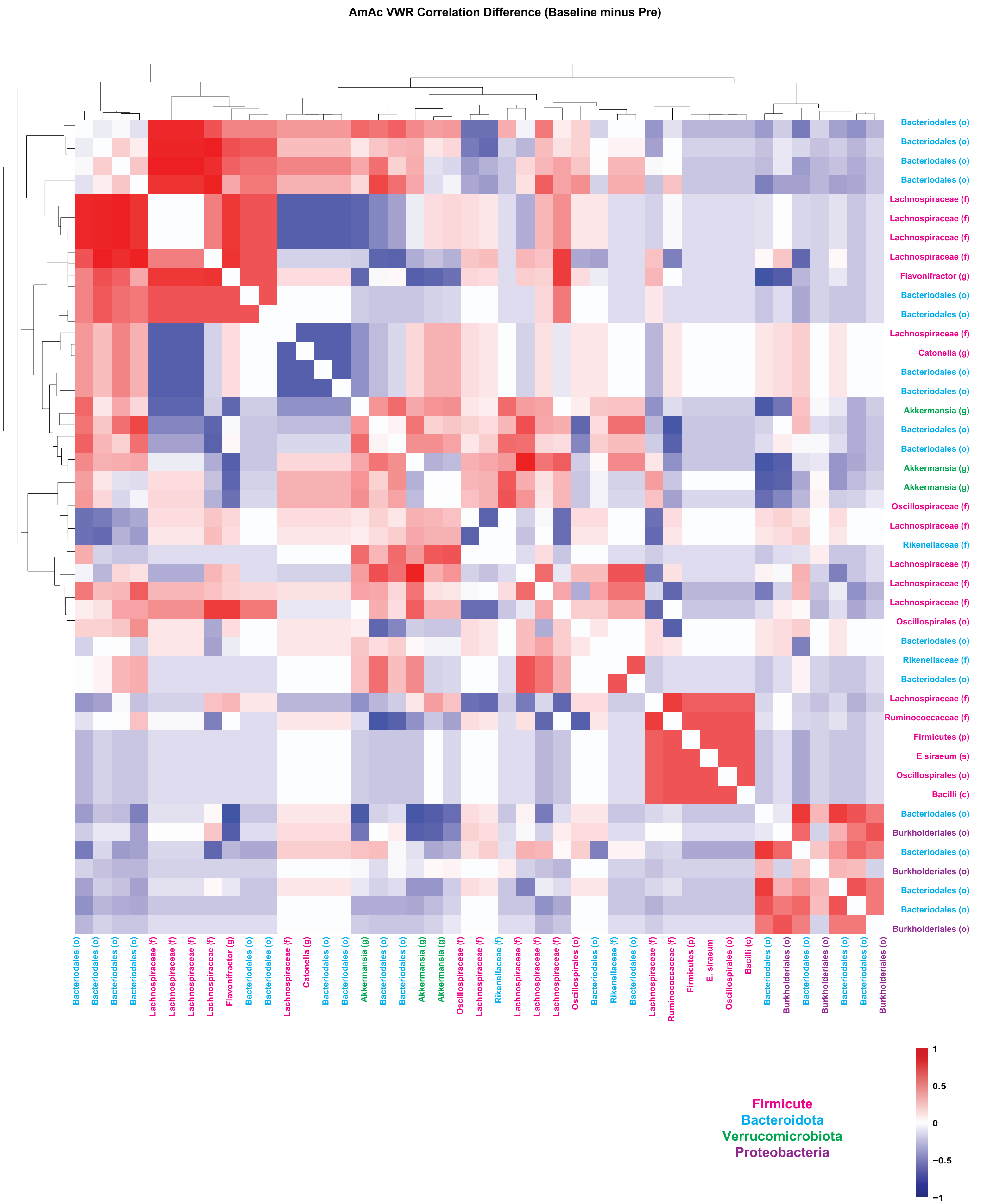
AmAc VWR Correlation Difference (Baseline minus Pre)
Bacteriodales (o)
Bacteriodales (o)
Bacteriodales (o)
Bacteriodales (o)
Lachnospiraceae (f)
Lachnospiraceae (f)
Lachnospiraceae (f)
Lachnospiraceae (f)
Flavonifractor (g)
Bacteriodales (o)
Bacteriodales (o)
Lachnospiraceae (f)
Catonella (g)
Bacteriodales (o)
Bacteriodales (o)
Akkermansia (g)
Bacteriodales (o)
Bacteriodales (o)
Akkermansia (g)
Akkermansia (g)
Oscillospiraceae (f)
Lachnospiraceae (f)
Rikenellaceae (f)
Lachnospiraceae (f)
Lachnospiraceae (f)
Lachnospiraceae (f)
Oscillospirales (o)
Bacteriodales (o)
Rikenellaceae (f)
Bacteriodales (o)
Lachnospiraceae (f)
Ruminococcaceae (f)
Firmicutes (p)
E siraeum (s)
Oscillospirales (o)
Bacilli (c)
Bacteriodales (o)
Burkholderiales (o)
Bacteriodales (o)
Burkholderiales (o)
Bacteriodales (o)
Bacteriodales (o)
Burkholderiales (o)
E. siraeum
1
0.5
0
−0.5
−1
Firmicute
Bacteroidota
Verrucomicrobiota
Proteobacteria

H.

AmAc VWR Correlation Difference (Pre minus Post)

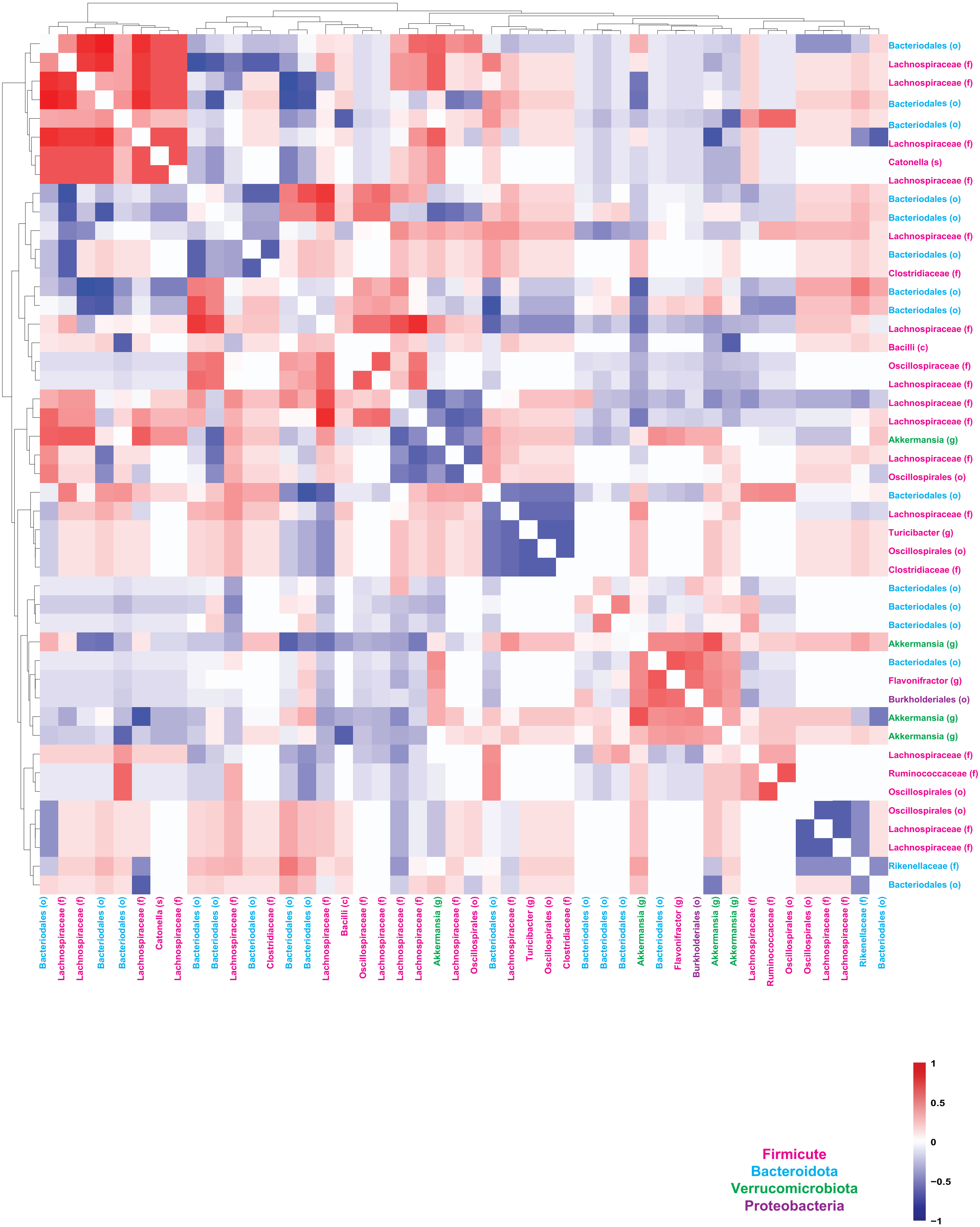

A.

Pre-Intervention Comparisons

AmAc UA vs PBS UA

S. Figure 5

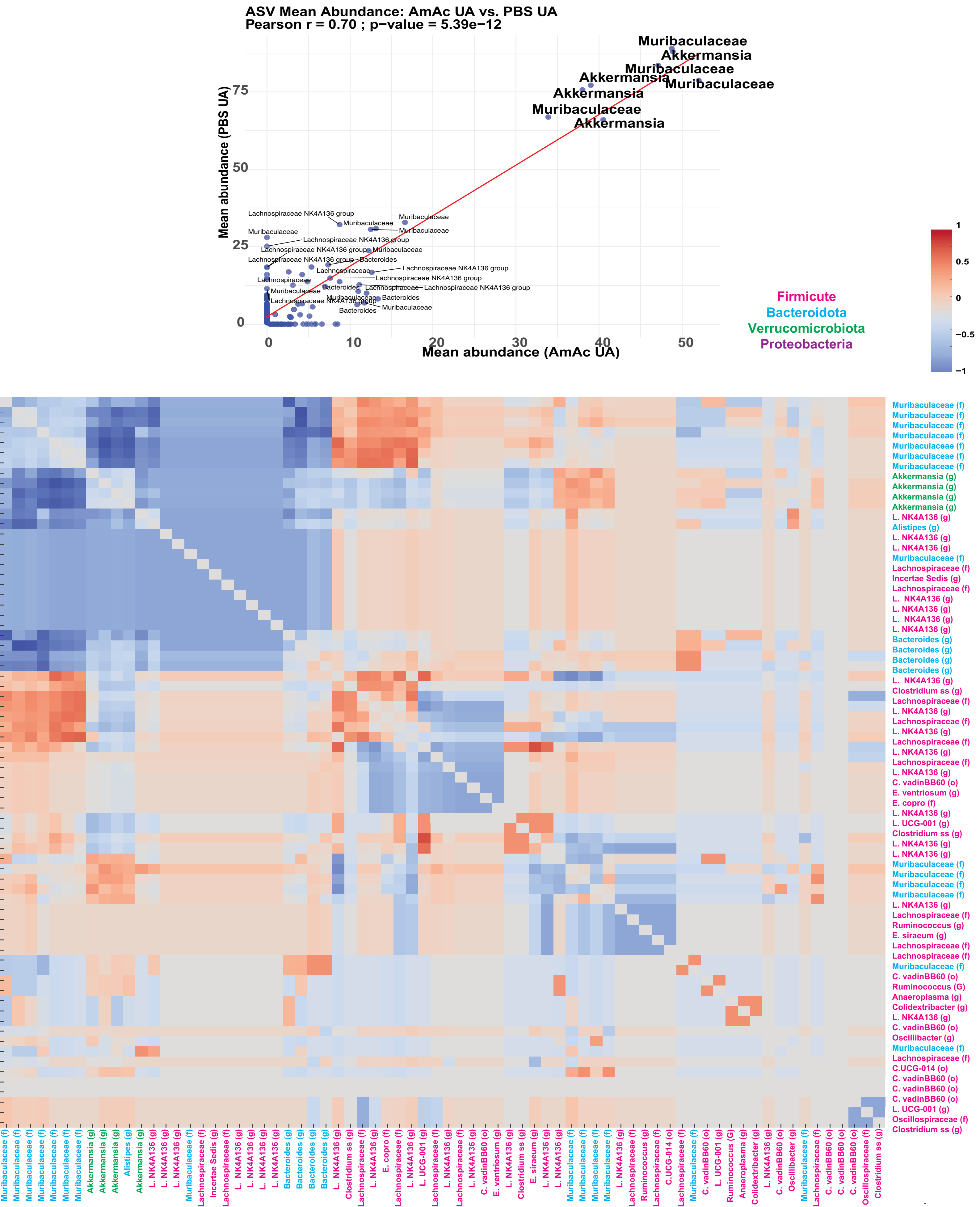

B.

S. Figure 5

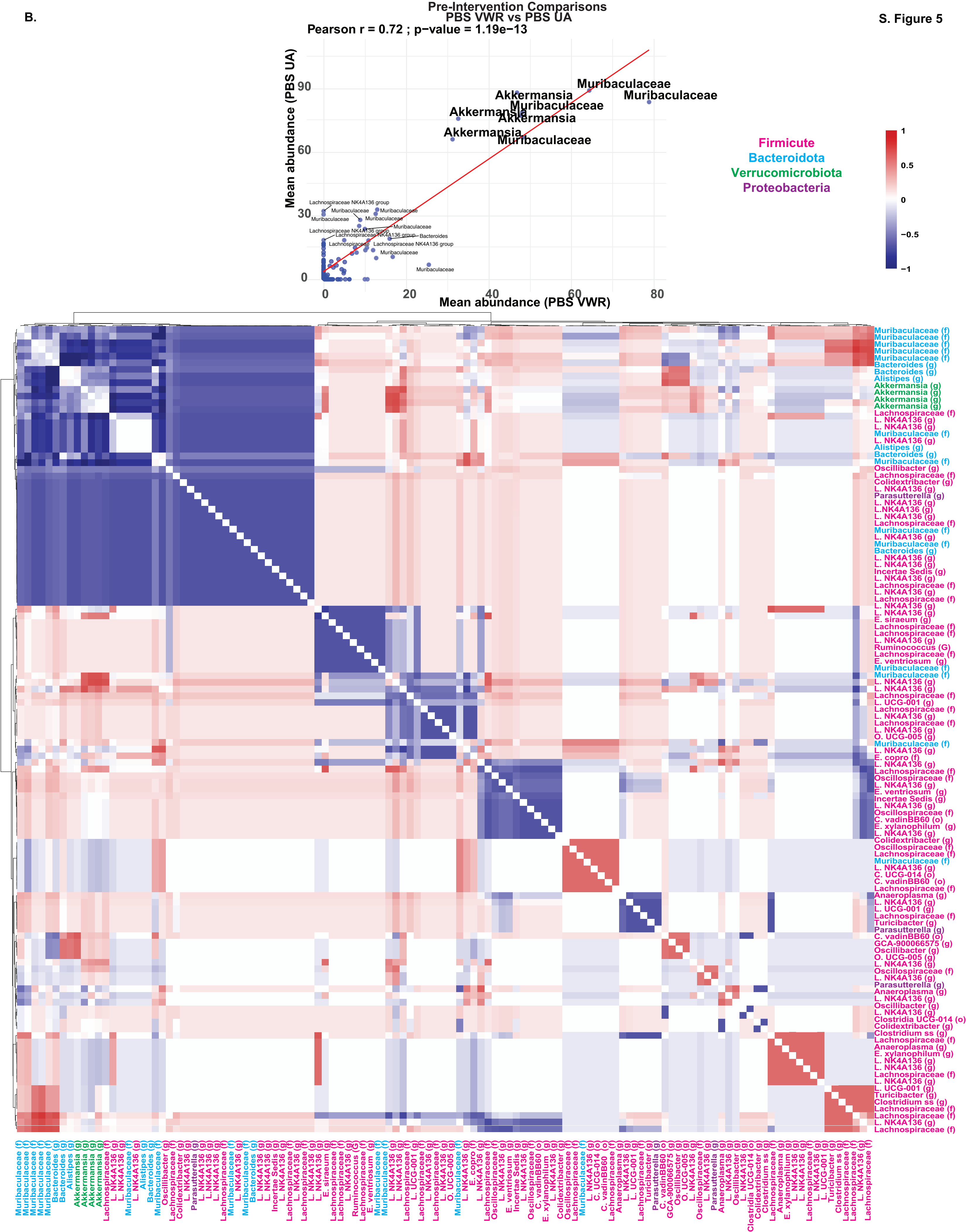
Pre-Intervention Comparisons
PBS VWR vs PBS UA
Pearson r = 0.72 ; p−value = 1.19e−13
Mean abundance (PBS UA)
Mean abundance (PBS VWR)
Muribaculaceae
Akkermansia
Lachnospiraceae NK4A136 group
Bacteroides
Firmicute
Bacteroidota
Verrucomicrobiota
Proteobacteria

C.

Pre-Intervention Comparisons
AmAc VWR vs PBS VWR

S. Figure 5

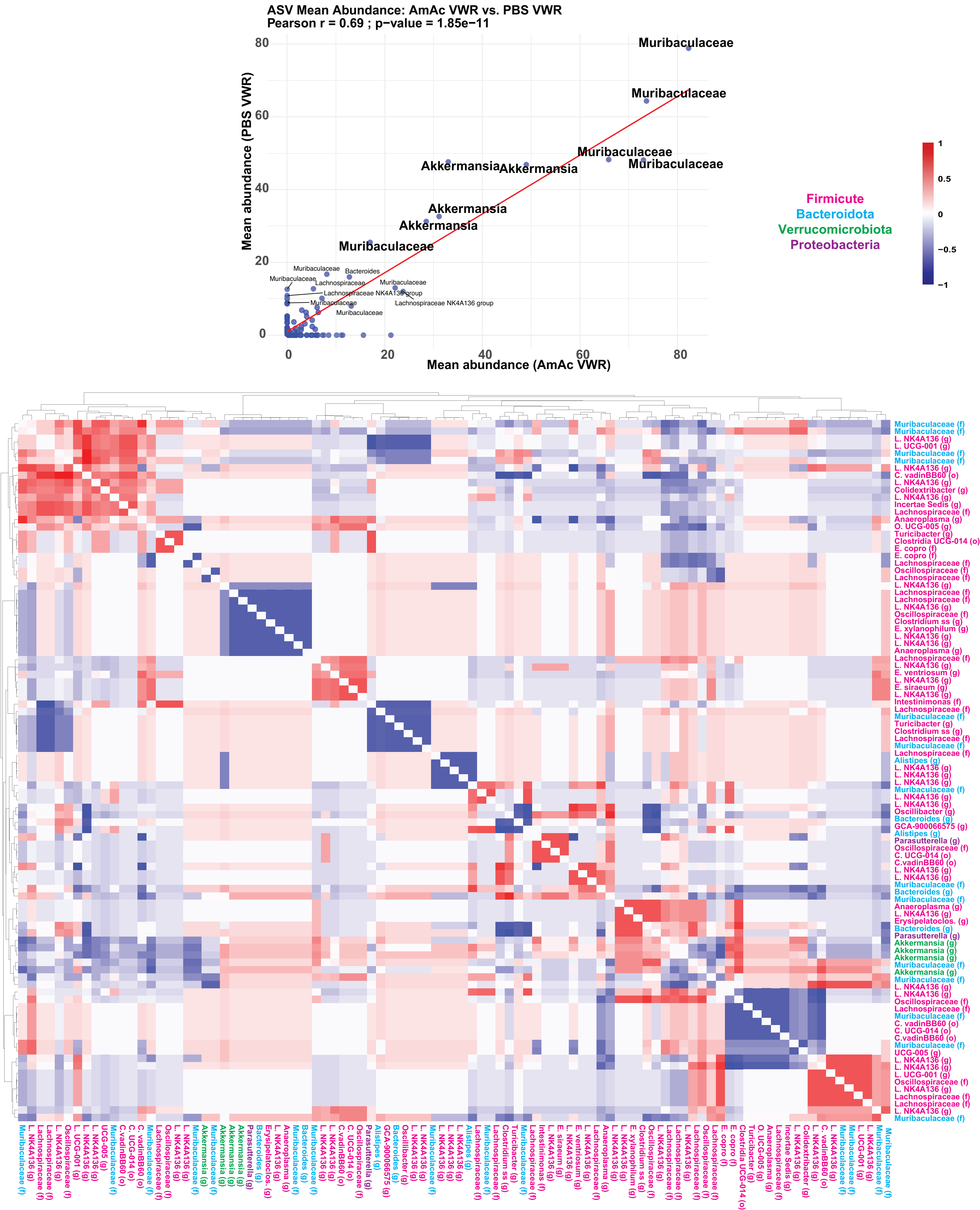

D.

S. Figure 5

Pre-Intervention Comparisons
AmAc VWR vs AmAc UA

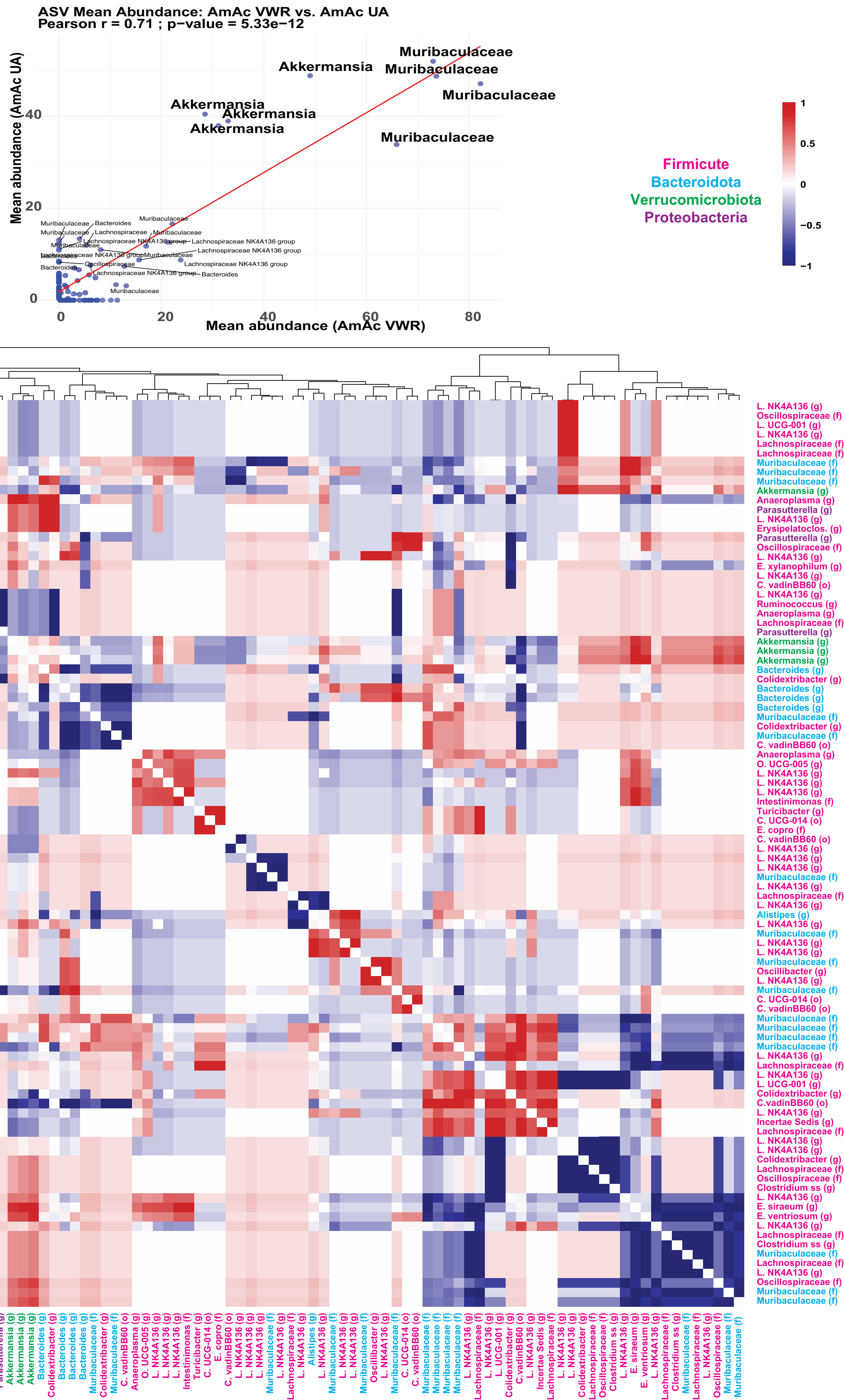

A.

Post-Intervention
AmAc UA vs PBS UA Correlation Plots

S. Figure 6

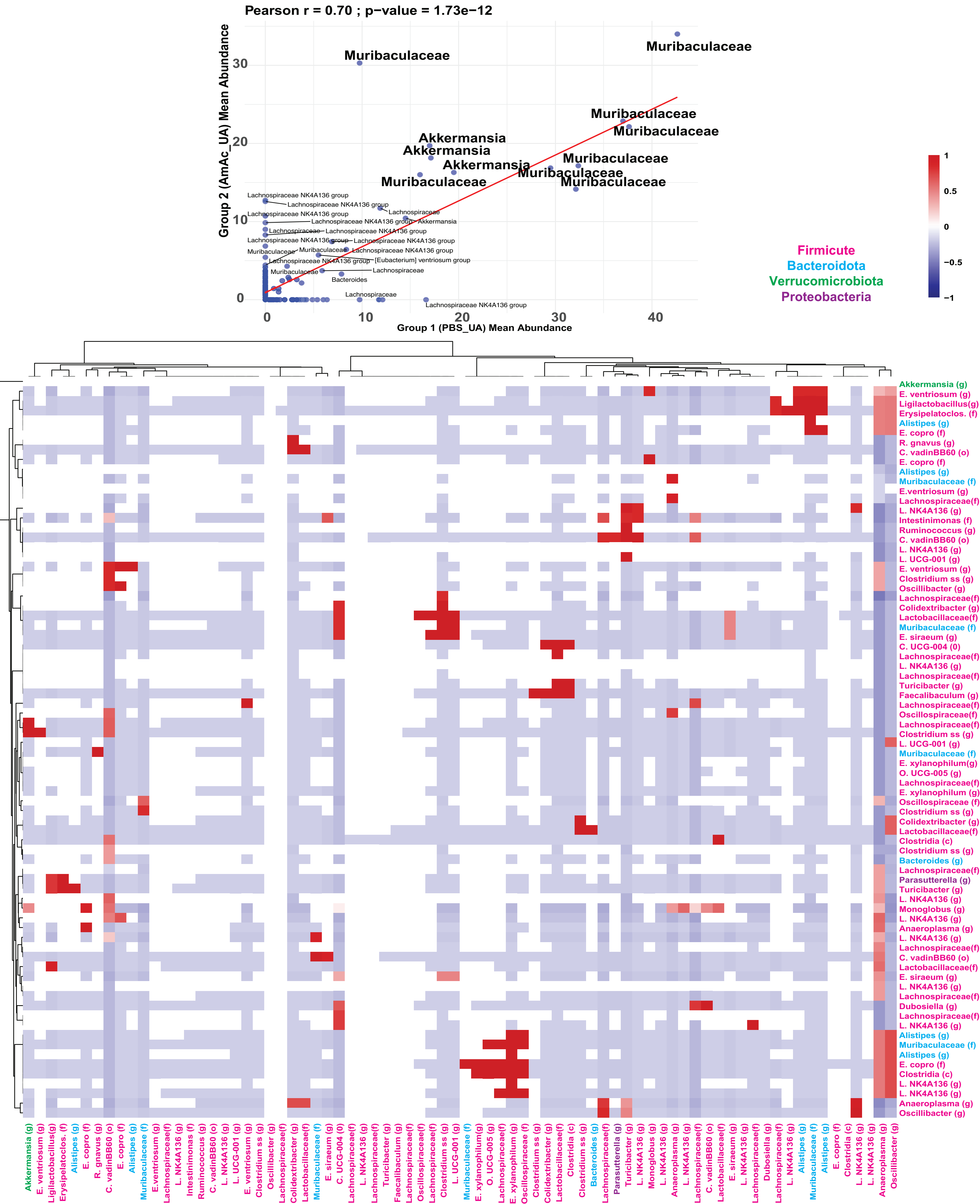

B.

S. Figure 6

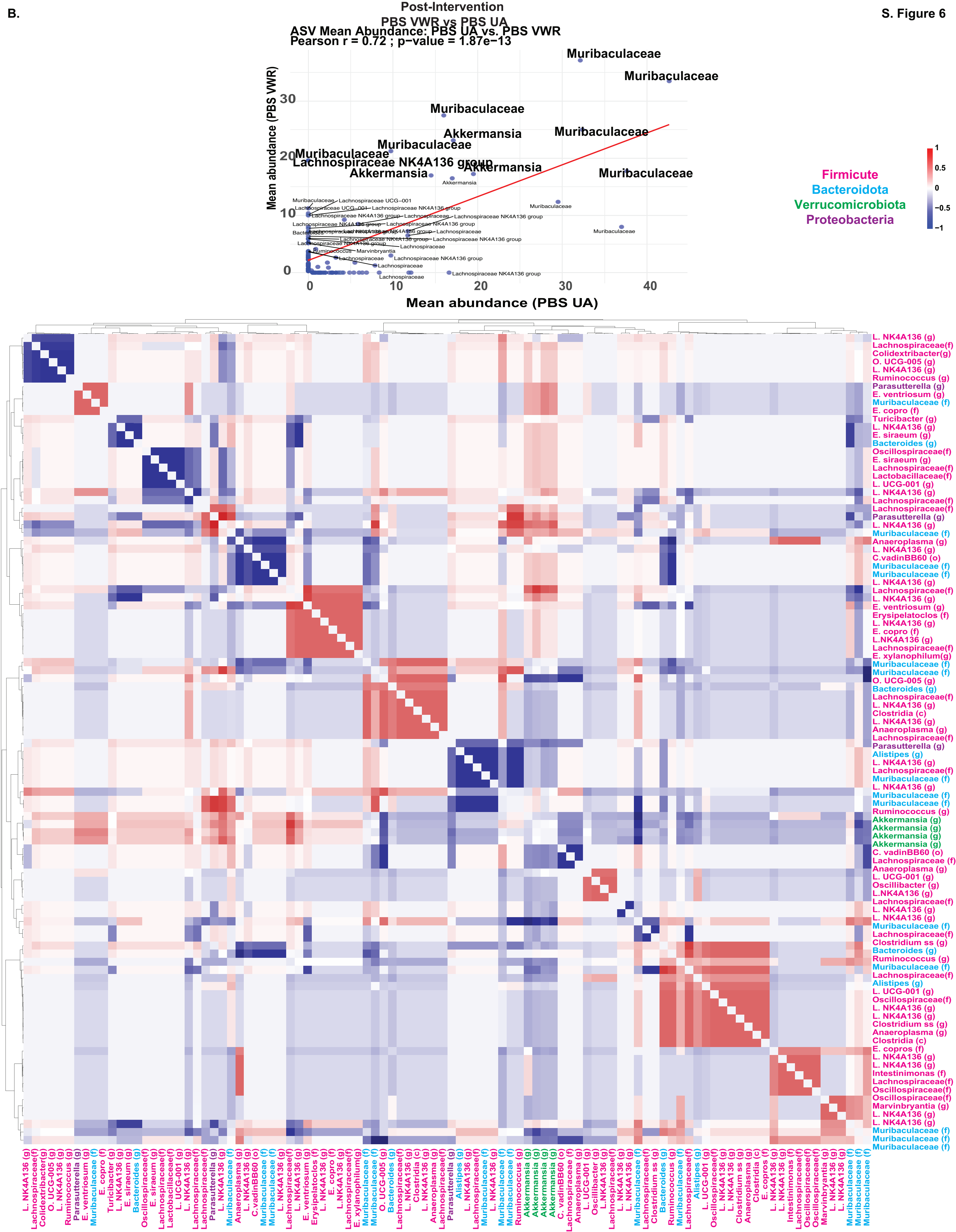

Post-Intervention
PBS VWR vs PBS UA
ASV Mean Abundance: PBS UA vs. PBS VWR
Pearson r = 0.72 ; p-value = 1.87e−13
Mean abundance (PBS VWR)
Mean abundance (PBS UA)
Muribaculaceae
Akkermansia
Lachnospiraceae NK4A136 group
Firmicute
Bacteroidota
Verrucomicrobiota
Proteobacteria

C.

S. Figure 6

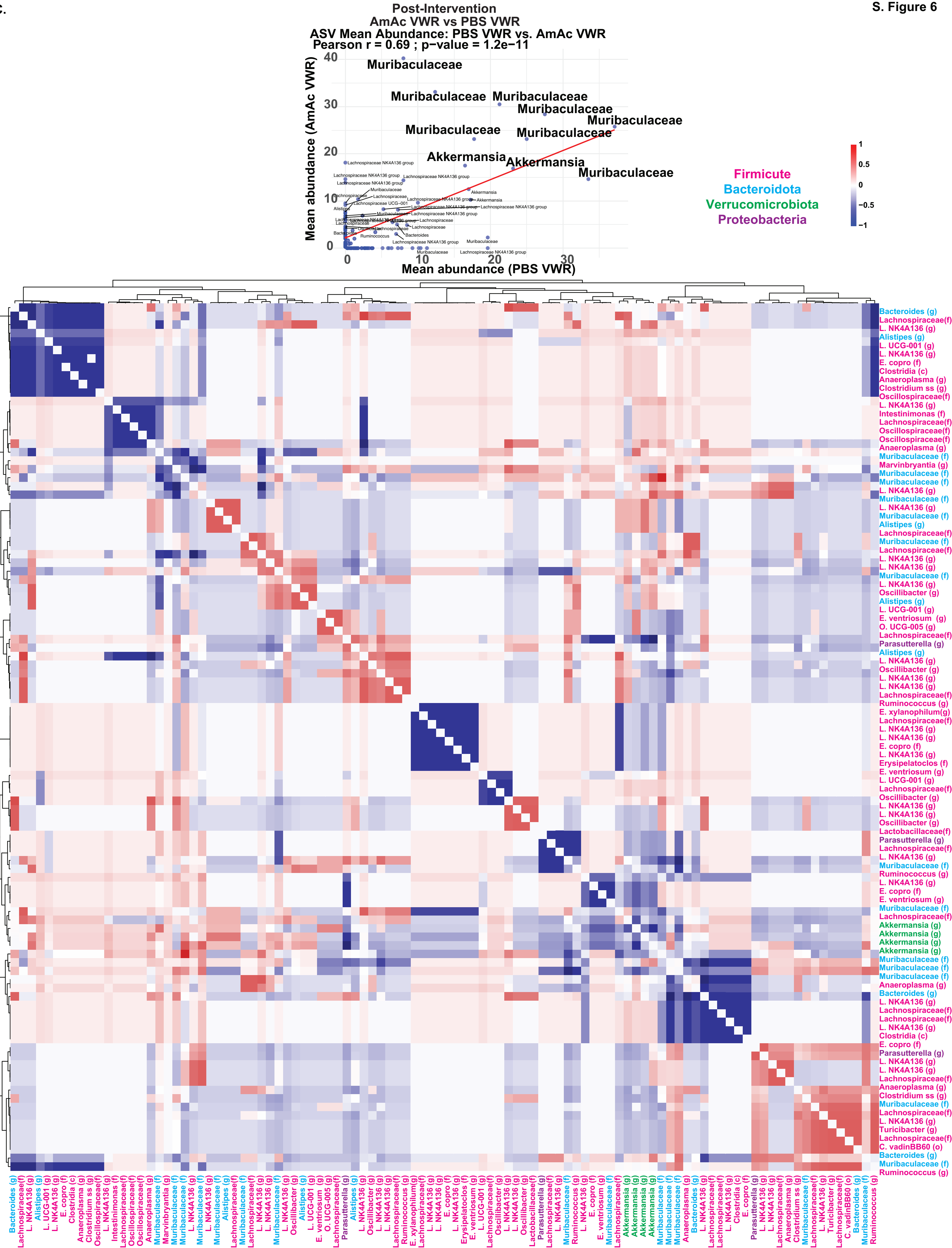

Post-Intervention
AmAc VWR vs PBS VWR
ASV Mean Abundance: PBS VWR vs. AmAc VWR
Pearson r = 0.69 ; p-value = 1.2e-11
Mean abundance (AmAc VWR)
Mean abundance (PBS VWR)
Muribaculaceae
Akkermansia
Firmicute
Bacteroidota
Verrucomicrobiota
Proteobacteria
1
0.5
0
−0.5
−1
Bacteroides (g)
Lachnospiraceae(f)
L. NK4A136 (g)
Alistipes (g)
L. UCG-001 (g)
L. NK4A136 (g)
E. copro (f)
Clostridia (c)
Anaeroplasma (g)
Clostridium ss (g)
Oscillospiraceae(f)
L. NK4A136 (g)
Intestinimonas (f)
Lachnospiraceae(f)
Oscillospiraceae(f)
Oscillospiraceae(f)
Anaeroplasma (g)
Muribaculaceae (f)
Marvinbryantia (g)
Muribaculaceae (f)
Muribaculaceae (f)
L. NK4A136 (g)
Muribaculaceae (f)
L. NK4A136 (g)
Muribaculaceae (f)
Alistipes (g)
Lachnospiraceae(f)
Muribaculaceae (f)
Lachnospiraceae(f)
L. NK4A136 (g)
L. NK4A136 (g)
Muribaculaceae (f)
L. NK4A136 (g)
Oscillibacter (g)
Alistipes (g)
L. UCG-001 (g)
E. ventriosum (g)
O. UCG-005 (g)
Lachnospiraceae(f)
Parasutterella (g)
Alistipes (g)
L. NK4A136 (g)
Oscillibacter (g)
L. NK4A136 (g)
L. NK4A136 (g)
Lachnospiraceae(f)
Ruminococcus (g)
E. xylanophilum(g)
Lachnospiraceae(f)
L. NK4A136 (g)
L. NK4A136 (g)
E. copro (f)
L. NK4A136 (g)
Erysipelatoclos (f)
E. ventriosum (g)
L. UCG-001 (g)
Lachnospiraceae(f)
Oscillibacter (g)
L. NK4A136 (g)
L. NK4A136 (g)
Oscillibacter (g)
Lactobacillaceae(f)
Parasutterella (g)
Lachnospiraceae(f)
L. NK4A136 (g)
Muribaculaceae (f)
Ruminococcus (g)
L. NK4A136 (g)
E. copro (f)
E. ventriosum (g)
Muribaculaceae (f)
Lachnospiraceae(f)
Akkermansia (g)
Akkermansia (g)
Akkermansia (g)
Akkermansia (g)
Muribaculaceae (f)
Muribaculaceae (f)
Muribaculaceae (f)
Anaeroplasma (g)
Bacteroides (g)
L. NK4A136 (g)
Lachnospiraceae(f)
Lachnospiraceae(f)
L. NK4A136 (g)
Clostridia (c)
E. copro (f)
Parasutterella (g)
L. NK4A136 (g)
L. NK4A136 (g)
Lachnospiraceae(f)
Anaeroplasma (g)
Clostridium ss (g)
Muribaculaceae (f)
Lachnospiraceae(f)
L. NK4A136 (g)
Turicibacter (g)
Lachnospiraceae(f)
C. vadinBB60 (o)
Bacteroides (g)
Muribaculaceae (f)
Ruminococcus (g)

D.

S. Figure 6

Post-Intervention
AmAc VWR vs AmAc UA

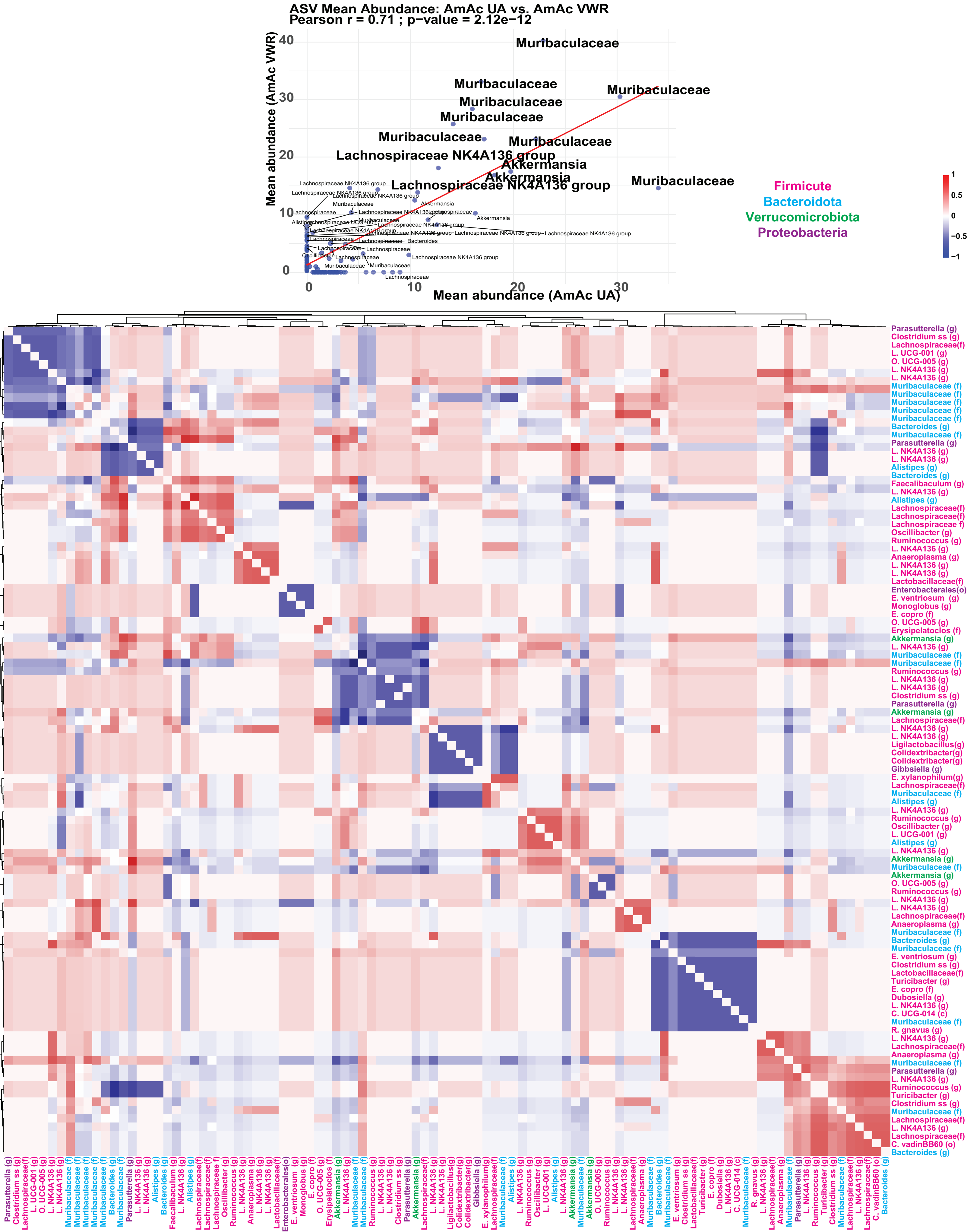

A.

S. Figure 7

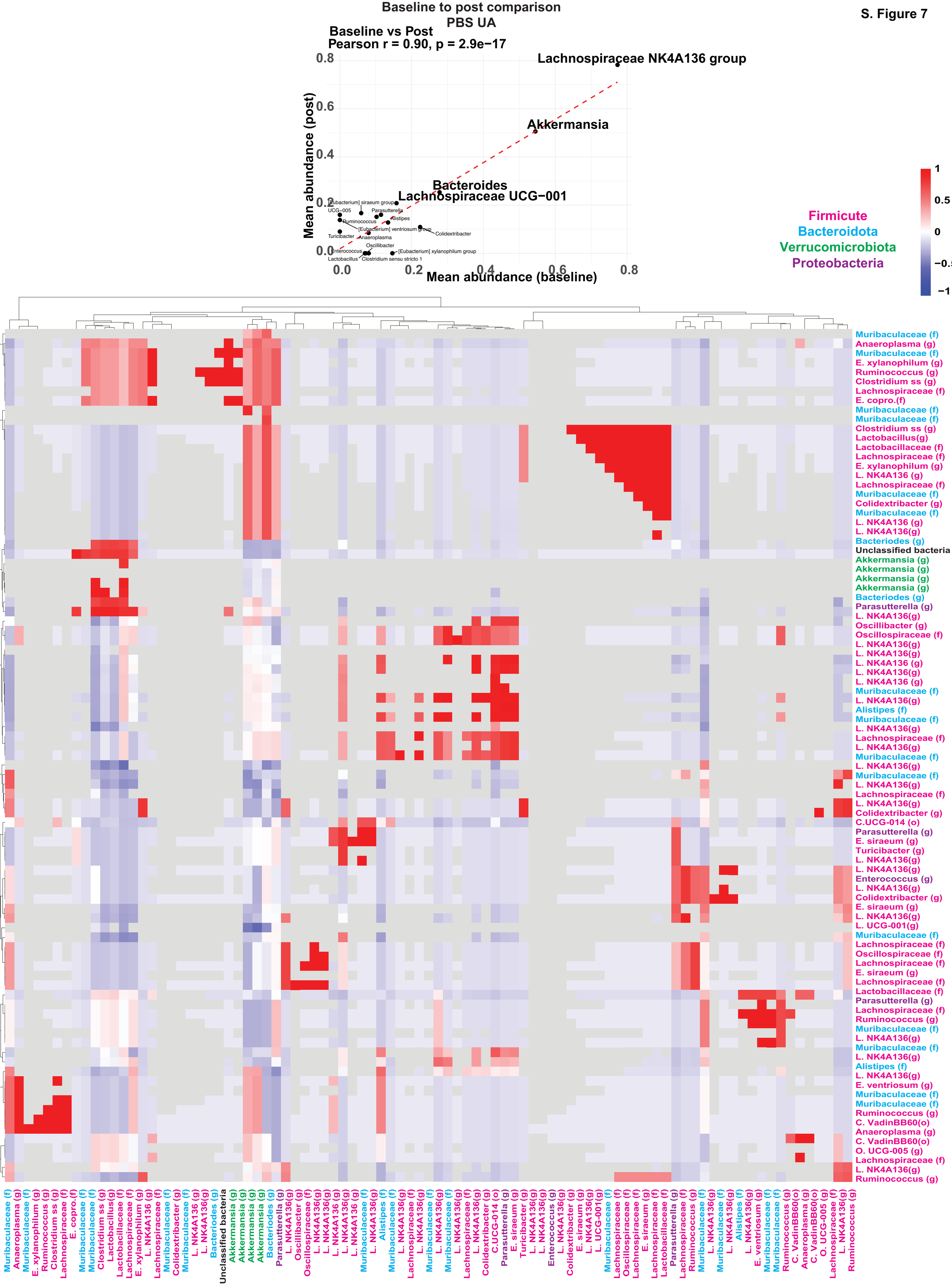
Baseline to post comparison
PBS UA
Baseline vs Post
Pearson r = 0.90, p = 2.9e−17
Lachnospiraceae NK4A136 group
Akkermansia
Bacteroides
Lachnospiraceae UCG−001
[Eubacterium] siraeum group
UCG−005
Parasutterella
Alistipes
Ruminococcus
[Eubacterium] ventriosum group
Colidextribacter
Turicibacter
Anaeroplasma
Oscillibacter
Enterococcus
[Eubacterium] xylanophilum group
Lactobacillus
Clostridium sensu stricto 1
Mean abundance (post)
Mean abundance (baseline)
0.0
0.2
0.4
0.6
0.8
1
0.5
0
−0.5
−1
Firmicute
Bacteroidota
Verrucomicrobiota
Proteobacteria
Muribaculaceae (f)
Anaeroplasma (g)
Muribaculaceae (f)
E. xylanophilum (g)
Ruminococcus (g)
Clostridium ss (g)
Lachnospiraceae (f)
E. copro.(f)
Muribaculaceae (f)
Muribaculaceae (f)
Clostridium ss (g)
Lactobacillus(g)
Lactobacillaceae (f)
Lachnospiraceae (f)
E. xylanophilum (g)
L. NK4A136 (g)
Lachnospiraceae (f)
Muribaculaceae (f)
Colidextribacter (g)
Muribaculaceae (f)
L. NK4A136 (g)
L. NK4A136(g)
Bacteriodes (g)
Unclassified bacteria
Akkermansia (g)
Akkermansia (g)
Akkermansia (g)
Akkermansia (g)
Bacteriodes (g)
Parasutterella (g)
L. NK4A136(g)
Oscillibacter (g)
Oscillospiraceae (f)
L. NK4A136(g)
L. NK4A136(g)
L. NK4A136 (g)
L. NK4A136(g)
L. NK4A136 (g)
Muribaculaceae (f)
L. NK4A136(g)
Alistipes (f)
Muribaculaceae (f)
L. NK4A136(g)
Lachnospiraceae (f)
L. NK4A136(g)
Muribaculaceae (f)
L. NK4A136(g)
Muribaculaceae (f)
L. NK4A136(g)
Lachnospiraceae (f)
L. NK4A136(g)
Colidextribacter (g)
C.UCG-014 (o)
Parasutterella (g)
E. siraeum (g)
Turicibacter (g)
L. NK4A136(g)
L. NK4A136(g)
Enterococcus (g)
L. NK4A136(g)
Colidextribacter (g)
E. siraeum (g)
L. NK4A136(g)
L. UCG-001(g)
Muribaculaceae (f)
Lachnospiraceae (f)
Oscillospiraceae (f)
Lachnospiraceae (f)
E. siraeum (g)
Lachnospiraceae (f)
Lactobacillaceae (f)
Parasutterella (g)
Lachnospiraceae (f)
Ruminococcus (g)
Muribaculaceae (f)
L. NK4A136(g)
Muribaculaceae (f)
L. NK4A136(g)
Alistipes (f)
L. NK4A136(g)
E. ventriosum (g)
Muribaculaceae (f)
Muribaculaceae (f)
Ruminococcus (g)
C. VadinBB60(o)
Anaeroplasma (g)
C. VadinBB60(o)
O. UCG-005 (g)
Lachnospiraceae (f)
L. NK4A136(g)
Ruminococcus (g)

B.

S. Figure 7

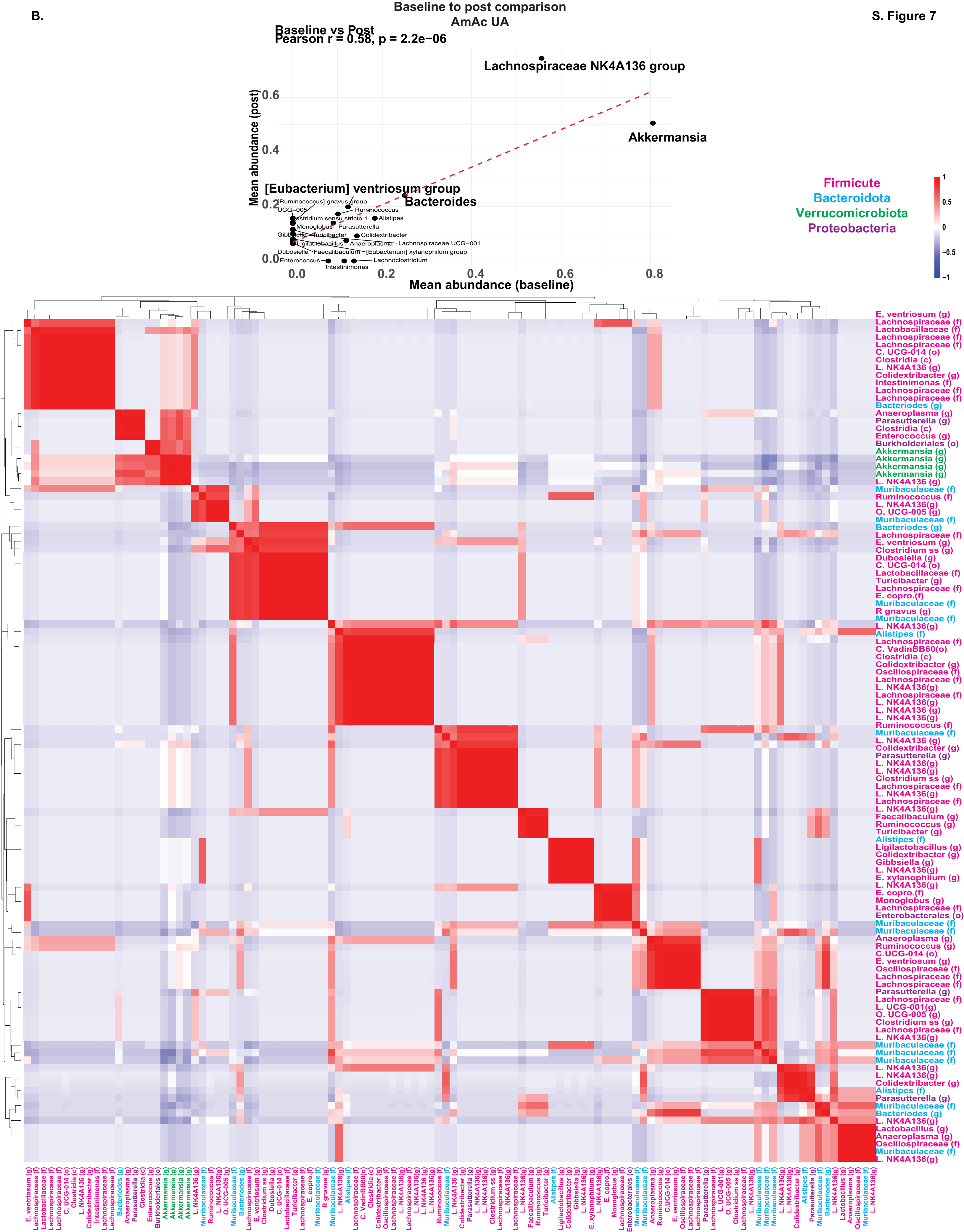

Baseline to post comparison
AmAc UA
Baseline vs Post
Pearson r = 0.58, p = 2.2e−06
Lachnospiraceae NK4A136 group
Akkermansia
[Eubacterium] ventriosum group
Bacteroides
[Ruminococcus] gnavus group
UCG−005
Ruminococcus
Clostridium sensu stricto 1
Alistipes
Monoglobus
Parasutterella
Gibbsiella
Turicibacter
Colidextribacter
Ligilactobacillus
Anaeroplasma
Lachnospiraceae UCG−001
Dubosiella
Faecalibaculum
[Eubacterium] xylanophilum group
Enterococcus
Intestinimonas
Lachnoclostridium
Mean abundance (post)
Mean abundance (baseline)
0.0
0.2
0.4
0.6
0.8
Firmicute
Bacteroidota
Verrucomicrobiota
Proteobacteria
1
0.5
0
−0.5
−1
E. ventriosum (g)
Lachnospiraceae (f)
Lactobacillaceae (f)
Lachnospiraceae (f)
Lachnospiraceae (f)
C. UCG-014 (o)
Clostridia (c)
L. NK4A136 (g)
Colidextribacter (g)
Intestinimonas (f)
Lachnospiraceae (f)
Lachnospiraceae (f)
Bacteriodes (g)
Anaeroplasma (g)
Parasutterella (g)
Clostridia (c)
Enterococcus (g)
Burkholderiales (o)
Akkermansia (g)
Akkermansia (g)
Akkermansia (g)
Akkermansia (g)
L. NK4A136 (g)
Muribaculaceae (f)
Ruminococcus (f)
L. NK4A136(g)
O. UCG-005 (g)
Muribaculaceae (f)
Bacteriodes (g)
Lachnospiraceae (f)
E. ventriosum (g)
Clostridium ss (g)
Dubosiella (g)
C. UCG-014 (o)
Lactobacillaceae (f)
Turicibacter (g)
Lachnospiraceae (f)
E. copro.(f)
Muribaculaceae (f)
R gnavus (g)
Muribaculaceae (f)
L. NK4A136(g)
Alistipes (f)
Lachnospiraceae (f)
C. VadinBB60(o)
Clostridia (c)
Colidextribacter (g)
Oscillospiraceae (f)
Lachnospiraceae (f)
L. NK4A136(g)
Lachnospiraceae (f)
L. NK4A136(g)
L. NK4A136 (g)
L. NK4A136(g)
Ruminococcus (f)
Muribaculaceae (f)
L. NK4A136 (g)
Colidextribacter (g)
Parasutterella (g)
L. NK4A136(g)
L. NK4A136(g)
Clostridium ss (g)
Lachnospiraceae (f)
L. NK4A136(g)
Lachnospiraceae (f)
L. NK4A136(g)
Faecalibaculum (g)
Ruminococcus (g)
Turicibacter (g)
Alistipes (f)
Ligilactobacillus (g)
Colidextribacter (g)
Gibbsiella (g)
L. NK4A136(g)
E. xylanophilum (g)
L. NK4A136(g)
E. copro.(f)
Monoglobus (g)
Lachnospiraceae (f)
Enterobacterales (o)
Muribaculaceae (f)
Muribaculaceae (f)
Anaeroplasma (g)
Ruminococcus (g)
C.UCG-014 (o)
E. ventriosum (g)
Oscillospiraceae (f)
Lachnospiraceae (f)
Lachnospiraceae (f)
Parasutterella (g)
Lachnospiraceae (f)
L. UCG-001(g)
O. UCG-005 (g)
Clostridium ss (g)
Lachnospiraceae (f)
L. NK4A136(g)
Muribaculaceae (f)
Muribaculaceae (f)
Muribaculaceae (f)
L. NK4A136(g)
L. NK4A136(g)
Colidextribacter (g)
Alistipes (f)
Parasutterella (g)
Muribaculaceae (f)
Bacteriodes (g)
L. NK4A136(g)
Lactobacillus (g)
Anaeroplasma (g)
Oscillospiraceae (f)
Muribaculaceae (f)
L. NK4A136(g)

C.

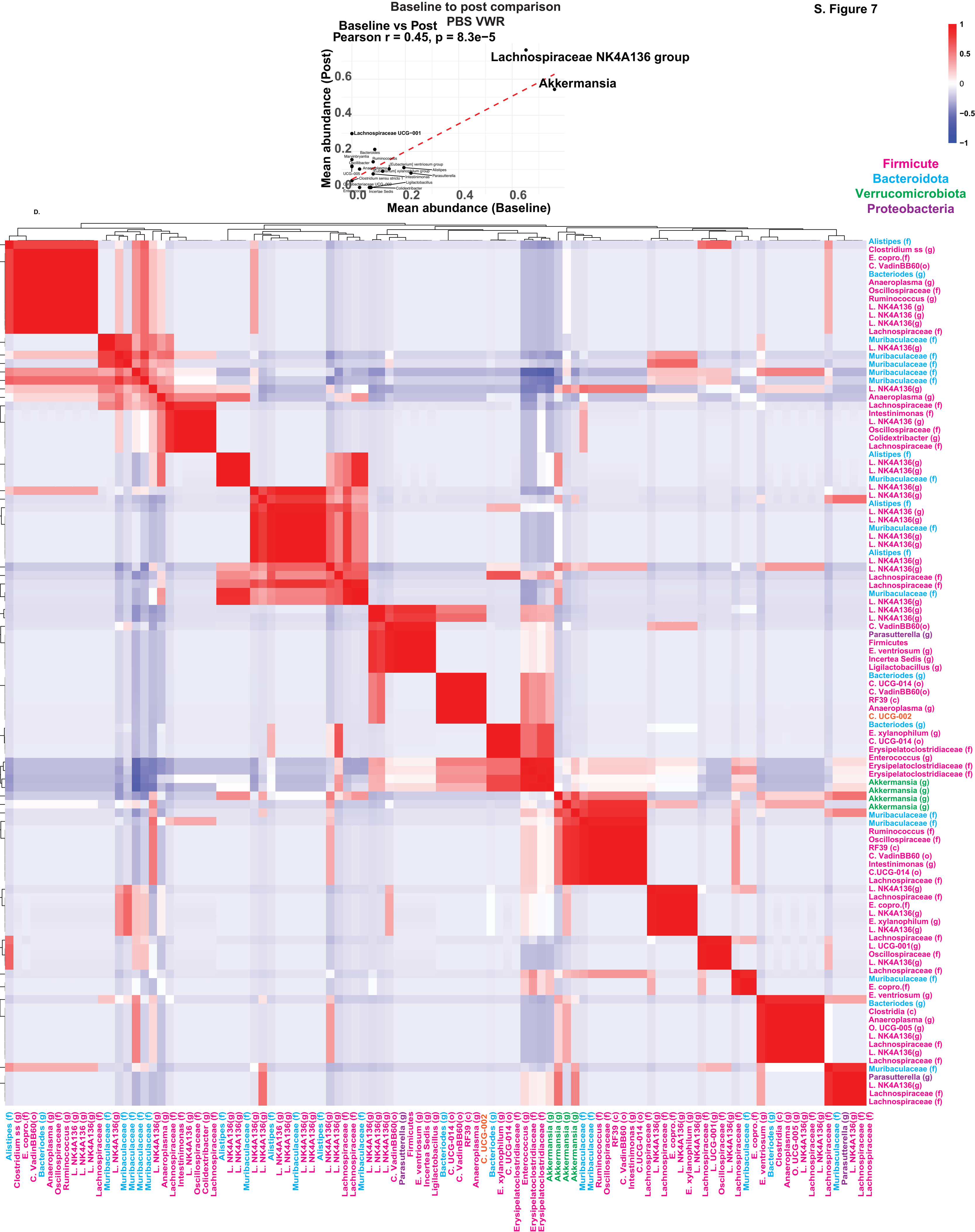
S. Figure 7
Baseline to post comparison
PBS VWR
Baseline vs Post
Pearson r = 0.45, p = 8.3e−5
Lachnospiraceae NK4A136 group
Akkermansia
Lachnospiraceae UCG-001
Mean abundance (Post)
Mean abundance (Baseline)
0.0
0.2
0.4
0.6
1
0.5
0
−0.5
−1
Firmicute
Bacteroidota
Verrucomicrobiota
Proteobacteria
D.
Alistipes (f)
Clostridium ss (g)
E. copro.(f)
C. VadinBB60(o)
Bacteriodes (g)
Anaeroplasma (g)
Oscillospiraceae (f)
Ruminococcus (g)
L. NK4A136 (g)
L. NK4A136 (g)
L. NK4A136(g)
Lachnospiraceae (f)
Muribaculaceae (f)
L. NK4A136(g)
Muribaculaceae (f)
Muribaculaceae (f)
Muribaculaceae (f)
Muribaculaceae (f)
L. NK4A136(g)
Anaeroplasma (g)
Lachnospiraceae (f)
Intestinimonas (f)
L. NK4A136 (g)
Oscillospiraceae (f)
Colidextribacter (g)
Lachnospiraceae (f)
Alistipes (f)
L. NK4A136(g)
L. NK4A136(g)
Muribaculaceae (f)
L. NK4A136(g)
L. NK4A136(g)
Alistipes (f)
L. NK4A136 (g)
L. NK4A136(g)
Muribaculaceae (f)
L. NK4A136(g)
L. NK4A136(g)
Alistipes (f)
L. NK4A136(g)
L. NK4A136(g)
Lachnospiraceae (f)
Lachnospiraceae (f)
Muribaculaceae (f)
L. NK4A136(g)
L. NK4A136(g)
L. NK4A136(g)
C. VadinBB60(o)
Parasutterella (g)
Firmicutes
E. ventriosum (g)
Incertea Sedis (g)
Ligilactobacillus (g)
Bacteriodes (g)
C. UCG-014 (o)
C. VadinBB60(o)
RF39 (c)
Anaeroplasma (g)
C. UCG-002
Bacteriodes (g)
E. xylanophilum (g)
C. UCG-014 (o)
Erysipelatoclostridiaceae (f)
Enterococcus (g)
Erysipelatoclostridiaceae (f)
Erysipelatoclostridiaceae (f)
Akkermansia (g)
Akkermansia (g)
Akkermansia (g)
Akkermansia (g)
Muribaculaceae (f)
Muribaculaceae (f)
Ruminococcus (f)
Oscillospiraceae (f)
RF39 (c)
C. VadinBB60 (o)
Intestinimonas (g)
C.UCG-014 (o)
Lachnospiraceae (f)
L. NK4A136(g)
Lachnospiraceae (f)
E. copro.(f)
L. NK4A136(g)
E. xylanophilum (g)
L. NK4A136(g)
Lachnospiraceae (f)
L. UCG-001(g)
Oscillospiraceae (f)
L. NK4A136(g)
Lachnospiraceae (f)
Muribaculaceae (f)
E. copro.(f)
E. ventriosum (g)
Bacteriodes (g)
Clostridia (c)
Anaeroplasma (g)
O. UCG-005 (g)
L. NK4A136(g)
Lachnospiraceae (f)
L. NK4A136(g)
Lachnospiraceae (f)
Muribaculaceae (f)
Parasutterella (g)
L. NK4A136(g)
Lachnospiraceae (f)
Lachnospiraceae (f)

S. Figure 7

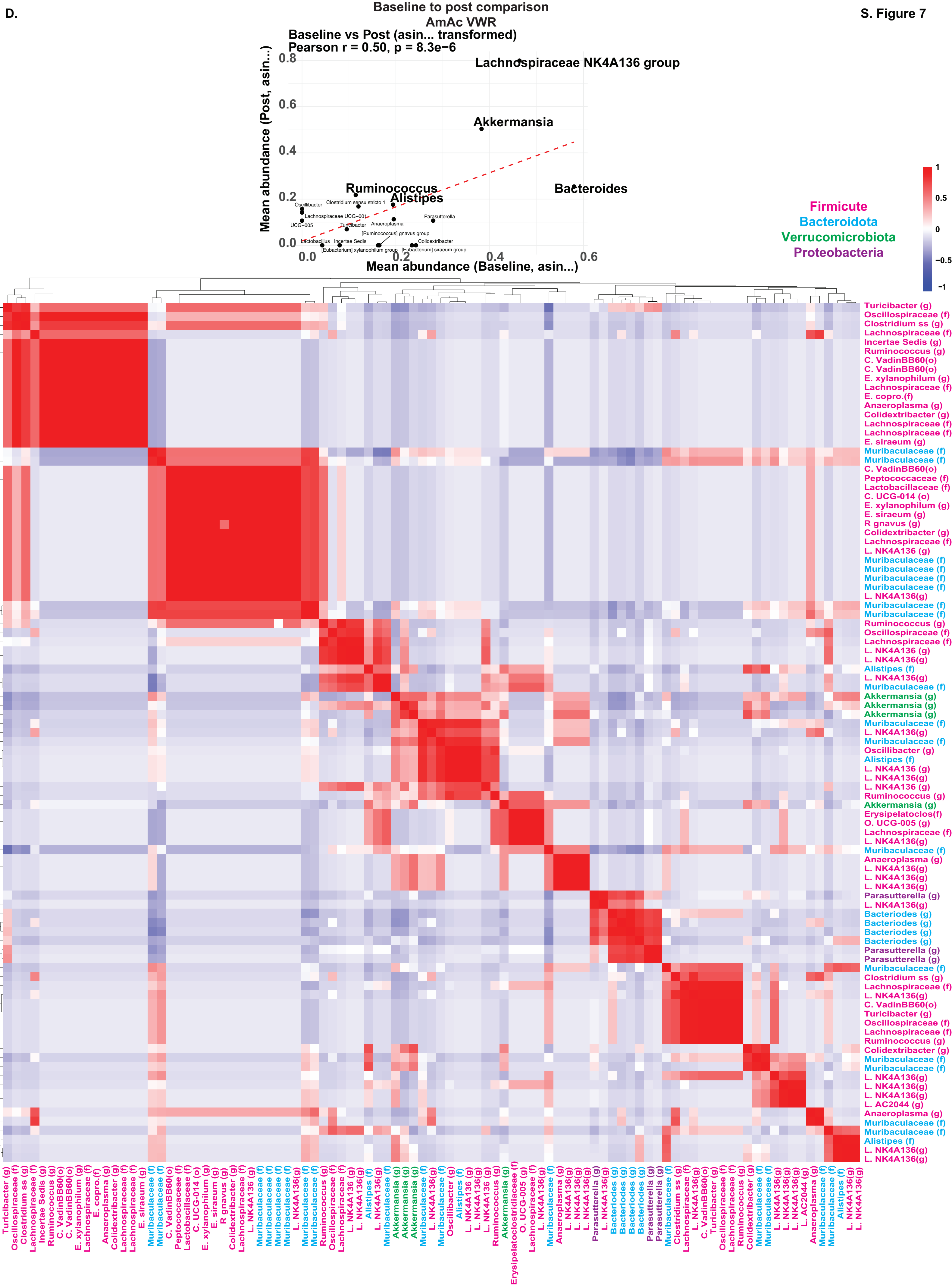
D.
Baseline to post comparison
AmAc VWR
Baseline vs Post (asin... transformed)
Pearson r = 0.50, p = 8.3e−6
Mean abundance (Post, asin...)
Mean abundance (Baseline, asin...)
0.0
0.2
0.4
0.6
0.8
Lachnospiraceae NK4A136 group
Akkermansia
Ruminococcus
Alistipes
Bacteroides
Oscillibacter
Clostridium sensu stricto 1
Lachnospiraceae UCG−001
UCG−005
Turicibacter
Anaeroplasma
Parasutterella
[Ruminococcus] gnavus group
Lactobacillus
Incertae Sedis
Colidextribacter
[Eubacterium] xylanophilum group
[Eubacterium] siraeum group
1
0.5
0
−0.5
−1
Firmicute
Bacteroidota
Verrucomicrobiota
Proteobacteria
Turicibacter (g)
Oscillospiraceae (f)
Clostridium ss (g)
Lachnospiraceae (f)
Incertae Sedis (g)
Ruminococcus (g)
C. VadinBB60(o)
C. VadinBB60(o)
E. xylanophilum (g)
Lachnospiraceae (f)
E. copro.(f)
Anaeroplasma (g)
Colidextribacter (g)
Lachnospiraceae (f)
Lachnospiraceae (f)
E. siraeum (g)
Muribaculaceae (f)
Muribaculaceae (f)
C. VadinBB60(o)
Peptococcaceae (f)
Lactobacillaceae (f)
C. UCG-014 (o)
E. xylanophilum (g)
E. siraeum (g)
R gnavus (g)
Colidextribacter (g)
Lachnospiraceae (f)
L. NK4A136 (g)
Muribaculaceae (f)
Muribaculaceae (f)
Muribaculaceae (f)
Muribaculaceae (f)
L. NK4A136(g)
Muribaculaceae (f)
Muribaculaceae (f)
Ruminococcus (g)
Oscillospiraceae (f)
Lachnospiraceae (f)
L. NK4A136 (g)
L. NK4A136(g)
Alistipes (f)
L. NK4A136(g)
Muribaculaceae (f)
Akkermansia (g)
Akkermansia (g)
Akkermansia (g)
Muribaculaceae (f)
L. NK4A136(g)
Muribaculaceae (f)
Oscillibacter (g)
Alistipes (f)
L. NK4A136 (g)
L. NK4A136(g)
L. NK4A136 (g)
Ruminococcus (g)
Akkermansia (g)
Erysipelatoclos(f)
O. UCG-005 (g)
Lachnospiraceae (f)
L. NK4A136(g)
Muribaculaceae (f)
Anaeroplasma (g)
L. NK4A136(g)
L. NK4A136(g)
L. NK4A136(g)
Parasutterella (g)
L. NK4A136(g)
Bacteriodes (g)
Bacteriodes (g)
Bacteriodes (g)
Bacteriodes (g)
Parasutterella (g)
Parasutterella (g)
Muribaculaceae (f)
Clostridium ss (g)
Lachnospiraceae (f)
L. NK4A136(g)
C. VadinBB60(o)
Turicibacter (g)
Oscillospiraceae (f)
Lachnospiraceae (f)
Ruminococcus (g)
Colidextribacter (g)
Muribaculaceae (f)
Muribaculaceae (f)
L. NK4A136(g)
L. NK4A136(g)
L. NK4A136(g)
L. AC2044 (g)
Anaeroplasma (g)
Muribaculaceae (f)
Muribaculaceae (f)
Alistipes (f)
L. NK4A136(g)
L. NK4A136(g)

A. SCFA Producing Bacteria

S. Figure 8

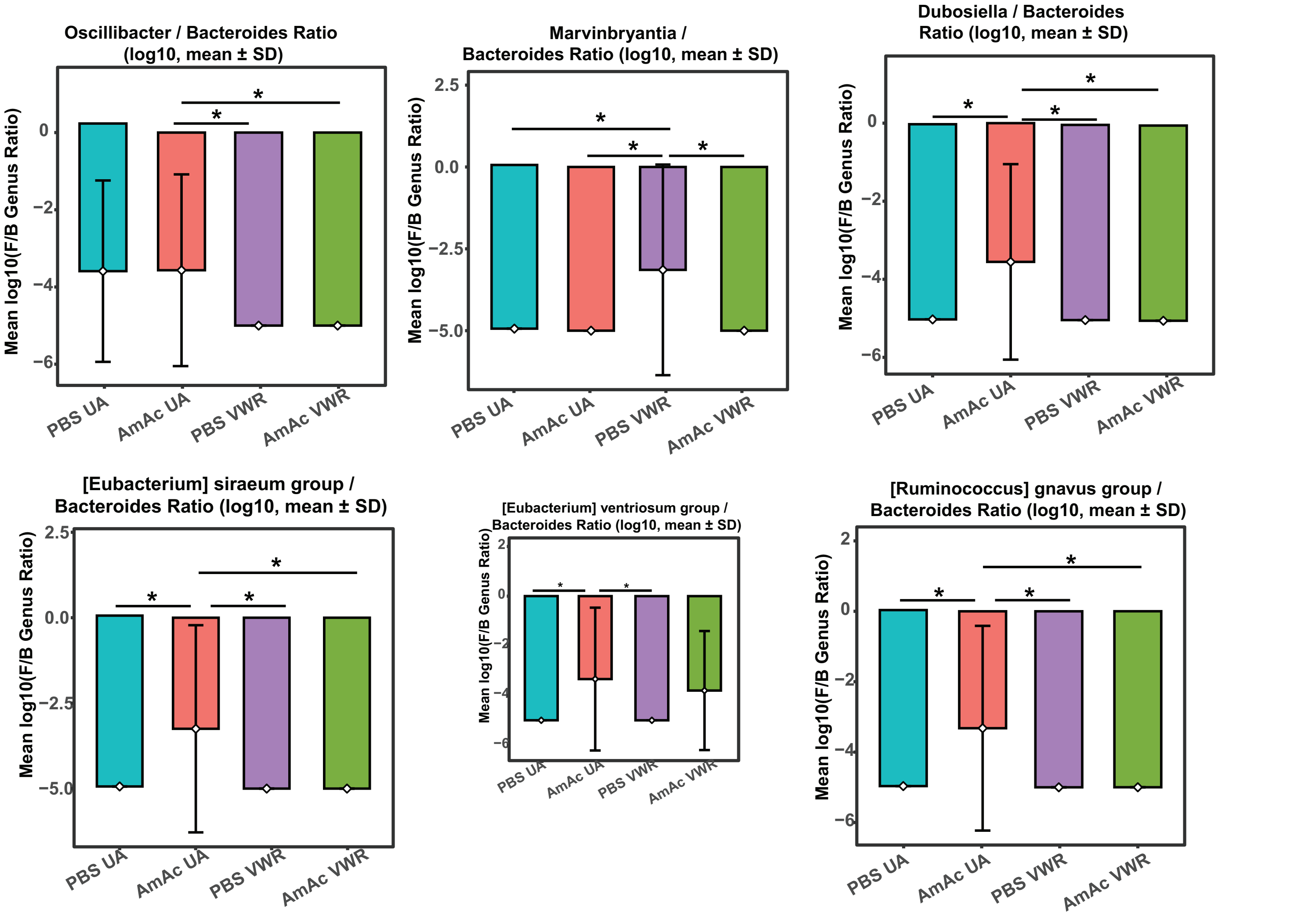

S. Fig. 9

A. PBS UA Post vs Pre PiCrust MetaCyc Pathways

B. PBS UA Post vs Pre PiCrust KO

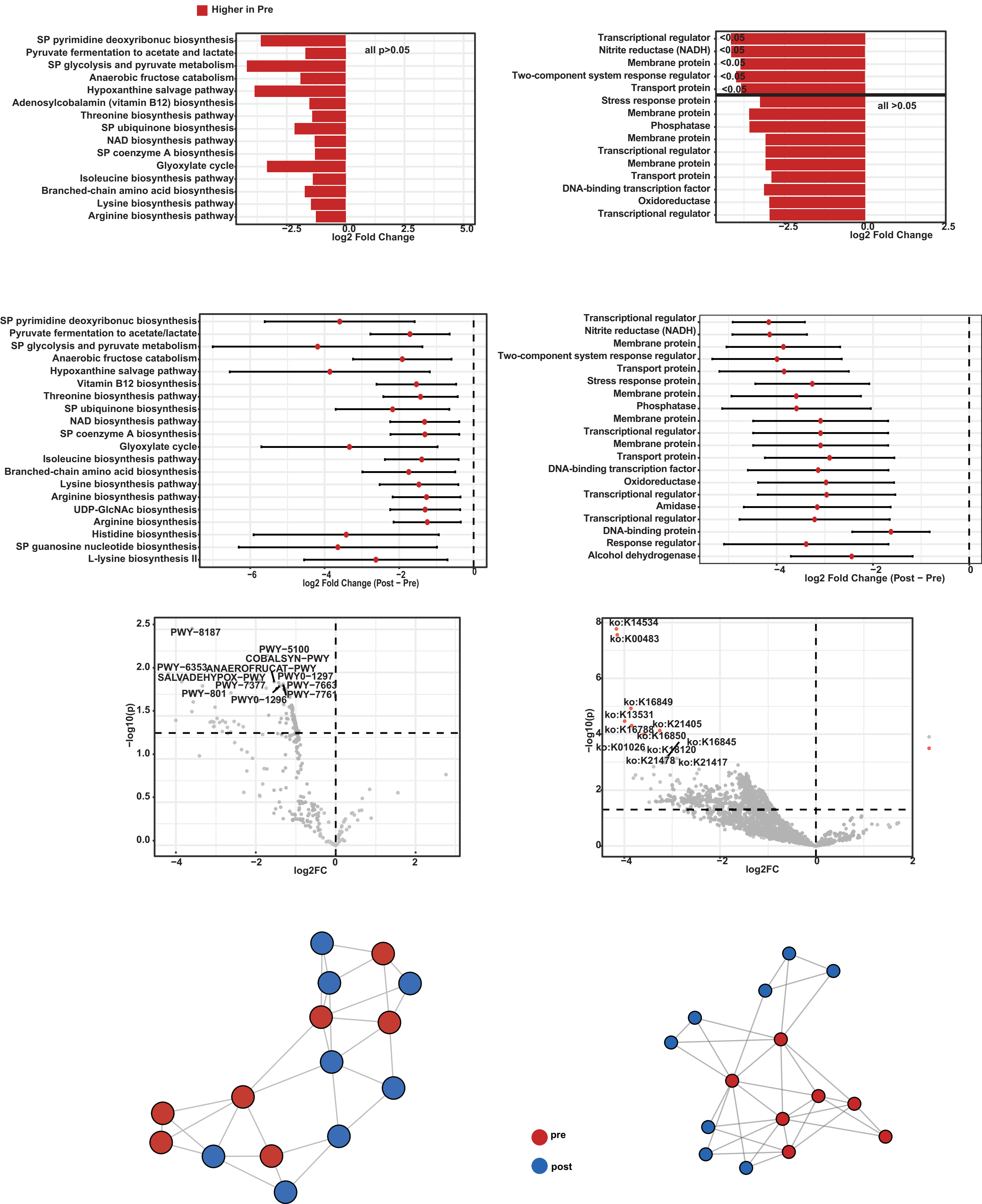

C PBS UA Post vs Pre Tax4Fun

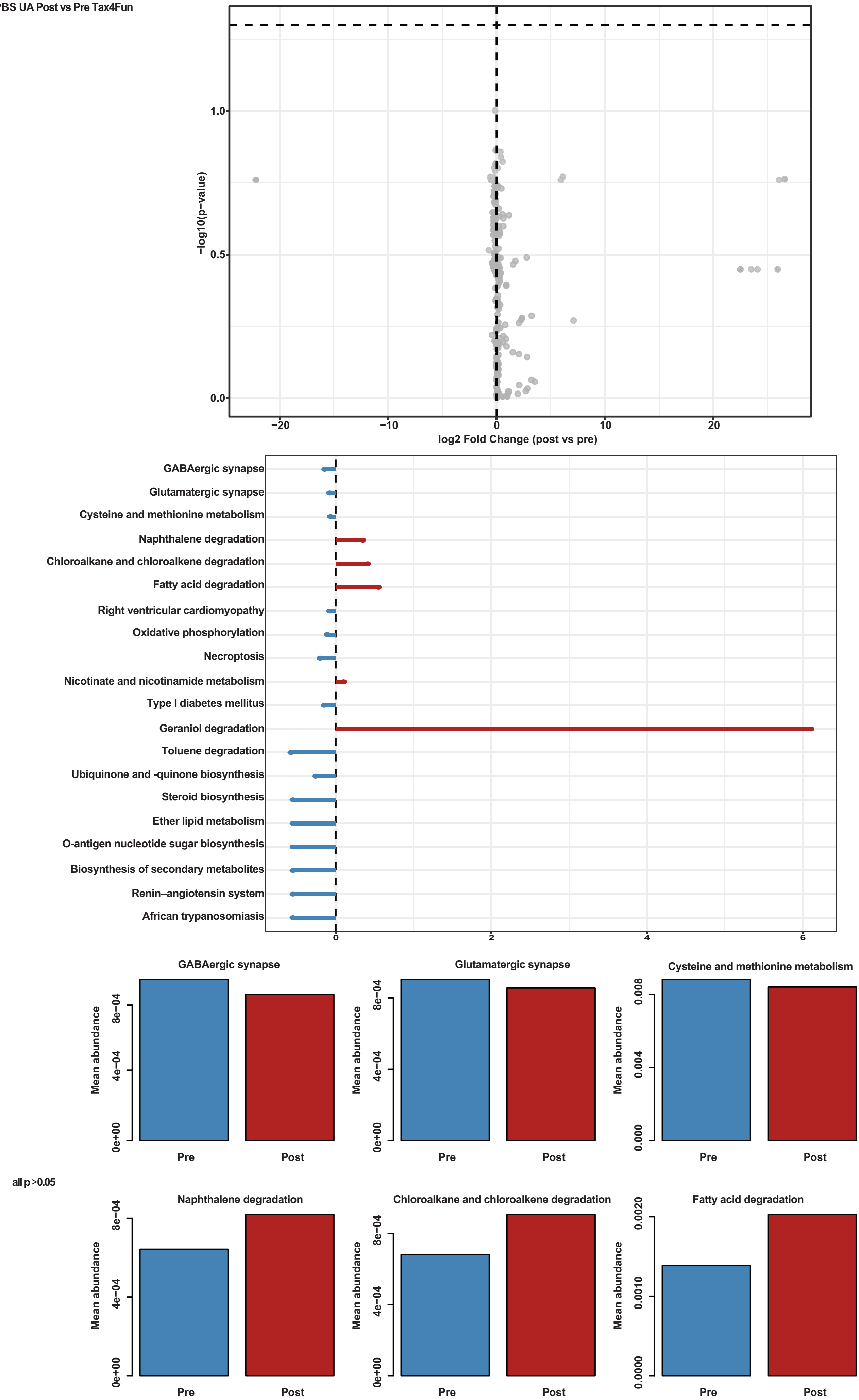

S. Fig. 10

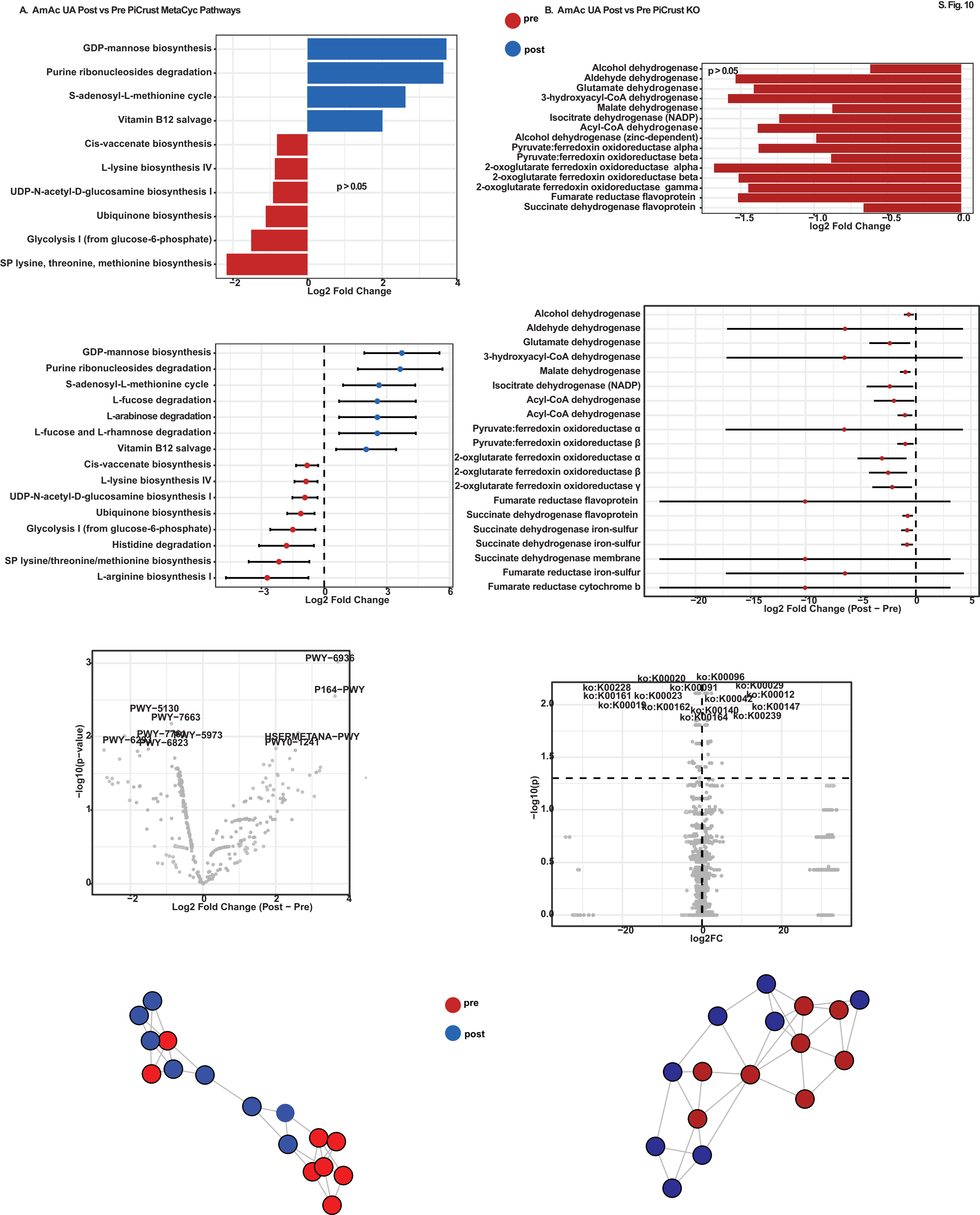
A. AmAc UA Post vs Pre PiCrust MetaCyc Pathways
GDP-mannose biosynthesis
Purine ribonucleosides degradation
S-adenosyl-L-methionine cycle
Vitamin B12 salvage
Cis-vaccenate biosynthesis
L-lysine biosynthesis IV
UDP-N-acetyl-D-glucosamine biosynthesis I
Ubiquinone biosynthesis
Glycolysis I (from glucose-6-phosphate)
SP lysine, threonine, methionine biosynthesis
p > 0.05
Log2 Fold Change
B. AmAc UA Post vs Pre PiCrust KO
pre
post
Alcohol dehydrogenase
Aldehyde dehydrogenase
Glutamate dehydrogenase
3-hydroxyacyl-CoA dehydrogenase
Malate dehydrogenase
Isocitrate dehydrogenase (NADP)
Acyl-CoA dehydrogenase
Alcohol dehydrogenase (zinc-dependent)
Pyruvate:ferredoxin oxidoreductase alpha
Pyruvate:ferredoxin oxidoreductase beta
2-oxoglutarate ferredoxin oxidoreductase alpha
2-oxoglutarate ferredoxin oxidoreductase beta
2-oxoglutarate ferredoxin oxidoreductase gamma
Fumarate reductase flavoprotein
Succinate dehydrogenase flavoprotein
p > 0.05
log2 Fold Change
GDP-mannose biosynthesis
Purine ribonucleosides degradation
S-adenosyl-L-methionine cycle
L-fucose degradation
L-arabinose degradation
L-fucose and L-rhamnose degradation
Vitamin B12 salvage
Cis-vaccenate biosynthesis
L-lysine biosynthesis IV
UDP-N-acetyl-D-glucosamine biosynthesis I
Ubiquinone biosynthesis
Glycolysis I (from glucose-6-phosphate)
Histidine degradation
SP lysine/threonine/methionine biosynthesis
L-arginine biosynthesis I
Log2 Fold Change
Alcohol dehydrogenase
Aldehyde dehydrogenase
Glutamate dehydrogenase
3-hydroxyacyl-CoA dehydrogenase
Malate dehydrogenase
Isocitrate dehydrogenase (NADP)
Acyl-CoA dehydrogenase
Acyl-CoA dehydrogenase
Pyruvate:ferredoxin oxidoreductase α
Pyruvate:ferredoxin oxidoreductase β
2-oxglutarate ferredoxin oxidoreductase α
2-oxglutarate ferredoxin oxidoreductase β
2-oxglutarate ferredoxin oxidoreductase γ
Fumarate reductase flavoprotein
Succinate dehydrogenase flavoprotein
Succinate dehydrogenase iron-sulfur
Succinate dehydrogenase iron-sulfur
Succinate dehydrogenase membrane
Fumarate reductase iron-sulfur
Fumarate reductase cytochrome b
log2 Fold Change (Post − Pre)
PWY−6936
P164−PWY
PWY−5130
PWY−7663
PWY−5973
PWY−6823
HSERMETANA−PWY
PWY0−1241
−log10(p−value)
Log2 Fold Change (Post − Pre)
ko:K00020
ko:K00096
ko:K00228
ko:K00091
ko:K00029
ko:K00161
ko:K00023
ko:K00012
ko:K00042
ko:K00019
ko:K00162
ko:K00147
ko:K00140
ko:K00239
ko:K00164
−log10(p)
log2FC
pre
post

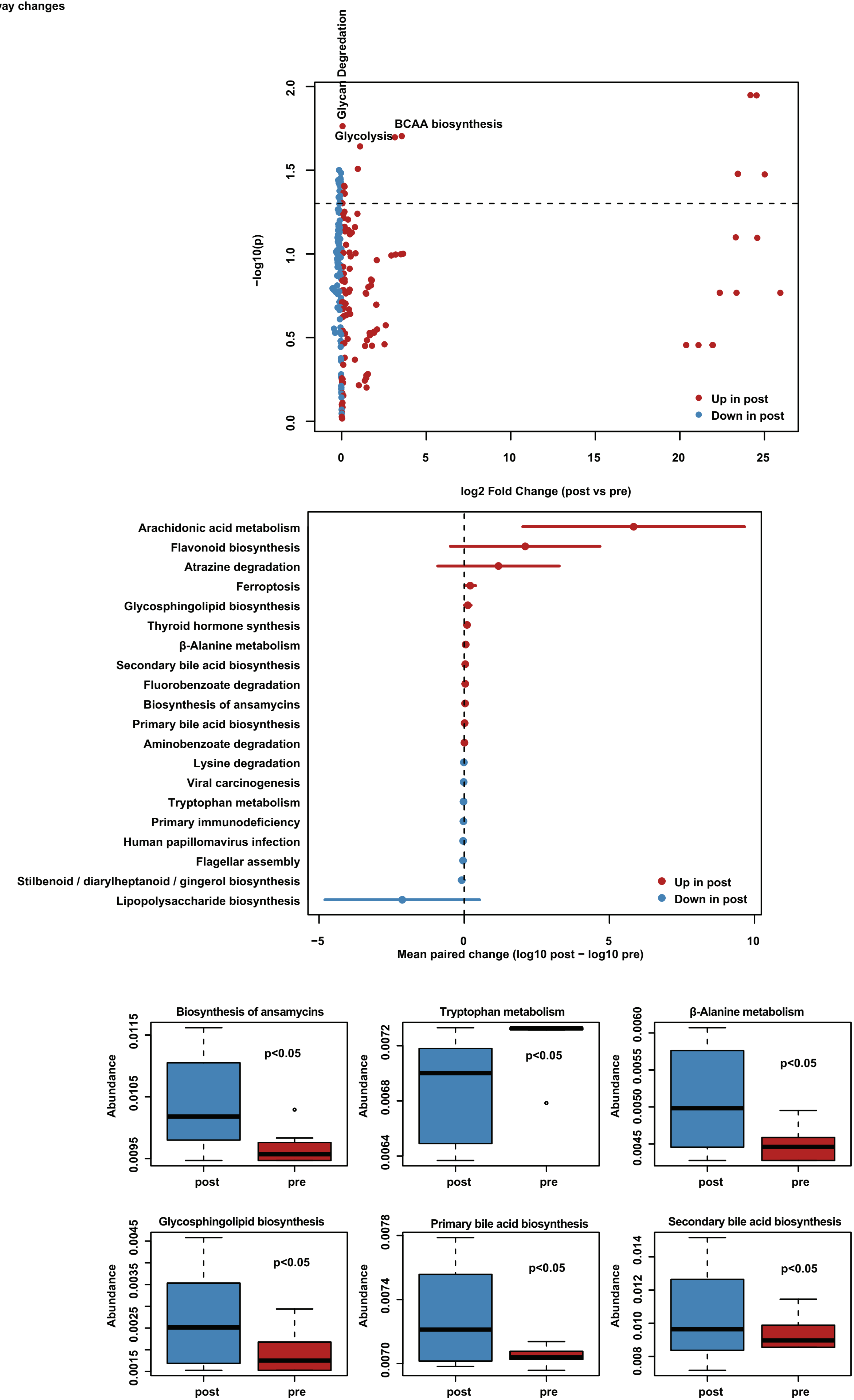
Glycan Degradation
BCAA biosynthesis
Glycolysis
−log10(p)
2.0
1.5
1.0
0.5
0.0
0
5
10
15
20
25
Up in post
Down in post
log2 Fold Change (post vs pre)
Arachidonic acid metabolism
Flavonoid biosynthesis
Atrazine degradation
Ferroptosis
Glycosphingolipid biosynthesis
Thyroid hormone synthesis
β-Alanine metabolism
Secondary bile acid biosynthesis
Fluorobenzoate degradation
Biosynthesis of ansamycins
Primary bile acid biosynthesis
Aminobenzoate degradation
Lysine degradation
Viral carcinogenesis
Tryptophan metabolism
Primary immunodeficiency
Human papillomavirus infection
Flagellar assembly
Stilbenoid / diarylheptanoid / gingerol biosynthesis
Lipopolysaccharide biosynthesis
Up in post
Down in post
−5
0
5
10
Mean paired change (log10 post − log10 pre)
Biosynthesis of ansamycins
Abundance
0.0095
0.0105
0.0115
p<0.05
post
pre
Tryptophan metabolism
Abundance
0.0064
0.0068
0.0072
p<0.05
post
pre
β-Alanine metabolism
Abundance
0.0045
0.0050
0.0055
0.0060
p<0.05
post
pre
Glycosphingolipid biosynthesis
Abundance
0.0015
0.0025
0.0035
0.0045
p<0.05
post
pre
Primary bile acid biosynthesis
Abundance
0.0070
0.0074
0.0078
p<0.05
post
pre
Secondary bile acid biosynthesis
Abundance
0.008
0.010
0.012
0.014
p<0.05
post
pre

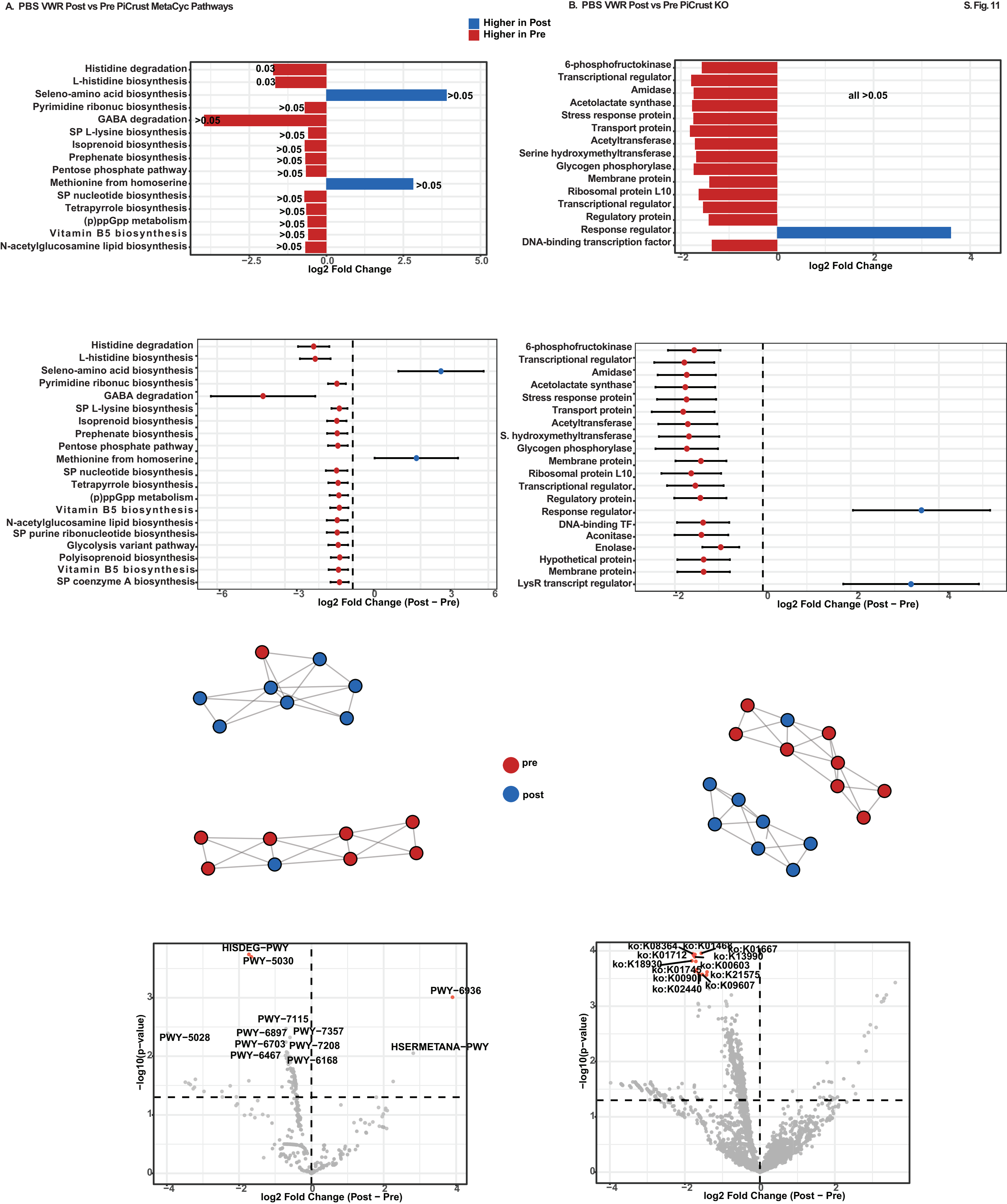
A. PBS VWR Post vs Pre PiCrust MetaCyc Pathways
B. PBS VWR Post vs Pre PiCrust KO
S. Fig. 11
Higher in Post
Higher in Pre
Histidine degradation
L-histidine biosynthesis
Seleno-amino acid biosynthesis
Pyrimidine ribonuc biosynthesis
GABA degradation
SP L-lysine biosynthesis
Isoprenoid biosynthesis
Prephenate biosynthesis
Pentose phosphate pathway
Methionine from homoserine
SP nucleotide biosynthesis
Tetrapyrrole biosynthesis
(p)ppGpp metabolism
Vitamin B5 biosynthesis
N-acetylglucosamine lipid biosynthesis
0.03
>0.05
log2 Fold Change
6-phosphofructokinase
Transcriptional regulator
Amidase
Acetolactate synthase
Stress response protein
Transport protein
Acetyltransferase
Serine hydroxymethyltransferase
Glycogen phosphorylase
Membrane protein
Ribosomal protein L10
Regulatory protein
Response regulator
DNA-binding transcription factor
all >0.05
SP purine ribonucleotide biosynthesis
Glycolysis variant pathway
Polyisoprenoid biosynthesis
SP coenzyme A biosynthesis
log2 Fold Change (Post − Pre)
S. hydroxymethyltransferase
DNA-binding TF
Aconitase
Enolase
Hypothetical protein
LysR transcript regulator
pre
post
HISDEG−PWY
PWY−5030
PWY−6936
PWY−7115
PWY−5028
PWY−6897
PWY−7357
PWY−6703
PWY−7208
PWY−6467
PWY−6168
HSERMETANA−PWY
−log10(p−value)
ko:K08364
ko:K01468
ko:K01667
ko:K01712
ko:K13990
ko:K18930
ko:K00603
ko:K01745
ko:K21575
ko:K00901
ko:K09607
ko:K02440

C. PBS VWR Post vs Pre Tax4Fun

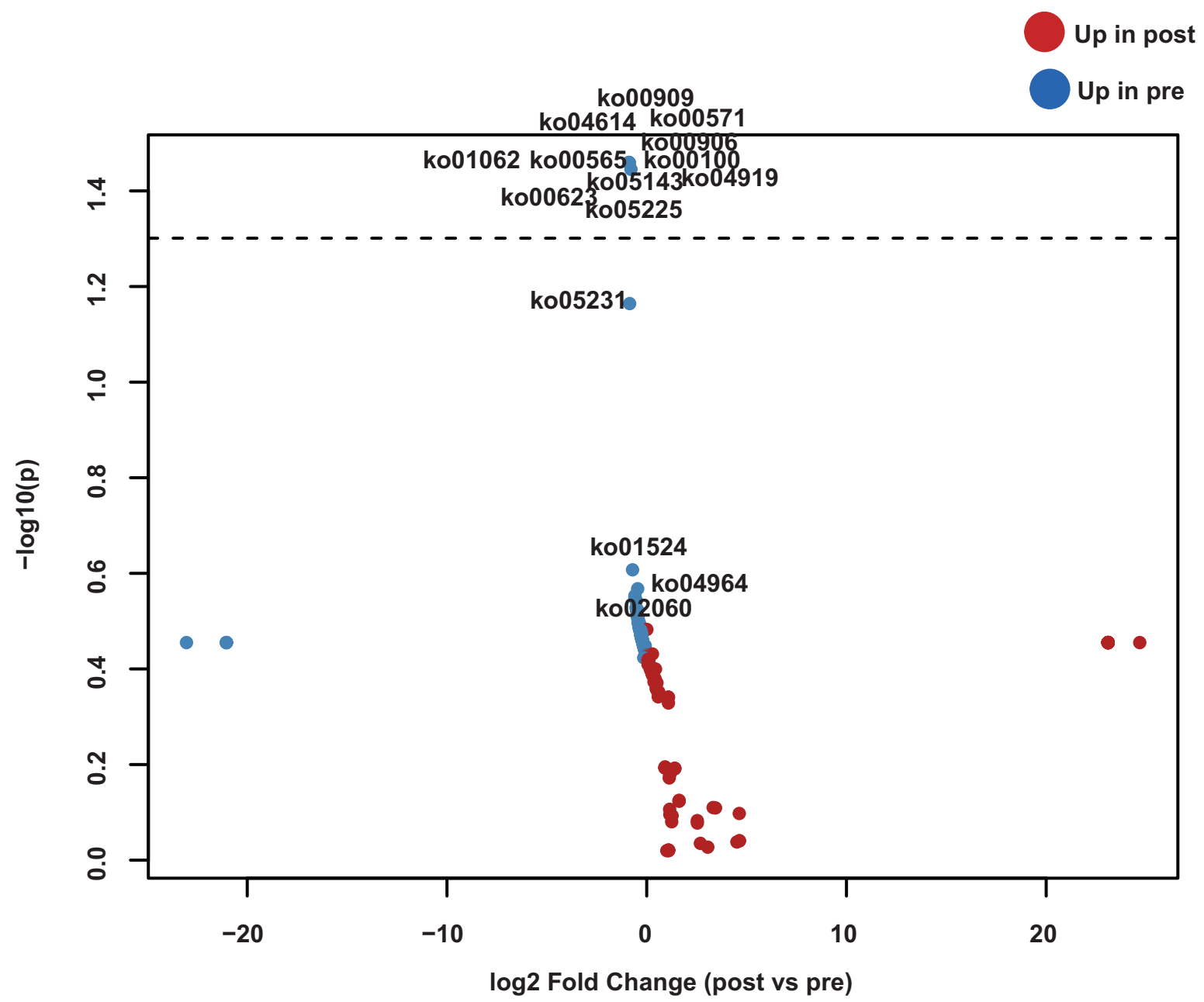

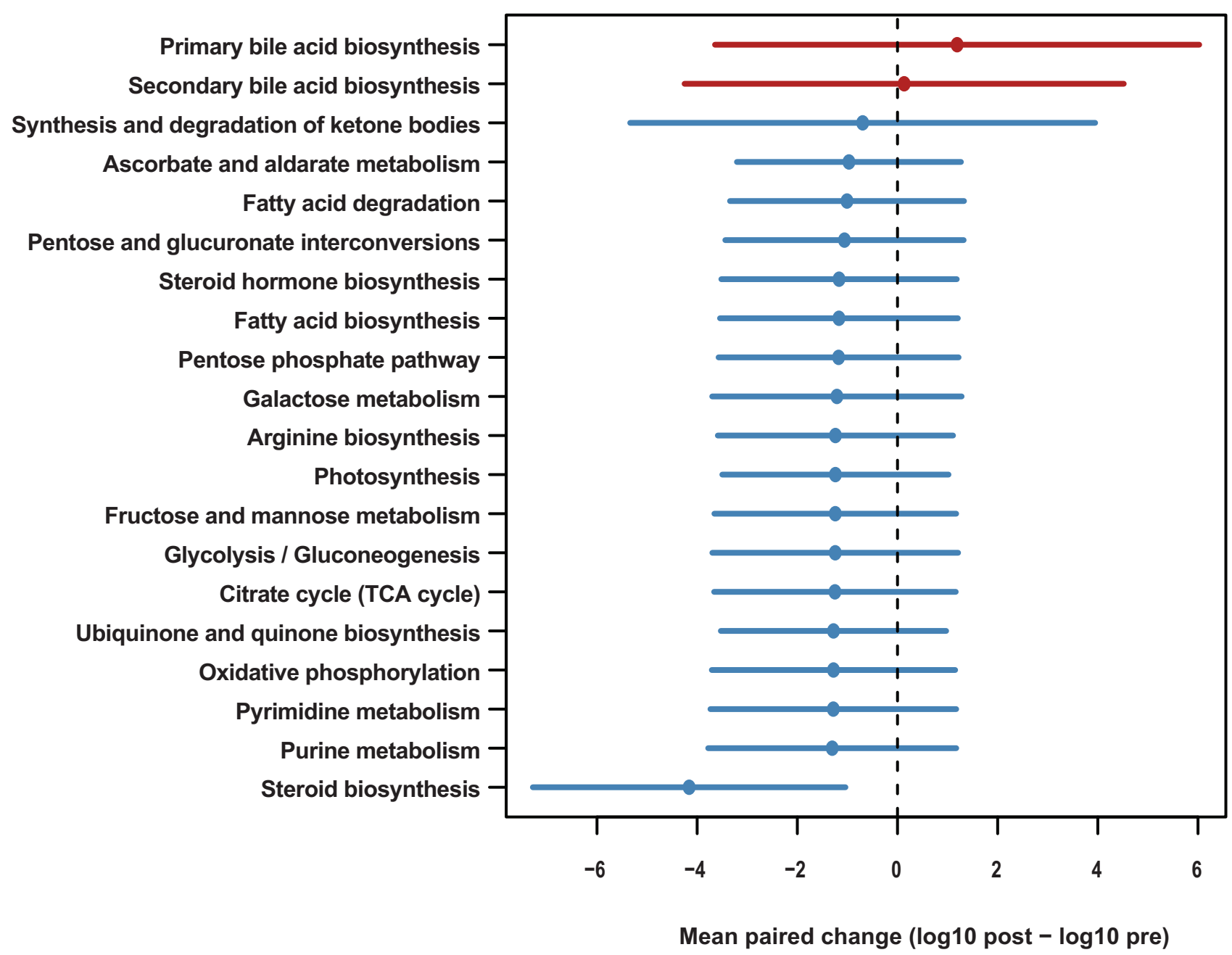

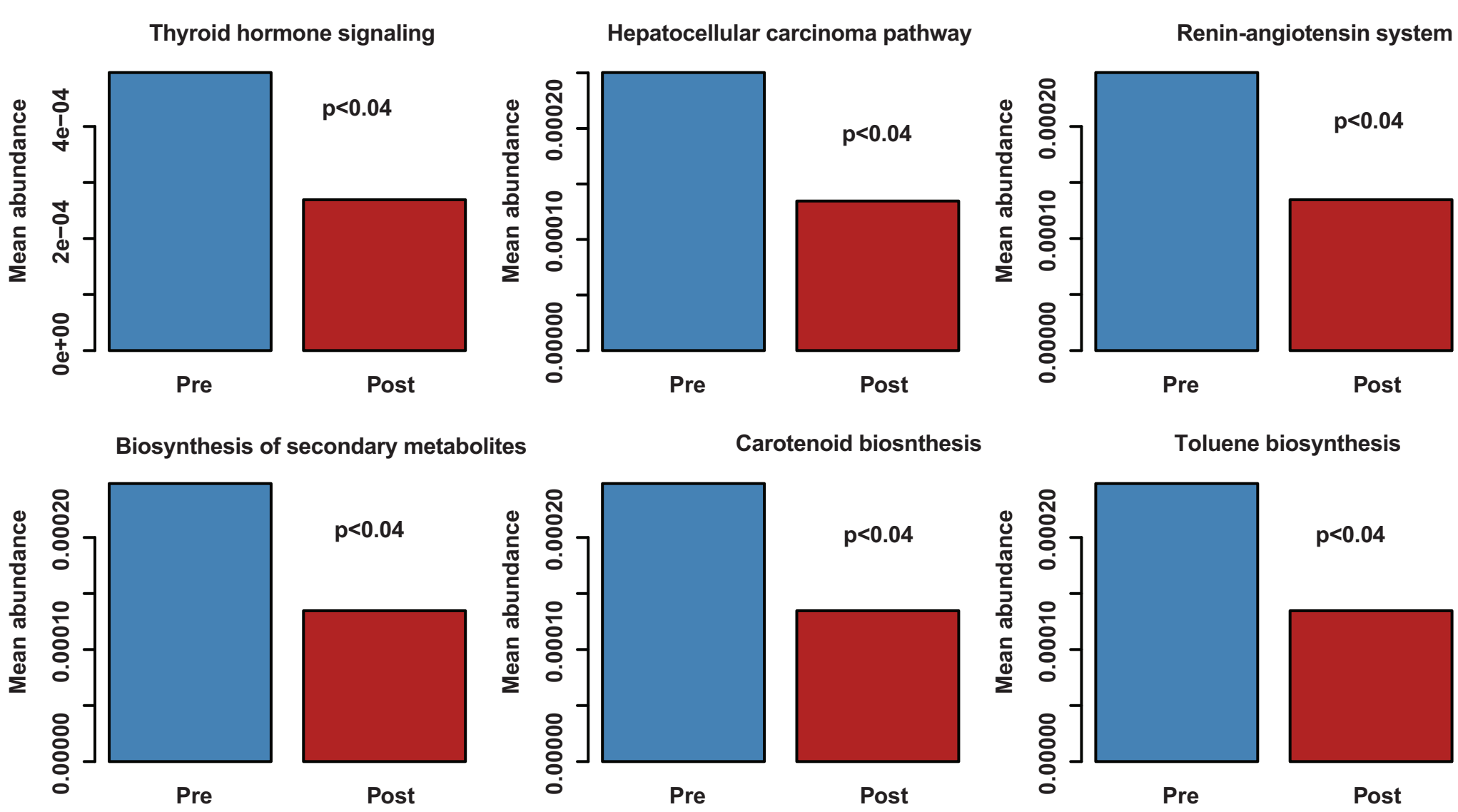

S. Fig. 12

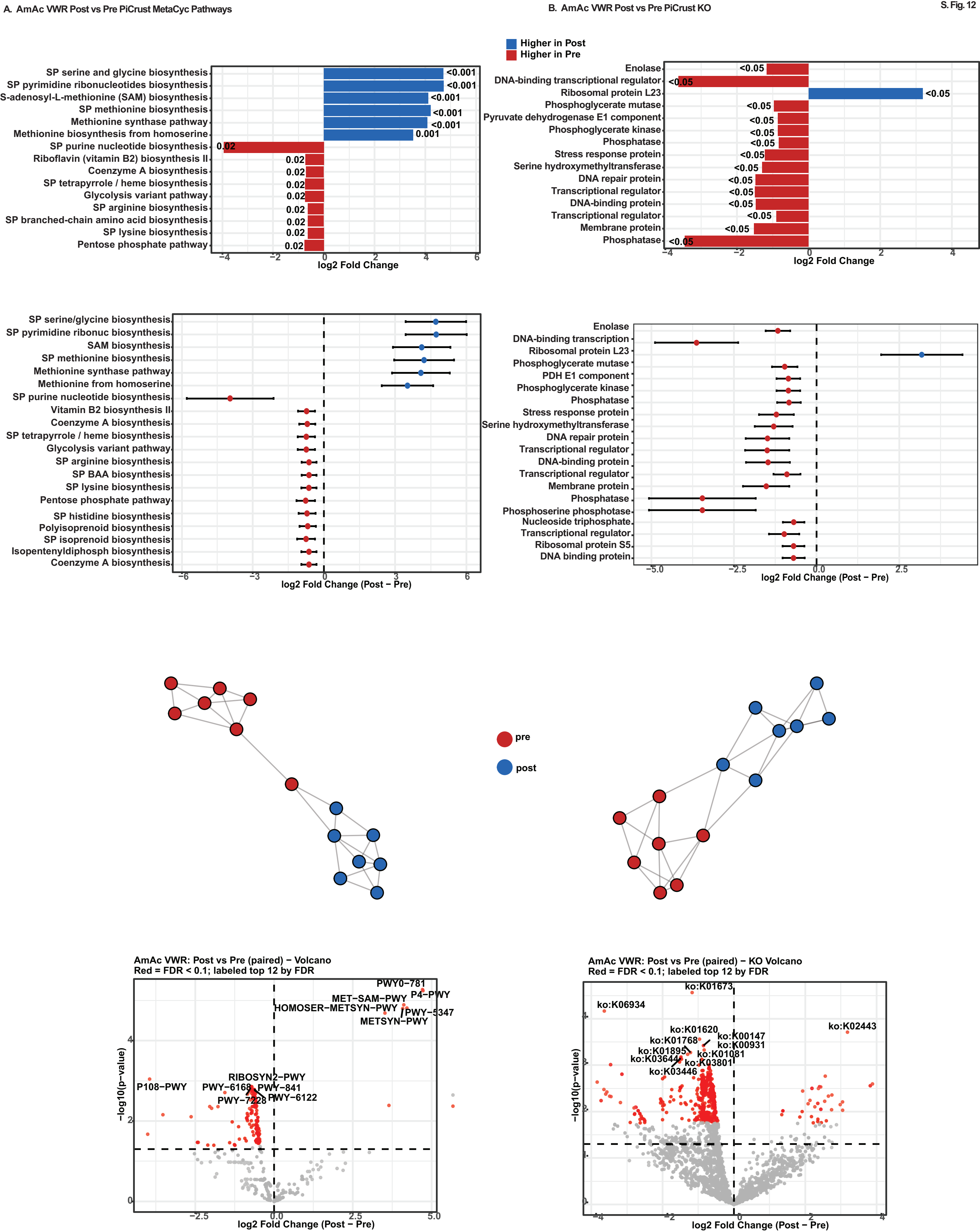

A. AmAc VWR Post vs Pre PiCrust MetaCyc Pathways
B. AmAc VWR Post vs Pre PiCrust KO
Higher in Post
Higher in Pre
SP serine and glycine biosynthesis
SP pyrimidine ribonucleotides biosynthesis
S-adenosyl-L-methionine (SAM) biosynthesis
SP methionine biosynthesis
Methionine synthase pathway
Methionine biosynthesis from homoserine
SP purine nucleotide biosynthesis
Riboflavin (vitamin B2) biosynthesis II
Coenzyme A biosynthesis
SP tetrapyrrole / heme biosynthesis
Glycolysis variant pathway
SP arginine biosynthesis
SP branched-chain amino acid biosynthesis
SP lysine biosynthesis
Pentose phosphate pathway
log2 Fold Change
Enolase
DNA-binding transcriptional regulator
Ribosomal protein L23
Phosphoglycerate mutase
Pyruvate dehydrogenase E1 component
Phosphoglycerate kinase
Phosphatase
Stress response protein
Serine hydroxymethyltransferase
DNA repair protein
Transcriptional regulator
DNA-binding protein
Membrane protein
SP serine/glycine biosynthesis
SP pyrimidine ribonuc biosynthesis
SAM biosynthesis
Methionine from homoserine
Vitamin B2 biosynthesis II
SP BAA biosynthesis
SP histidine biosynthesis
Polyisoprenoid biosynthesis
SP isoprenoid biosynthesis
Isopentenyldiphosph biosynthesis
log2 Fold Change (Post − Pre)
DNA-binding transcription
PDH E1 component
Phosphoserine phosphotase
Nucleoside triphosphate
Ribosomal protein S5
DNA binding protein
pre
post
AmAc VWR: Post vs Pre (paired) − Volcano
Red = FDR < 0.1; labeled top 12 by FDR
PWY0−781
P4−PWY
MET−SAM−PWY
HOMOSER−METSYN−PWY
PWY−5347
METSYN−PWY
RIBOSYN2−PWY
P108−PWY
PWY−6168
PWY−841
PWY−7228
PWY−6122
−log10(p−value)
AmAc VWR: Post vs Pre (paired) − KO Volcano
ko:K01673
ko:K06934
ko:K01620
ko:K01768
ko:K00147
ko:K00931
ko:K01895
ko:K01081
ko:K03644
ko:K03801
ko:K03446
ko:K02443

C. AmAc VWR Post vs Pre Tax4Fun

S. Fig. 12

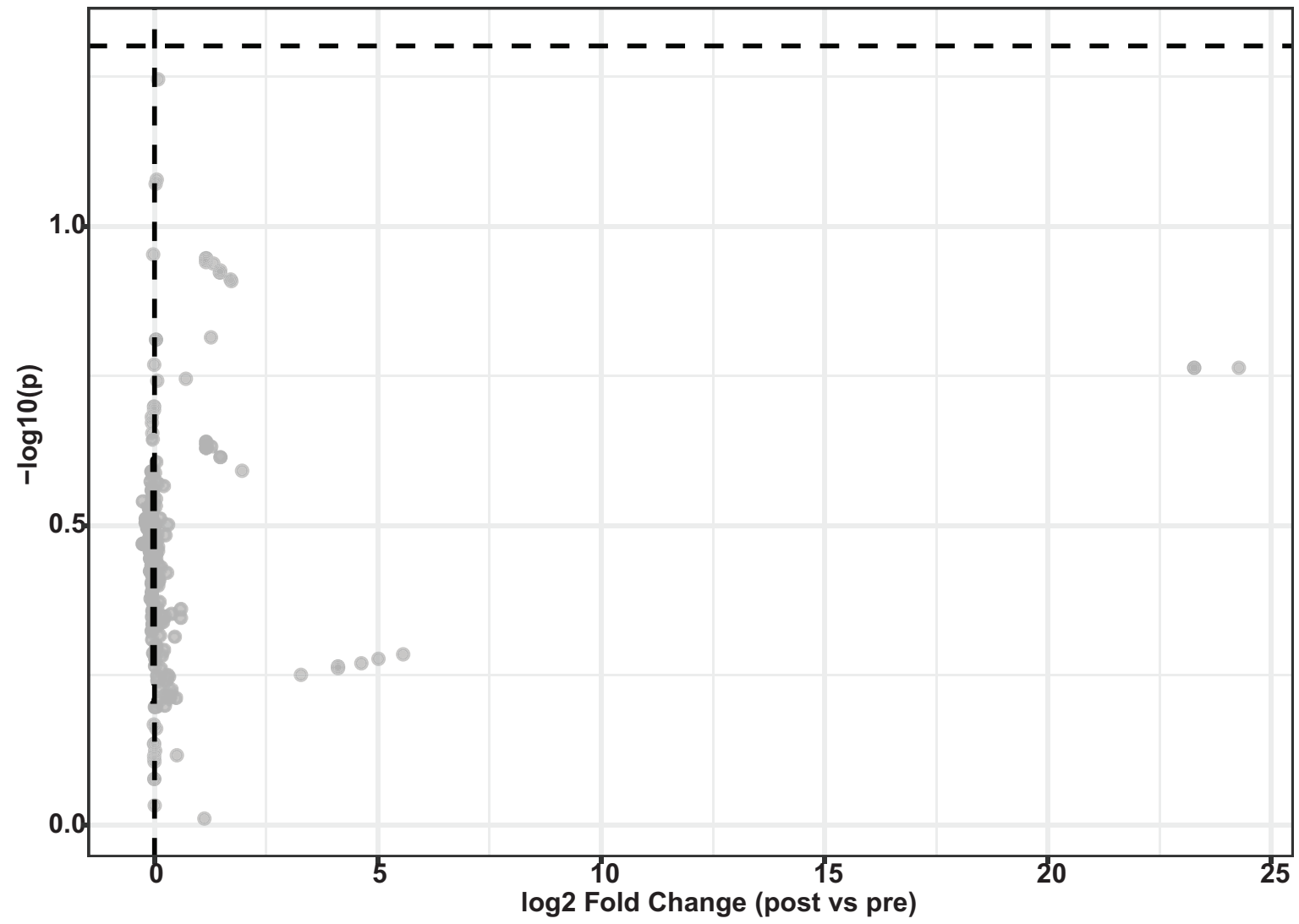

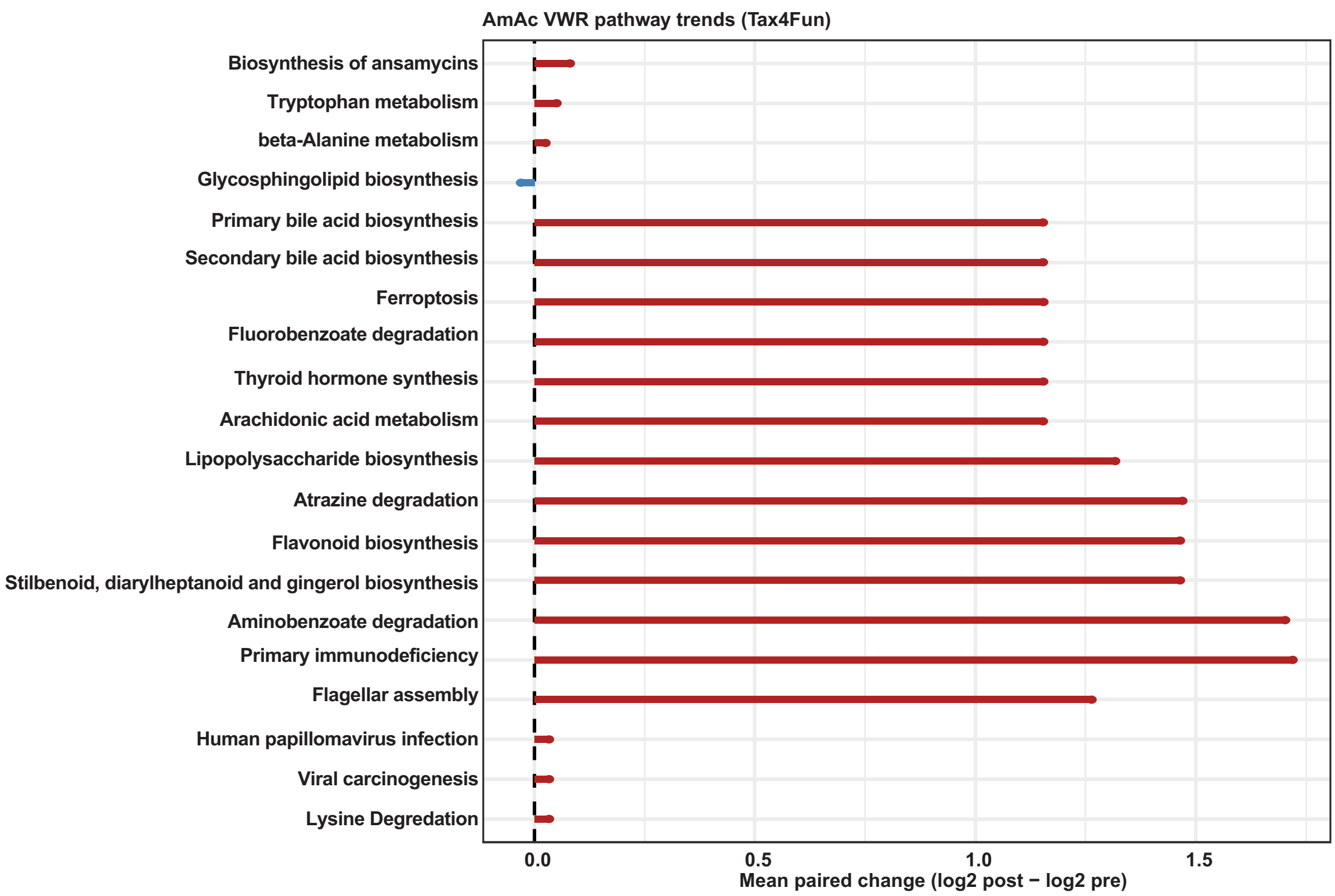

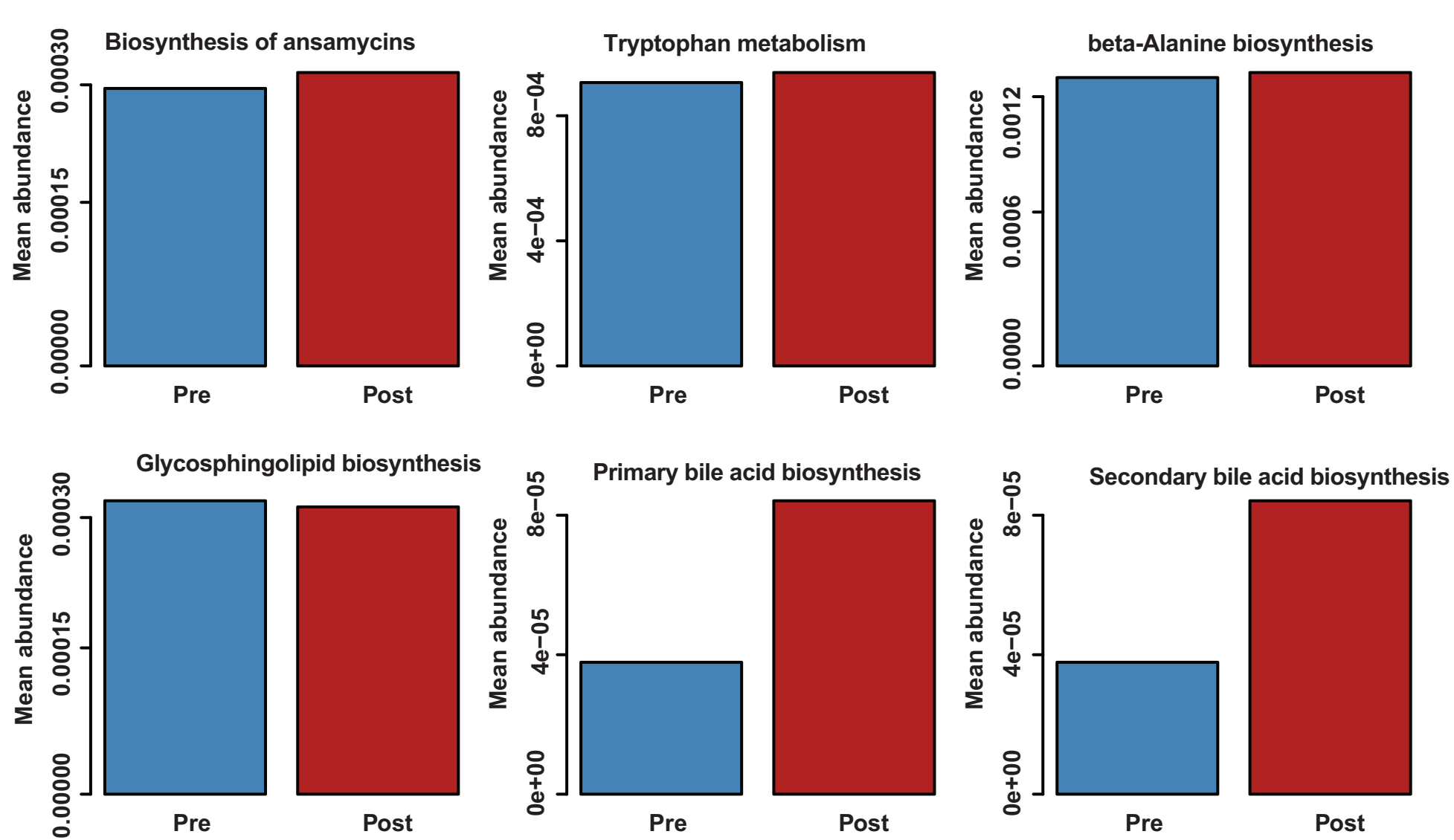

S. Fig. 13

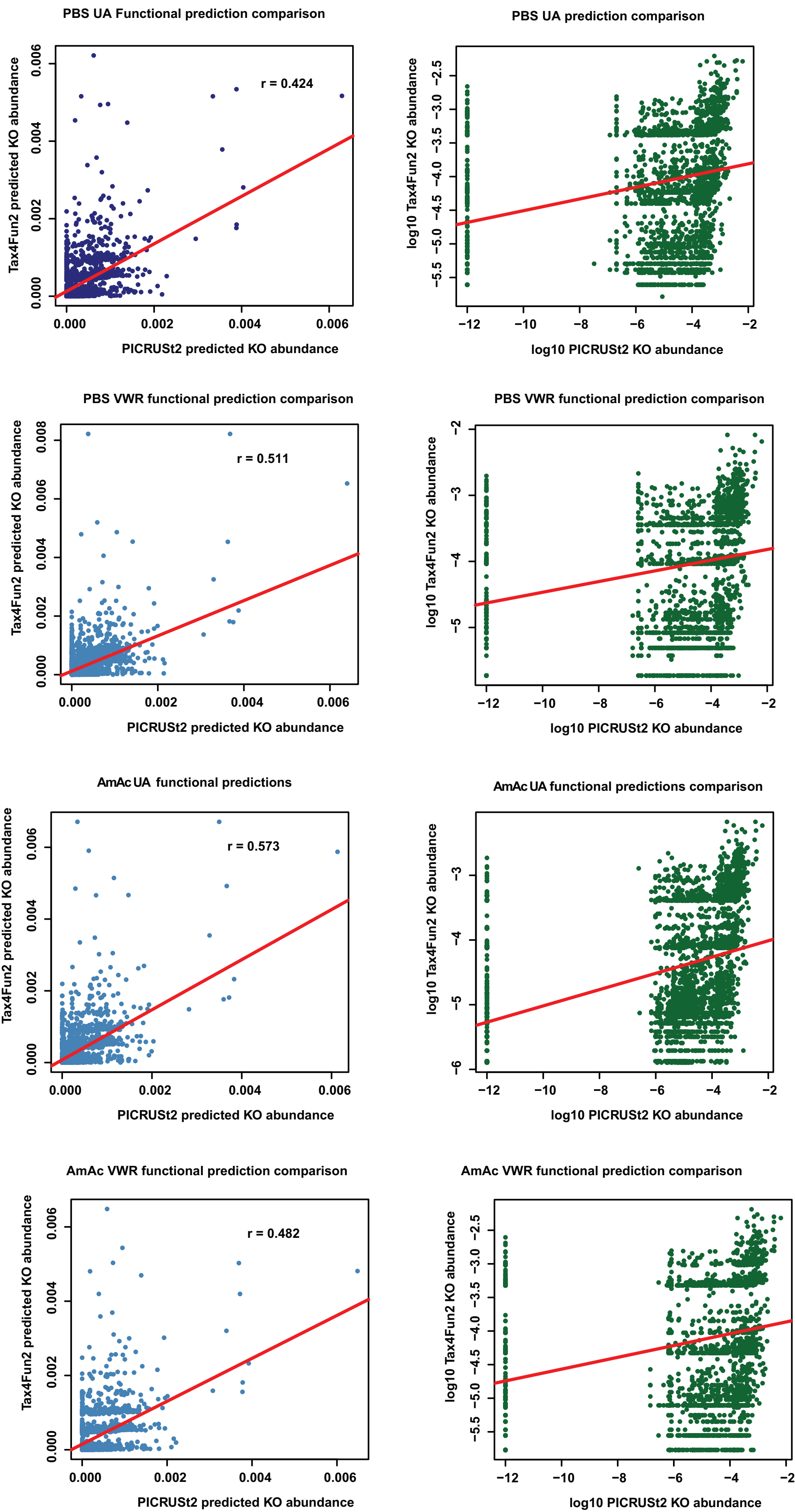

PBS UA Functional prediction comparison
r = 0.424
Tax4Fun2 predicted KO abundance
PICRUSt2 predicted KO abundance
0.000
0.002
0.004
0.006
PBS UA prediction comparison
log10 Tax4Fun2 KO abundance
log10 PICRUSt2 KO abundance
−2.5
−3.0
−3.5
−4.0
−4.5
−5.0
−5.5
−12
−10
−8
−6
−4
−2
PBS VWR functional prediction comparison
r = 0.511
Tax4Fun2 predicted KO abundance
PICRUSt2 predicted KO abundance
0.000
0.002
0.004
0.006
0.008
PBS VWR functional prediction comparison
log10 Tax4Fun2 KO abundance
log10 PICRUSt2 KO abundance
−2
−3
−4
−5
AmAc UA functional predictions
r = 0.573
Tax4Fun2 predicted KO abundance
PICRUSt2 predicted KO abundance
AmAc UA functional predictions comparison
log10 Tax4Fun2 KO abundance
log10 PICRUSt2 KO abundance
−6
AmAc VWR functional prediction comparison
r = 0.482
Tax4Fun2 predicted KO abundance
PICRUSt2 predicted KO abundance
AmAc VWR functional prediction comparison
log10 Tax4Fun2 KO abundance
log10 PICRUSt2 KO abundance

S. Fig. 14

A. PiCrust MetaCyc Pathways

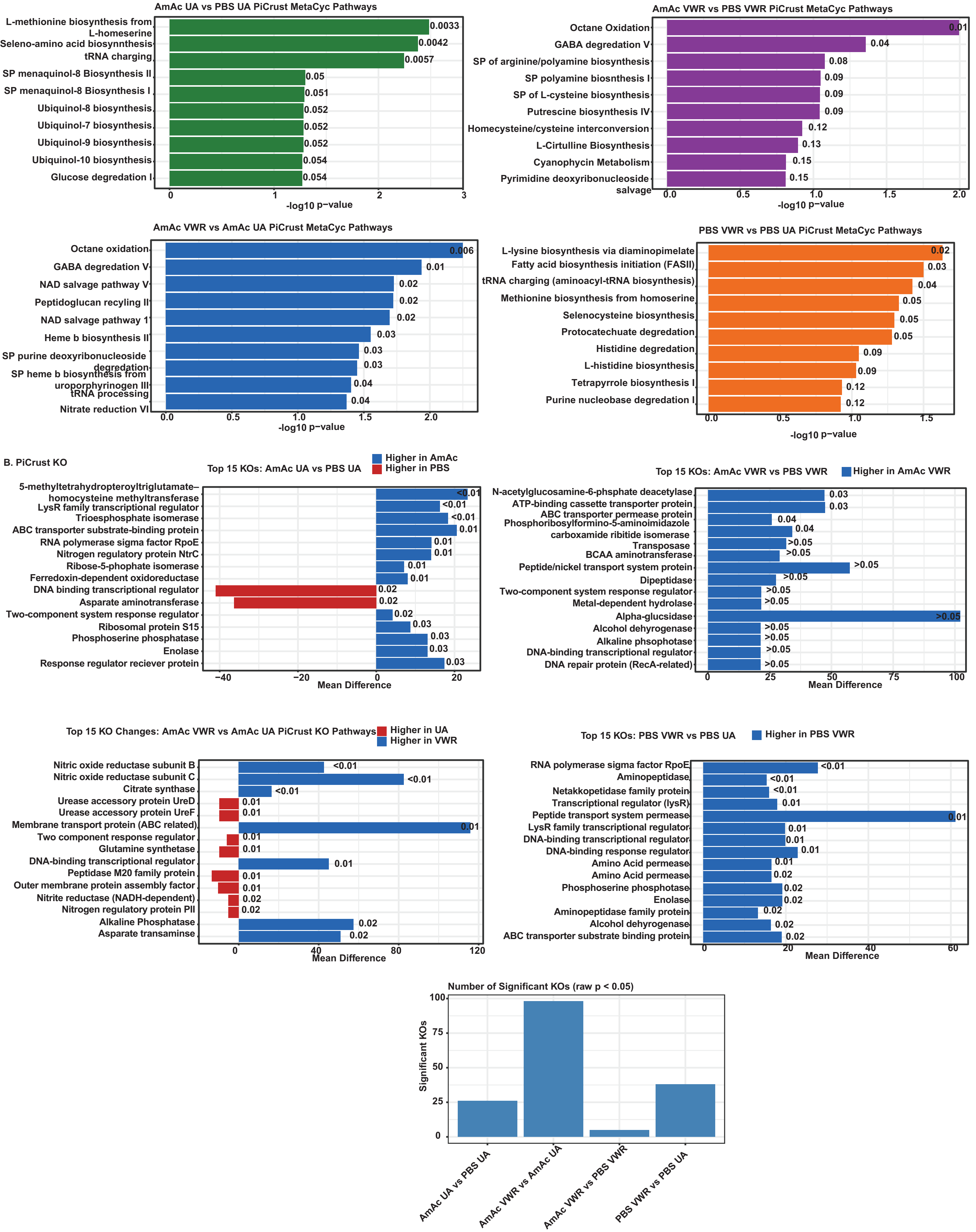

S. Fig. 15

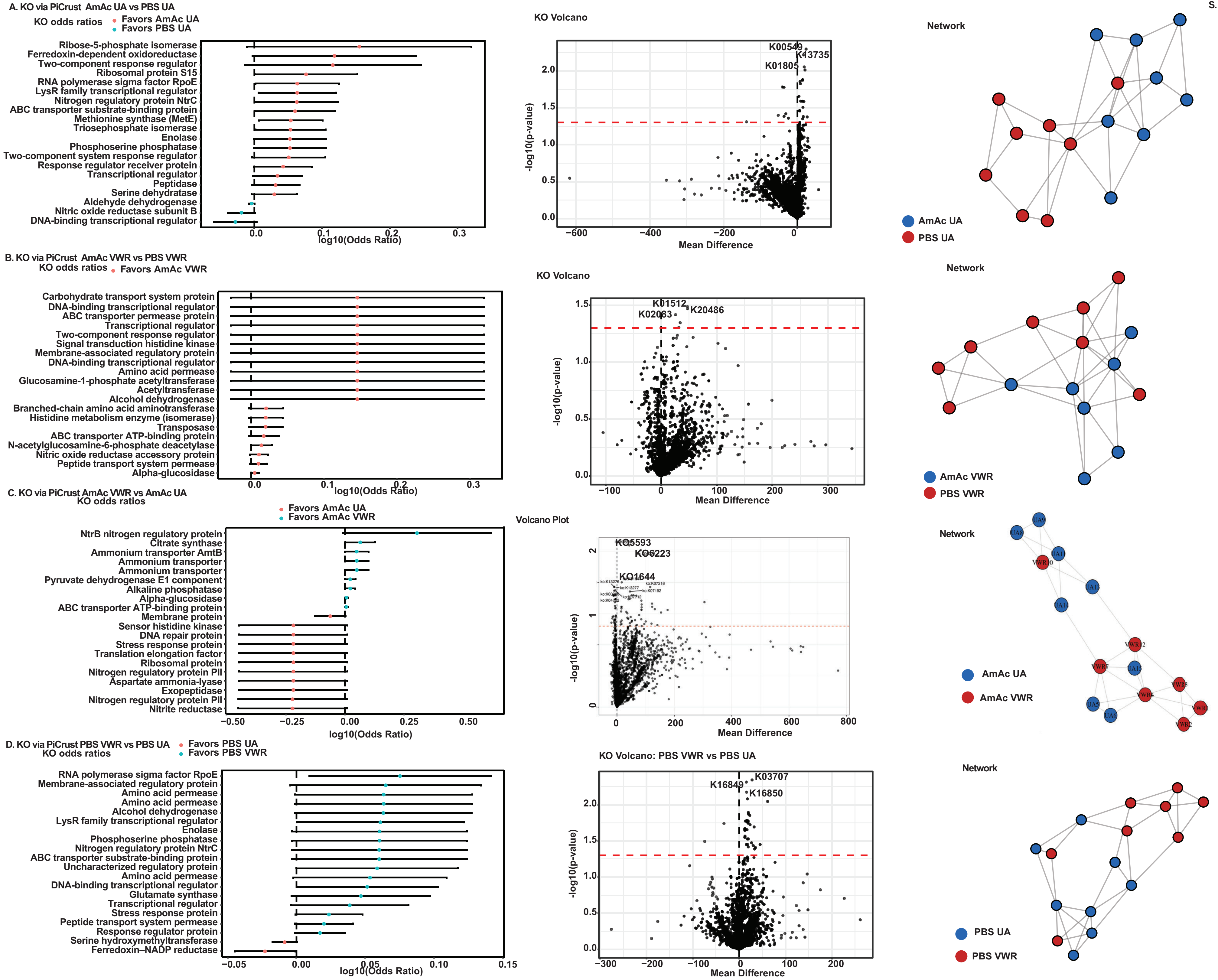

Dots = OR, bars = 95% CI (top 20 by raw p)

A. AmAcUA vs PBSUA

Tax4Fun KO forest plot:

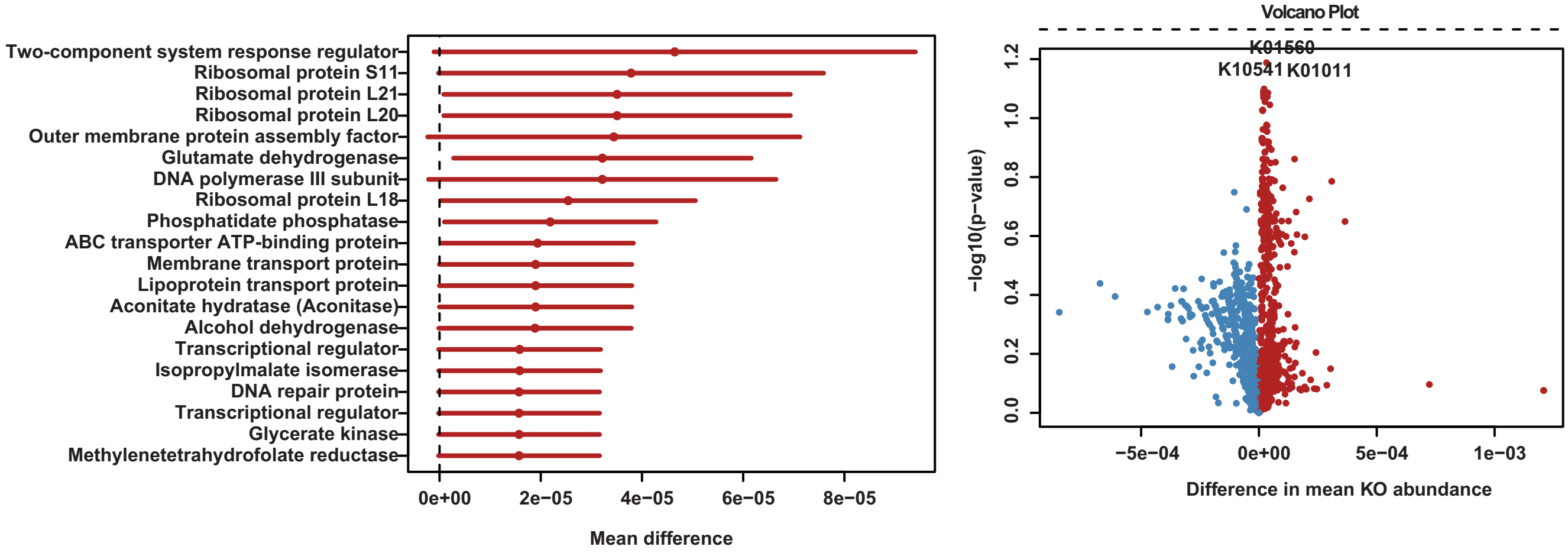

p>0.05

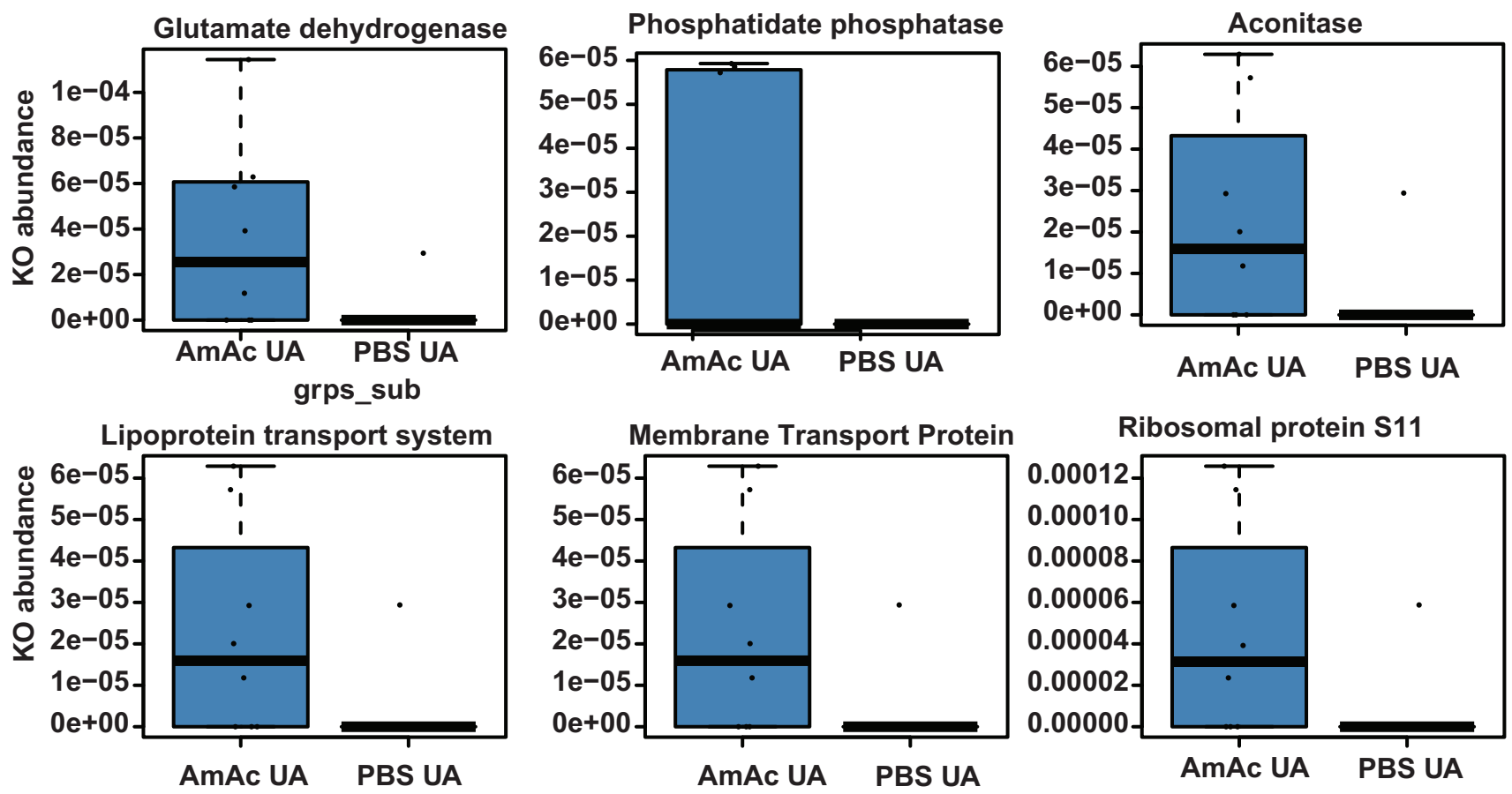

B. AmAc VWR vs PBS VWR

Tax4Fun KO forest plot:

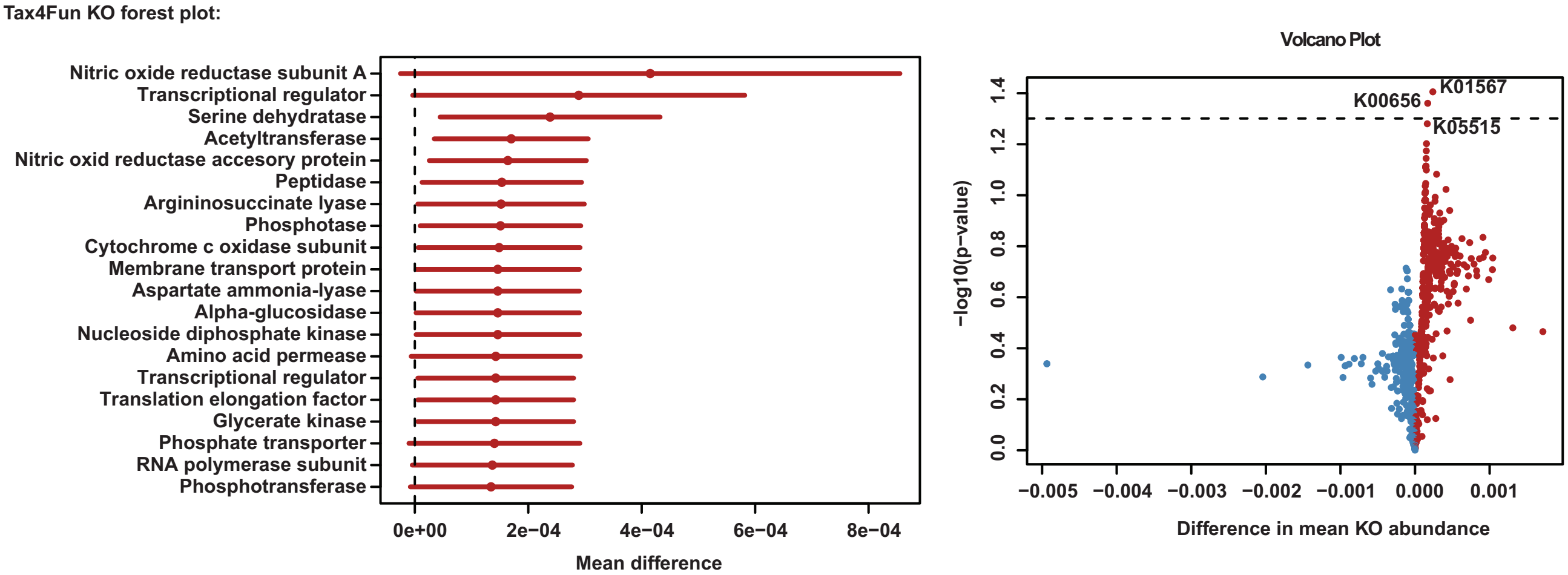

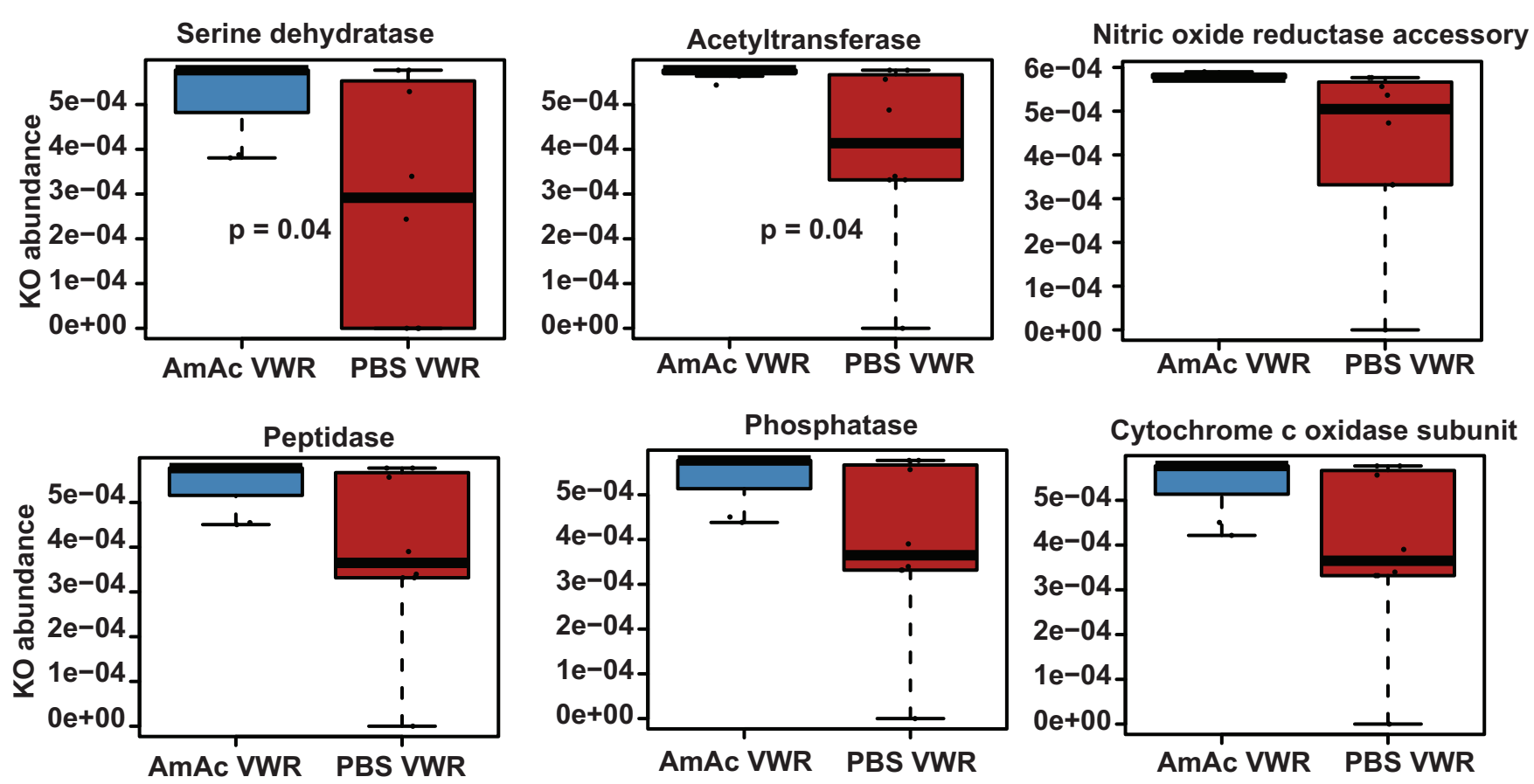

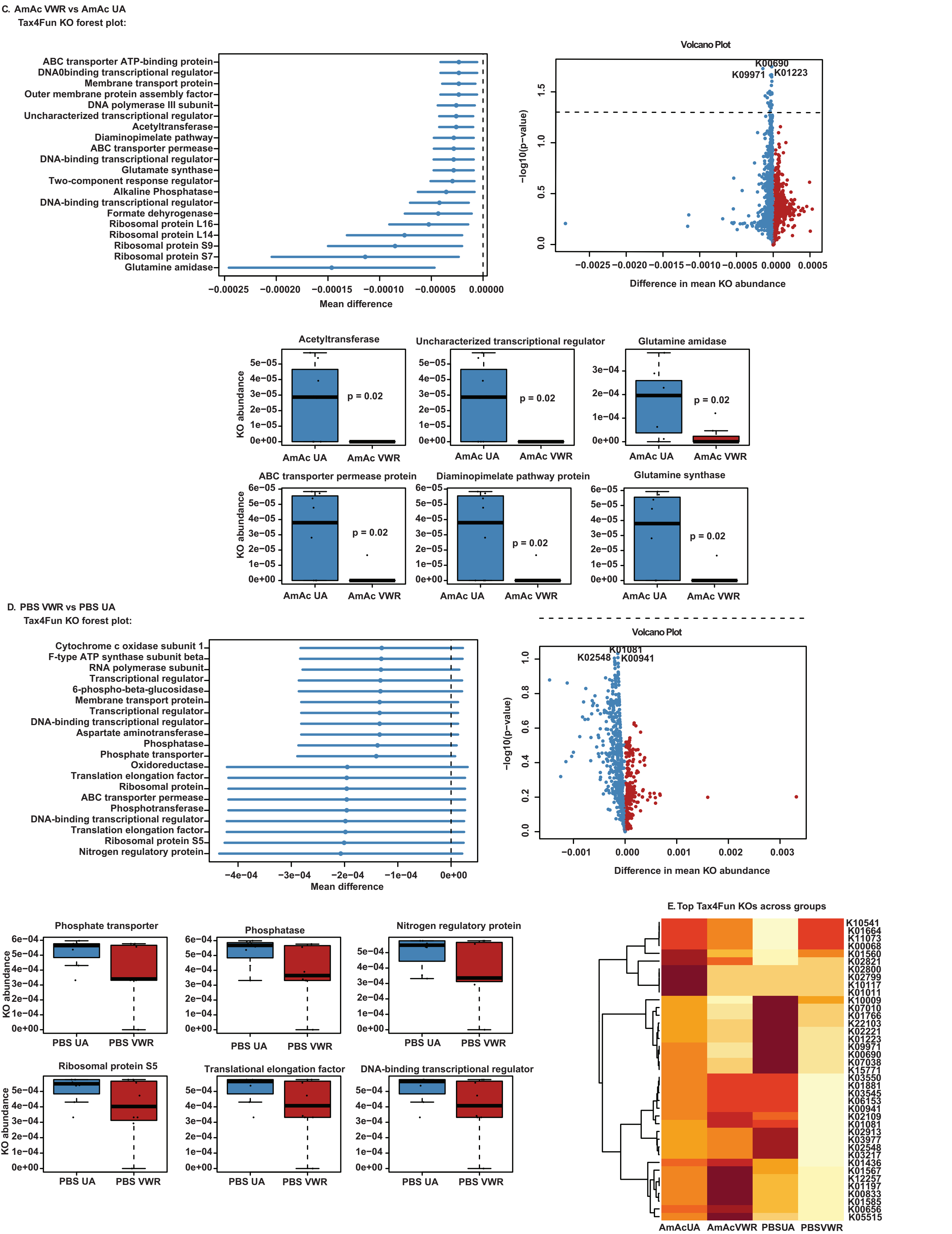

C. AmAc VWR vs AmAc UA
Tax4Fun KO forest plot:
ABC transporter ATP-binding protein
DNA0binding transcriptional regulator
Membrane transport protein
Outer membrane protein assembly factor
DNA polymerase III subunit
Uncharacterized transcriptional regulator
Acetyltransferase
Diaminopimelate pathway
ABC transporter permease
DNA-binding transcriptional regulator
Glutamate synthase
Two-component response regulator
Alkaline Phosphatase
DNA-binding transcriptional regulator
Formate dehyrogenase
Ribosomal protein L16
Ribosomal protein L14
Ribosomal protein S9
Ribosomal protein S7
Glutamine amidase
Mean difference
Volcano Plot
K00690
K09971
K01223
−log10(p−value)
Difference in mean KO abundance
Acetyltransferase
Uncharacterized transcriptional regulator
Glutamine amidase
ABC transporter permease protein
Diaminopimelate pathway protein
Glutamine synthase
KO abundance
p = 0.02
AmAc UA
AmAc VWR
D. PBS VWR vs PBS UA
Tax4Fun KO forest plot:
Cytochrome c oxidase subunit 1
F-type ATP synthase subunit beta
RNA polymerase subunit
Transcriptional regulator
6-phospho-beta-glucosidase
Membrane transport protein
Transcriptional regulator
DNA-binding transcriptional regulator
Aspartate aminotransferase
Phosphatase
Phosphate transporter
Oxidoreductase
Translation elongation factor
Ribosomal protein
ABC transporter permease
Phosphotransferase
DNA-binding transcriptional regulator
Translation elongation factor
Ribosomal protein S5
Nitrogen regulatory protein
Mean difference
Volcano Plot
K01081
K02548
K00941
−log10(p−value)
Difference in mean KO abundance
Phosphate transporter
Phosphatase
Nitrogen regulatory protein
Ribosomal protein S5
Translational elongation factor
DNA-binding transcriptional regulator
KO abundance
PBS UA
PBS VWR
E. Top Tax4Fun KOs across groups
K10541
K01664
K11073
K00068
K01560
K02821
K02800
K02799
K10117
K01011
K10009
K07010
K01766
K22103
K02221
K01223
K09971
K00690
K07038
K15771
K03550
K01881
K03545
K06153
K00941
K02109
K01081
K02913
K03977
K02548
K03217
K01436
K01567
K12257
K01197
K00833
K01585
K00656
K05515
AmAcUA
AmAcVWR
PBSUA
PBSVWR

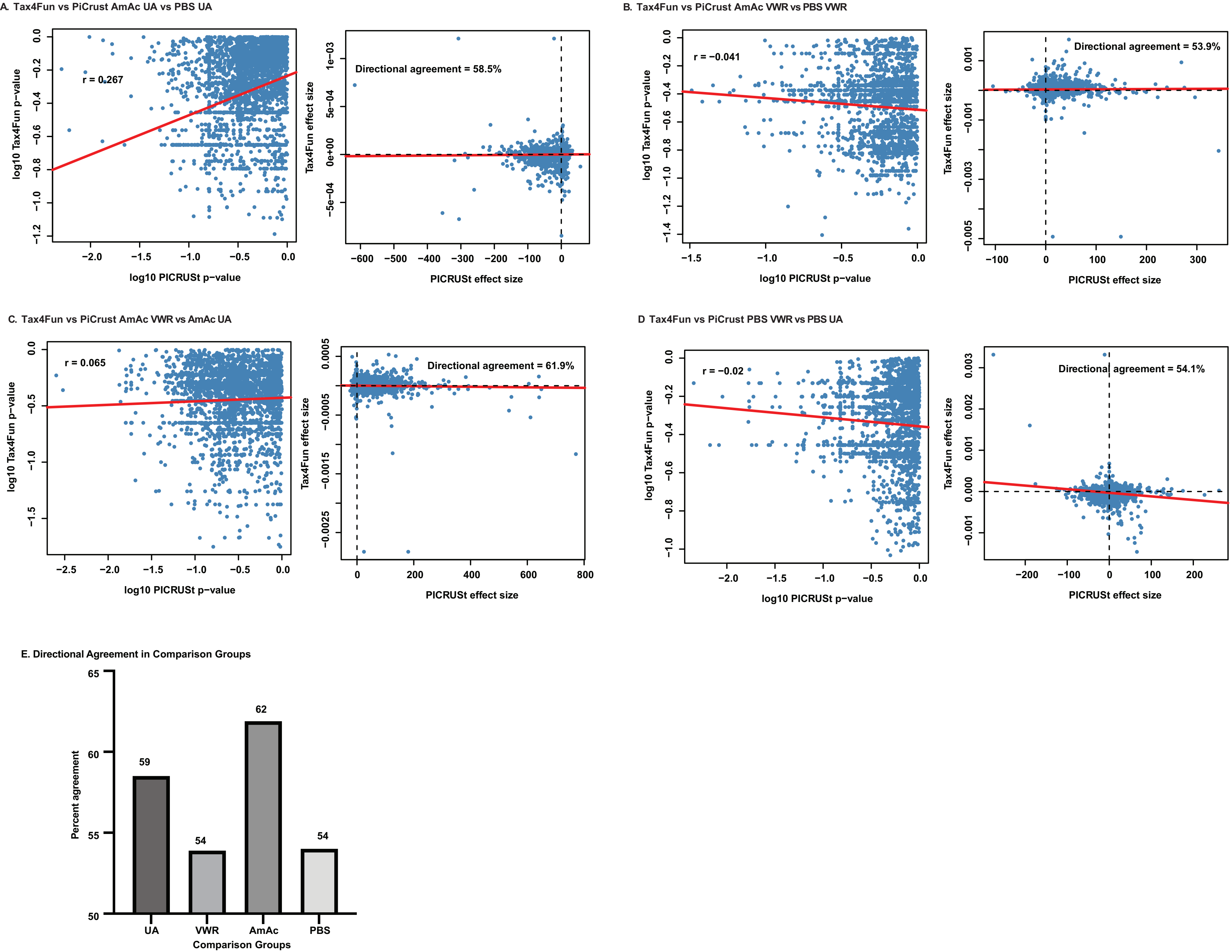
A. Tax4Fun vs PiCrust AmAc UA vs PBS UA
r = 0.267
log10 Tax4Fun p−value
log10 PICRUSt p−value
Directional agreement = 58.5%
Tax4Fun effect size
PICRUSt effect size
B. Tax4Fun vs PiCrust AmAc VWR vs PBS VWR
r = −0.041
log10 Tax4Fun p−value
log10 PICRUSt p−value
Directional agreement = 53.9%
Tax4Fun effect size
PICRUSt effect size
C. Tax4Fun vs PiCrust AmAc VWR vs AmAc UA
r = 0.065
log10 Tax4Fun p−value
log10 PICRUSt p−value
Directional agreement = 61.9%
Tax4Fun effect size
PICRUSt effect size
D Tax4Fun vs PiCrust PBS VWR vs PBS UA
r = −0.02
log10 Tax4Fun p−value
log10 PICRUSt p−value
Directional agreement = 54.1%
Tax4Fun effect size
PICRUSt effect size
E. Directional Agreement in Comparison Groups
Percent agreement
59
54
62
54
UA
VWR
AmAc
PBS
Comparison Groups

S. Fig. 18

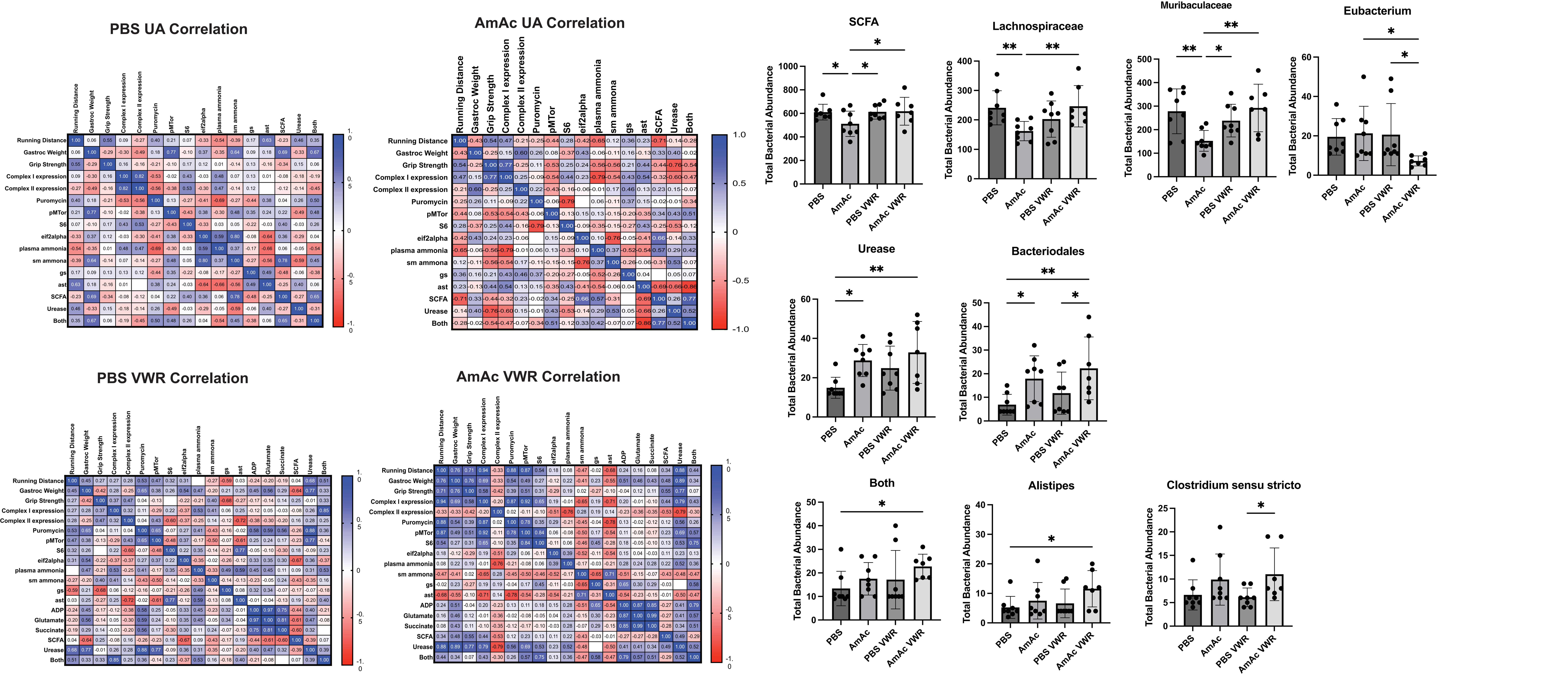
PBS UA Correlation
AmAc UA Correlation
PBS VWR Correlation
AmAc VWR Correlation
Running Distance
Gastroc Weight
Grip Strength
Complex I expression
Complex II expression
Puromycin
pMTor
S6
eif2alpha
plasma ammonia
sm ammonia
gs
ast
ADP
Glutamate
Succinate
SCFA
Urease
Both
SCFA
Lachnospiraceae
Muribaculaceae
Eubacterium
Urease
Bacteriodales
Both
Alistipes
Clostridium sensu stricto
Total Bacterial Abundance
PBS
AmAc
PBS VWR
AmAc VWR
