# Supplementary material for: Voluntary wheel running modulates murine gut microbiome during hyperammonemic stress
Source: Microbiol Spectr. 2026 Jul 15;14(8):e03194-25. doi: 10.1128/spectrum.03194-25 (PMC13435939; doi:10.1128/spectrum.03194-25)
Supplement: Supplementary file 2 [file spectrum.03194-25-s0001.docx]

**Supplementary Figure Legends.** Supplementary figures provide extended analyses of microbial composition, co-abundance networks, and predicted functional pathways**.**

**Supplementary Figure 1.** **Baseline microbiome composition and diversity prior to any treatment or intervention.** Male C57BL6/J mice aged 8–10 weeks were treated with either PBS or 2.5 mmol·kg⁻¹·d⁻¹ ammonium acetate (AmAc) for 42 days (treatment period). Two weeks after the osmotic pumps were installed, mice were randomized to usual activity (UA) or voluntary wheel running (VWR) for 4 weeks (intervention period). Fecal samples collected at baseline (prior to any intervention or treatment) were analyzed for diversity and taxonomic composition. **A.** Shannon alpha diversity plotted by group at baseline, showing similar within-group diversity across all four treatment arms. **B.** Constrained correspondence analysis (CCA)plot of genus-level beta diversity PERMANOVA R² = 0.14, p = 0.43. **C.** Heatmap showing genus-level percent read abundance across individual baseline samples. **D.** Heatmap showing raw genus-level relative abundance. **E.** Alluvial plots showing genus-level abundance at baseline for each group. **F.** Venn diagram of shared and unique genera between treatment groups. **G.** Bar graphs showing differential abundance of top genera at baseline. **Data:** mean ± SD. **Statistical analyses: White’s non-parametric test with pooled variance and bootstrapped p-values** was used for group comparisons in **Panels A and G. PERMANOVA** was used for beta diversity analysis in **Panel B**. p < 0.05; **p** < 0.01; ***p*** < 0.001. n = 8 mice per group.

**Supplementary Figure 2.** **Correlation difference matrices at baseline across treatment groups.**
Male C57BL6/J mice aged 8–10 weeks were treated with either PBS or 2.5 mmol·kg⁻¹·d⁻¹ ammonium acetate (AmAc) for 42 days (treatment period). Two weeks after the osmotic pumps were installed, mice were randomized to usual activity (UA) or voluntary wheel running (VWR) for 4 weeks (intervention period). Fecal samples collected at baseline were analyzed for diversity and taxonomic composition. Amplicon sequence variance (ASV)-level correlation networks were compared pairwise between treatment groups at baseline to assess similarity in microbial co-abundance structure. **A.** AmAc-UA vs. PBS-UA; **B.** PBS-VWR vs. PBS-UA. **C.** AmAc-VWR vs. PBS-VWR. **D.** AmAc-VWR vs. AmAc-UA. Each panel includes a Pearson correlation scatterplot showing the relationship between mean ASV abundances across the two groups being compared at baseline. These comparisons reflect the consistency or divergence in microbial community structure between treatment arms at baseline. **Statistical analyses:** Pearson correlation was used to assess similarity in mean ASV abundance between groups at baseline and to construct ASV–ASV co-abundance networks. ASV-ASV correlations were computed using **Pearson correlation coefficients.** Scatterplots display the strength and direction of correlation for shared ASVs, along with the corresponding r and p-values. n = 8 samples per group.

**Supplementary Figure 3.** **Baseline correlation differences in microbial co-abundance networks across treatment groups.** Male C57BL6/J mice aged 8–10 weeks were treated with either PBS or 2.5 mmol·kg⁻¹·d⁻¹ ammonium acetate (AmAc) for 42 days (treatment period). Two weeks after the osmotic pumps were installed, mice were randomized to usual activity (UA) or voluntary wheel running (VWR) for 4 weeks (intervention period). Fecal samples collected at baseline were analyzed for diversity and taxonomic composition. Amplicon sequence variance (ASV)-level correlation patterns were compared pairwise between treatment groups at baseline samples to assess similarity in microbial co-abundance structure. Pairwise comparisons of ASV-ASV correlation networks were performed to assess differences in microbial co-occurrence structure between groups at baseline. **A.** AmAc UA vs. PBS UA. **B.** PBS VWR vs. PBS UA. **C.** AmAc VWR vs. PBS VWR. **D.** AmAc VWR vs. AmAc UA. Each panel shows a correlation network of ASV-ASV comparisons. Red indicates stronger correlations in the first group; blue indicates stronger correlations in the second group. ASVs are color-coded by phylum. **Statistical analyses:** ASV-ASV correlation matrices between groups at baseline were computed using **Pearson correlation coefficients**. n = 8 samples per group. **Abbreviations: (**c): class, (f): family, (g): genus, (o) order, C: ***Candidatus*, Corio:** *Coriobacteriales*, L: *Lachnospiraceae*, E: *Eubacterium*, R: *Ruminococcus*, ss: *sensu stricto*.

**Supplementary Figure 4.** **Correlation difference matrices showing changes in microbial co-abundance across timepoints within each treatment group.** Male C57BL6/J mice aged 8–10 weeks were treated with either PBS or 2.5 mmol·kg⁻¹·d⁻¹ ammonium acetate (AmAc) for 42 days (treatment period). Two weeks after the osmotic pumps were installed, mice were randomized to usual activity (UA) or voluntary wheel running (VWR) for 4 weeks (intervention period). Samples were collected at baseline, pre-intervention, and post-intervention and were analyzed for diversity and taxonomic composition. Amplicon sequence variance (ASV)-level correlation patterns were compared across time points within each treatment group to assess similarity in microbial co-abundance structure. Pairwise comparisons of ASV-ASV correlation networks were performed between baseline and pre-intervention and pre-intervention to post-intervention to assess differences in microbial co-occurrence structure between groups. ASV-ASV Pearson correlation matrices were compared between timepoints within each group to evaluate shifts in microbial interaction between timepoints. **A.** PBS UA: Baseline vs. Pre-intervention **B.** PBS UA: Pre- vs. Post-intervention **C.** PBS-VWR: Baseline vs. Pre-intervention; **D.** PBS-VWR: Pre- vs. Post-intervention; **E.** AmAc-UA: Baseline vs. Pre-intervention; **F.** AmAc-UA: Pre- vs. Post-intervention; **G.** AmAc VWR: Baseline vs. Pre **H.** AmAc VWR: Pre- vs. Post- intervention. Each panel displays a network in which ASV-ASV correlations were compared. Red values indicate stronger correlations at the **later** time point, while blue colors indicate stronger correlations at the **earlier** time point. ASVs are labeled and color-coded by phylum (Firmicutes, Bacteroidota, Verrucomicrobiota, Proteobacteria). **Statistical analyses:** ASV-ASV correlations were computed using **Pearson correlation coefficients**. **Pearson correlation** was used to assess the relationship between mean ASV abundances across groups**.** n = 8 samples per timepoint. **Abbreviations: (**c): class, (f): family, (g): genus, (o) order, C: ***Candidatus*, Corio:** *Coriobacteriales*, L: *Lachnospiraceae*, E: *Eubacterium*, R: *Ruminococcus*, ss: *sensu stricto*.

**Supplementary Figure 5.** **Pre-intervention comparisons of ASV-level mean abundance across treatment groups.** Male C57BL6/J mice aged 8–10 weeks were treated with either PBS or 2.5 mmol·kg⁻¹·d⁻¹ ammonium acetate (AmAc) for 42 days (treatment period). Two weeks after the osmotic pumps were installed, mice were randomized to usual activity (UA) or voluntary wheel running (VWR) for 4 weeks (intervention period). Fecal samples collected 2 weeks after the osmotic pump was installed (pre-intervention) were analyzed for diversity and taxonomic composition. Pairwise comparisons of pre-intervention mean ASV abundances were performed to assess similarity in microbial community structure prior to the start of intervention. **A.** AmAc UA vs. PBS UA **B.** PBS VWR vs. PBS UA **C.** AmAc VWR vs. PBS VWR **D.** AmAc VWR vs. AmAc UA. Each panel displays a scatterplot of mean ASV abundances (log-transformed where appropriate), with each point representing one ASV. Pearson correlation coefficients (r) and associated p-values are shown in each panel to quantify similarity in microbial composition between groups. Additionally, each panel displays a correlation difference matrix (Δr) in which ASV-ASV Pearson correlation values from the second group were subtracted from those in the first group. Red indicates stronger correlations in the first group; blue indicates stronger correlations in the second group. ASVs are color-coded by phylum. **Statistical analyses:** Pearson correlation was used to assess similarity in mean ASV abundance between groups at the pre-intervention timepoint and to construct ASV–ASV co-abundance networks. Scatterplots display the strength and direction of correlation for shared ASVs, along with the corresponding r and p-values. n = 8 samples per group. **Abbreviations: (**c): class, (f): family, (g): genus, (o) order, C: ***Candidatus*, Corio:** *Coriobacteriales*, L: *Lachnospiraceae*, E: *Eubacterium*, R: *Ruminococcus*, ss: *sensu stricto*.

**Supplementary Figure 6.** **Post-intervention comparisons of ASV-level mean abundance across treatment groups.** Male C57BL6/J mice aged 8–10 weeks were treated with either PBS or 2.5 mmol·kg⁻¹·d⁻¹ ammonium acetate (AmAc) for 42 days (treatment period). Two weeks after the osmotic pumps were installed, mice were randomized to usual activity (UA) or voluntary wheel running (VWR) for 4 weeks (intervention period). Fecal samples collected 2 weeks after the osmotic pump was installed (pre-intervention) and were analyzed for diversity and taxonomic composition. Pairwise comparisons of post-intervention mean ASV abundances were performed to assess similarity in microbial community post-intervention. **A.** AmAc UA vs. PBS UA. **B.** PBS VWR vs. PBS UA. **C.** AmAc VWR vs. PBS VWR. **D.** AmAc VWR vs. AmAc UA. Each panel displays a scatterplot of mean ASV abundances, with each point representing one ASV. Pearson correlation coefficients (r) and associated p-values are shown to quantify the degree of similarity in microbial composition between groups. Additionally, each panel displays a correlation difference matrix (Δr), in which ASV-ASV Pearson correlation values from the second group were subtracted from those in the first group. Red indicates stronger correlations in the first group; blue indicates stronger correlations in the second group. **Statistical analyses:** Pearson correlation was used to assess similarity in mean ASV abundance between groups at the post intervention timepoint and to construct ASV–ASV co-abundance networks. Scatterplots display the strength and direction of correlation for shared ASVs, along with the corresponding r and p-values. n = 8 samples per group. **Abbreviations: (**c): class, (f): family, (g): genus, (o) order, C: ***Candidatus*, Corio:** *Coriobacteriales*, L: *Lachnospiraceae*, E: *Eubacterium*, R: *Ruminococcus*, ss: *sensu stricto*.

**Supplementary Figure 7.** **Within-group comparisons of mean ASV abundance from baseline to post-intervention.** Male C57BL6/J mice aged 8–10 weeks were treated with either PBS or 2.5 mmol·kg⁻¹·d⁻¹ ammonium acetate (AmAc) for 42 days (treatment period). Two weeks after the osmotic pumps were installed, mice were randomized to usual activity (UA) or voluntary wheel running (VWR) for 4 weeks (intervention period). Fecal samples collected 2 weeks after the osmotic pump was installed (pre-intervention) and were analyzed for diversity and taxonomic composition. Pairwise comparisons of post-intervention mean ASV abundances were performed to assess similarity in microbial community post-intervention. **A.** PBS-UA. **B.** AmAc-UA. **C.** PBS-VWR. **D.** AmAc-VWR. Each point represents one ASV, with its mean abundance plotted at baseline (x-axis) versus post-intervention (y-axis). Pearson correlation coefficients (r) and associated p-values are shown in each panel to quantify the consistency of ASV-level abundance patterns over time. Additionally, each panel displays a correlation difference matrix (Δr), where the ASV-ASV Pearson correlation values at baseline were subtracted from those at the post-intervention time point. Red indicates stronger correlations post-intervention; blue indicates stronger correlations at baseline. ASVs are color-coded by phylum. **Statistical analyses: Pearson correlation** was used to assess the relationship between mean ASV abundances at baseline and post-intervention. p < 0.05; **p** < 0.01; ***p*** < 0.001. n = 8 samples per group per timepoint. **Abbreviations: (**c): class, (f): family, (g): genus, (o) order, C: ***Candidatus*, Corio:** *Coriobacteriales*, L: *Lachnospiraceae*, E: *Eubacterium*, R: *Ruminococcus*, ss: *sensu stricto*.

**Supplementary Figure 8.** Male C57BL6/J mice aged 8–10 weeks were treated with either PBS or 2.5 mmol·kg⁻¹·d⁻¹ ammonium acetate (AmAc) for 42 days (treatment period). Two weeks after the osmotic pumps were installed, mice were randomized to usual activity (UA) or voluntary wheel running (VWR) for 4 weeks (intervention period). Stool samples were collected at three timepoints: baseline (prior to treatment or intervention), pre-intervention (2 weeks post-treatment), and post-intervention (4 weeks post-intervention). Post-intervention samples were sequenced and analyzed, and the Genus-level Bacillota/Bacteroidetes ratios were calculated from log_10_-transformed relative abundance data for comparisons. Bar heights indicate mean ± SD**. Statistical analyses:** White’s non-parametric test with pooled variance and bootstrapped p-values * p<0.05; n=8 mice per group

**Supplementary Figure 9.** Functional pathway and KEGG ortholog (KO) changes in PBS UA mice from pre- to post-intervention using PICRUSt2 and Tax4Fun2. Male C57BL6/J mice aged 8–10 weeks were treated with PBS 42 days (treatment period). Two weeks after osmotic pump implantation, mice were randomized to usual activity (UA) or voluntary wheel running (VWR) for 4 weeks (intervention period). Samples collected at pre- and post-intervention timepoints from PBS UA mice. **A.** PICRUSt2 MetaCyc pathway analysis showing top pathways ranked by log2 fold change (post vs pre). Red indicates pathways higher in the pre-intervention state. The corresponding forest plots display effect sizes and confidence intervals for the top pathways. Volcano plots show global pathway changes with log2 fold change and –log10(p-value). Network plots represent sample-to-sample functional similarity based on Bray–Curtis distance, with nodes colored by timepoint (pre vs post). **B.** PICRUSt2 KO analysis showing top KOs ranked by log2 fold change (post vs pre). Red indicates KOs higher in the pre-intervention state. Forest plots display effect sizes and confidence intervals. Volcano plots show global KO-level changes, and network plots illustrate functional similarity between samples across timepoints. **C.** Tax4Fun2 pathway analysis comparing pre- and post-intervention functional predictions in PBS UA mice. Volcano plots show global pathway-level changes, with individual pathways plotted by log2 fold change and –log10(p-value). Bar plots display selected pathways, with blue representing pre-intervention and red representing post-intervention abundance. No significant differences were observed (all p > 0.05). **Statistical analyses:** Two-sample t-tests were used to assess differences between pre- and post-intervention groups for PICRUSt2-derived pathways and KOs. For Tax4Fun2, pathway differences were assessed using t-tests on log-transformed abundance values. Network analysis was performed using Bray–Curtis distance and k-nearest neighbor (kNN) graph construction. n = 8 samples per timepoint. All functional results represent predicted metabolic potential inferred from 16S rRNA gene sequencing data.

**Supplementary Figure 10.** Functional pathway and KEGG ortholog (KO) changes in AmAc UA mice from pre- to post-intervention using PICRUSt2 and Tax4Fun2. Male C57BL6/J mice aged 8–10 weeks were treated with 2.5 mmol·kg⁻¹·d⁻¹ ammonium acetate (AmAc) for 42 days (treatment period). Two weeks after osmotic pump implantation, mice were randomized to usual activity (UA) or voluntary wheel running (VWR) for 4 weeks (intervention period). Samples collected at pre- and post-intervention timepoints from AmAc UA mice were used for functional prediction and comparison. **A.** PICRUSt2 MetaCyc pathway analysis showing top pathways ranked by log2 fold change (post vs pre). Blue indicates pathways higher in the post-intervention state, while red indicates pathways higher in the pre-intervention state. Corresponding forest plots display effect sizes with confidence intervals for the top pathways. Volcano plots show global pathway-level changes with log2 fold change and –log10(p-value). Network plots represent sample-to-sample functional similarity based on Bray–Curtis distance, with nodes colored by timepoint (pre vs post). **B.** PICRUSt2 KO analysis showing top KOs ranked by log2 fold change (post vs pre). Red indicates KOs higher in the pre-intervention state. Forest plots display effect sizes and confidence intervals, while volcano plots show global KO-level changes. Network plots illustrate functional similarity between samples across timepoints. **C.** Tax4Fun2 pathway analysis comparing pre- and post-intervention functional predictions in AmAc UA mice. Volcano plots show global pathway-level changes, with individual pathways plotted by log2 fold change and –log10(p-value). Bar plots display pathway-level differences, with red indicating increased abundance in the post-intervention state and blue indicating decreased abundance. Box plots show selected pathways with significant differences between pre- and post-intervention samples. **Statistical analyses:** Two-sample t-tests were used to assess differences between pre- and post-intervention groups for PICRUSt2-derived pathways and KOs. For Tax4Fun2, pathway differences were assessed using t-tests on log-transformed abundance values. Network analysis was performed using Bray–Curtis distance and k-nearest neighbor (kNN) graph construction. p < 0.05 was considered statistically significant. n = 8 samples per timepoint. All functional results represent predicted metabolic potential inferred from 16S rRNA gene sequencing data.

**Supplementary Figure 11**. Functional pathway and KEGG ortholog (KO) changes in PBS VWR mice from pre- to post-intervention using PICRUSt2 and Tax4Fun2. Male C57BL6/J mice aged 8–10 weeks were treated with PBS for 42 days (treatment period). Two weeks after osmotic pump implantation, mice were randomized to usual activity (UA) or voluntary wheel running (VWR) for 4 weeks (intervention period). Samples collected at pre- and post-intervention timepoints from PBS VWR mice were used for functional prediction and comparison. **A.** PICRUSt2 MetaCyc pathway analysis showing top pathways ranked by log2 fold change (post vs pre). Blue indicates pathways higher in the post-intervention state, while red indicates pathways higher in the pre-intervention state. Corresponding forest plots display effect sizes with confidence intervals for the top pathways. Volcano plots show global pathway-level changes with log2 fold change and –log10(p-value). Network plots represent sample-to-sample functional similarity based on Bray–Curtis distance, with nodes colored by timepoint (pre vs post). **B**. PICRUSt2 KO analysis showing top KOs ranked by log2 fold change (post vs pre). Blue indicates KOs higher in the post-intervention state, while red indicates KOs higher in the pre-intervention state. Forest plots display effect sizes and confidence intervals, volcano plots show global KO-level changes, and network plots illustrate functional similarity between samples across timepoints. **C.** Tax4Fun2 pathway analysis comparing pre- and post-intervention functional predictions in PBS VWR mice. Volcano plots show global pathway-level changes, with individual pathways plotted by log2 fold change and –log10(p-value). Bar plots display pathway-level differences, with red indicating increased abundance in the post-intervention state and blue indicating decreased abundance. Box plots show selected pathways with significant differences between pre- and post-intervention samples. **Statistical analyses:** Two-sample t-tests were used to assess differences between pre- and post-intervention groups for PICRUSt2-derived pathways and KOs. For Tax4Fun2, pathway differences were assessed using t-tests on log-transformed abundance values. Network analysis was performed using Bray–Curtis distance and k-nearest neighbor (kNN) graph construction. p < 0.05 was considered statistically significant. n = 8 samples per timepoint. All functional results represent predicted metabolic potential inferred from 16S rRNA gene sequencing data.

**Supplementary Figure 12.** Functional pathway and KEGG ortholog (KO) changes in AmAc VWR mice from pre- to post-intervention using PICRUSt2 and Tax4Fun2. Male C57BL6/J mice aged 8–10 weeks were treated with 2.5 mmol·kg⁻¹·d⁻¹ ammonium acetate (AmAc) for 42 days (treatment period). Two weeks after osmotic pump implantation, mice were randomized to usual activity (UA) or voluntary wheel running (VWR) for 4 weeks (intervention period). Samples collected at pre- and post-intervention timepoints from AmAc VWR mice were used for functional prediction and comparison. **A.** PICRUSt2 MetaCyc pathway analysis showing top pathways ranked by log2 fold change (post vs pre). Blue indicates pathways higher in the post-intervention state, while red indicates pathways higher in the pre-intervention state. Corresponding forest plots display effect sizes with confidence intervals for the top pathways. Volcano plots show global pathway-level changes with log2 fold change and –log10(p-value). Network plots represent sample-to-sample functional similarity based on Bray–Curtis distance, with nodes colored by timepoint (pre vs post). **B.** PICRUSt2 KO analysis showing top KOs ranked by log2 fold change (post vs pre). Blue indicates KOs higher in the post-intervention state, while red indicates KOs higher in the pre-intervention state. Forest plots display effect sizes and confidence intervals, volcano plots show global KO-level changes, and network plots illustrate functional similarity between samples across timepoints. **C.** Tax4Fun2 pathway analysis comparing pre- and post-intervention functional predictions in AmAc VWR mice. Volcano plots show global pathway-level changes, with individual pathways plotted by log2 fold change and –log10(p-value). Bar plots display pathway-level differences, with red indicating increased abundance in the post-intervention state and blue indicating decreased abundance. Box plots show selected pathways with significant differences between pre- and post-intervention samples. **Statistical analyses:** Two-sample t-tests were used to assess differences between pre- and post-intervention groups for PICRUSt2-derived pathways and KOs. For Tax4Fun2, pathway differences were assessed using t-tests on log-transformed abundance values. Network analysis was performed using Bray–Curtis distance and k-nearest neighbor (kNN) graph construction. p < 0.05 was considered statistically significant. n = 8 samples per timepoint. All functional results represent predicted metabolic potential inferred from 16S rRNA gene sequencing data.

**Supplementary Figure 13.** Comparison of functional predictions between PICRUSt2 and Tax4Fun2 across experimental groups. Functional predictions were generated from 16S rRNA data using both PICRUSt2 and Tax4Fun2. Scatter plots compare KEGG ortholog (KO) abundance predicted by PICRUSt2 and Tax4Fun2 for each experimental group: PBS UA, PBS VWR, AmAc UA, and AmAc VWR. Each point represents an individual KO. Left panels show raw predicted KO abundance, and right panels show log10-transformed abundance values. Pearson correlation coefficients (r) are shown for each group, indicating the strength of agreement between methods. Across all groups, moderate correlations were observed between PICRUSt2 and Tax4Fun2 predictions, with the strongest agreement in AmAc UA (r = 0.573) and PBS VWR (r = 0.511), and lower agreement in PBS UA (r = 0.424) and AmAc VWR (r = 0.482). **Statistical analyses:** Pearson correlation was used to assess agreement between PICRUSt2 and Tax4Fun2 predicted KO abundances. Each point represents a shared KO between methods. n = number of KOs shared between PICRUSt2 and Tax4Fun2 for each group.

**Supplementary Figure 14.** Differential functional pathway and KEGG ortholog (KO) analysis across treatment and activity groups using PICRUSt2. Male C57BL6/J mice aged 8–10 weeks were treated with either PBS or 2.5 mmol·kg⁻¹·d⁻¹ ammonium acetate (AmAc) for 42 days (treatment period). Two weeks after osmotic pump implantation, mice were randomized to usual activity (UA) or voluntary wheel running (VWR) for 4 weeks (intervention period). Functional predictions were generated from 16S rRNA data using PICRUSt2 and analyzed across group comparisons. **A.** MetaCyc pathway analysis showing top pathways ranked by –log10(p-value) for each pairwise comparison: AmAc UA vs PBS UA, AmAc VWR vs PBS VWR, AmAc VWR vs AmAc UA, and PBS VWR vs PBS UA. Bar plots display pathways with the strongest statistical differences across comparisons. **B.** KO analysis showing top 15 KOs ranked by mean difference for each pairwise comparison. Blue indicates higher abundance in the first group, and red indicates higher abundance in the comparison group. Bar plots highlight the direction and magnitude of functional differences between groups. Summary of the number of significantly different KOs (uncorrected p < 0.05) identified for each comparison. **Statistical analyses:** Two-sample t-tests were used to assess differences in KO and pathway abundance between groups. Mean differences and p-values were calculated for each comparison, and features were ranked based on statistical significance. n = 8 samples per group.

**Supplementary Figure 15.** KEGG ortholog (KO) differential analysis, volcano plots, and network comparisons across treatment and activity groups using PICRUSt2. Male C57BL6/J mice aged 8–10 weeks were treated with either PBS or 2.5 mmol·kg⁻¹·d⁻¹ ammonium acetate (AmAc) for 42 days (treatment period). Two weeks after osmotic pump implantation, mice were randomized to usual activity (UA) or voluntary wheel running (VWR) for 4 weeks (intervention period). Functional predictions were generated from 16S rRNA data using PICRUSt2 and compared across group conditions. **A.** AmAc UA vs PBS UA comparison. Forest plots display KEGG ortholog (KO) odds ratios, with points representing log_10_ transformed odds ratios and horizontal lines indicating confidence intervals. Positive values indicate enrichment in AmAc UA, while negative values indicate enrichment in PBS UA. Volcano plots show global KO differences using mean differences and –log_10_(p-value). Network plots represent sample-to-sample functional similarity based on Bray–Curtis distance, with nodes colored by group. **B.** AmAc VWR vs PBS VWR comparison. Forest plots display KO odds ratios highlighting differences between treatment groups under exercise conditions. Volcano plots show KO-level differences across groups. Network plots illustrate functional similarity and clustering between AmAc VWR and PBS VWR samples. **C.** AmAc VWR vs AmAc UA comparison. Forest plots display KO odds ratios comparing exercise effects within the AmAc group. Volcano plots show KO-level changes associated with voluntary wheel running. Network plots demonstrate separation between pre- and post-intervention functional profiles within AmAc-treated mice. **D.** PBS VWR vs PBS UA comparison. Forest plots display KO odds ratios comparing exercise effects within the PBS group. Volcano plots show global KO-level changes between conditions. Network plots illustrate functional similarity between sedentary and exercised PBS mice. **Statistical analyses:** Logistic regression was used to estimate odds ratios and confidence intervals for KO associations between groups. Two-sample t-tests were used to calculate mean differences and p-values for volcano plots. Network analysis was performed using Bray–Curtis distance and k-nearest neighbor (kNN) graph construction. n = 8 samples per group.

**Supplementary Figure 16.** Tax4Fun2 KEGG ortholog (KO) differential analysis across treatment and activity groups. Male C57BL6/J mice aged 8–10 weeks were treated with either PBS or 2.5 mmol·kg⁻¹·d⁻¹ ammonium acetate (AmAc) for 42 days (treatment period). Two weeks after osmotic pump implantation, mice were randomized to usual activity (UA) or voluntary wheel running (VWR) for 4 weeks (intervention period). Functional predictions were generated from 16S rRNA data using Tax4Fun2 and compared across group conditions. **A.** AmAc UA vs PBS UA comparison. Forest plots display KO mean differences between groups, with horizontal lines representing variability across samples. Volcano plots show global KO-level differences based on mean difference and –log10(p-value). Box plots display selected KOs with group-level differences in abundance. **B.** AmAc VWR vs PBS VWR comparison. Forest plots show KO-level differences between treatment groups under exercise conditions. Volcano plots illustrate global KO-level changes, and box plots display selected KOs with significant differences between groups. **C.** AmAc VWR vs AmAc UA comparison. Forest plots display KO-level differences associated with voluntary wheel running within the AmAc group. Volcano plots show global KO changes, and box plots highlight selected KOs with significant differences between pre- and post-intervention conditions. **D.** PBS VWR vs PBS UA comparison. Forest plots display KO-level differences associated with exercise within the PBS group. Volcano plots show global KO-level changes, and box plots display selected KOs with significant differences between groups. **E.** Heatmap of top KOs across all groups. Hierarchical clustering of KEGG orthologs based on abundance across AmAc UA, AmAc VWR, PBS UA, and PBS VWR groups. Color intensity represents relative abundance of each KO, illustrating group-specific clustering patterns and functional differences. **Statistical analyses:** Two-sample t-tests were used to assess differences in KO abundance between groups. Mean differences and p-values were calculated for each comparison. Volcano plots display –log10(p-values) versus mean differences. Heatmap clustering was performed using hierarchical clustering based on Euclidean distance. n = 8 samples per group.

**Supplementary Figure 17.** Comparison of functional predictions between PICRUSt2 and Tax4Fun2 across treatment and activity groups. Functional predictions were generated from 16S rRNA data using PICRUSt2 and Tax4Fun2 and compared across group conditions. **A.** AmAc UA vs PBS UA comparison. Scatter plots show the relationship between KEGG ortholog (KO) abundances predicted by PICRUSt2 and Tax4Fun2. Each point represents a KO. Pearson correlation coefficients (r) are shown to quantify agreement between methods. Directional agreement plots show concordance in effect size direction between methods. **B.** AmAc VWR vs PBS VWR comparison. Scatter plots display the relationship between PICRUSt2 and Tax4Fun2 predicted KO abundances under exercise conditions. Directional agreement plots quantify concordance in effect size direction between methods. **C.** AmAc VWR vs AmAc UA comparison. Scatter plots show KO-level agreement between methods for within-treatment exercise comparisons. Directional agreement plots indicate the proportion of KOs with a consistent direction of change between methods. **D.** PBS VWR vs PBS UA comparison. Scatter plots display KO-level agreement between methods for within-control exercise comparisons. Directional agreement plots quantify consistency in the direction of change across methods. **E.** Summary of directional agreement across comparison groups. Bar plot shows the percentage of KOs with concordant direction of change between PICRUSt2 and Tax4Fun2 for each comparison, illustrating modest agreement across all conditions. These analyses show limited concordance between functional prediction methods. **Statistical analyses:** Pearson correlation was used to assess agreement between PICRUSt2 and Tax4Fun2 predicted KO abundances. Directional agreement was calculated as the percentage of KOs with concordant direction of change between methods. n = number of shared KOs across both prediction platforms for each comparison.

**Supplementary Figure 18.** Correlation analysis and functional taxa abundance across treatment and activity groups. Male C57BL6/J mice aged 8–10 weeks were treated with either PBS or 2.5 mmol·kg⁻¹·d⁻¹ ammonium acetate (AmAc) for 42 days (treatment period). Two weeks after osmotic pump implantation, mice were randomized to usual activity (UA) or voluntary wheel running (VWR) for 4 weeks (intervention period). Functional correlations and taxa associated with SCFA production and urease activity were analyzed across all groups. **A.** Correlation heatmaps showing relationships between physiological parameters, metabolic markers, and predicted functional features within each group (PBS UA, AmAc UA, PBS VWR, AmAc VWR). Correlation coefficients (r) are displayed, with color intensity representing strength and direction of association. **B.** Relative abundance of taxa grouped by functional classification, including SCFA-producing taxa, urease-associated taxa, and taxa with both functional capabilities. Individual taxa including Lachnospiraceae, Muribaculaceae, Eubacterium, Bacteroidales, Alistipes, and Clostridium sensu stricto are shown across experimental groups. Bar graphs display mean ± SD with individual data points. **Statistical analyses:** Pearson correlation coefficients were used to assess relationships between variables in correlation heatmaps. Group comparisons for taxa abundance were performed using one-way ANOVA followed by Fischer LSD post-hoc analysis. * p < 0.05; ** p < 0.01. n = 8 mice per group.
