# Supplementary material for: Voluntary wheel running modulates murine gut microbiome during hyperammonemic stress
Source: Microbiol Spectr. 2026 Jul 15;14(8):e03194-25. doi: 10.1128/spectrum.03194-25 (PMC13435939; doi:10.1128/spectrum.03194-25)
Supplement: Supplementary file 3 [file spectrum.03194-25-s0004.docx]

S Table 1 Correlations between microbial functional groups and host measures

| **Group** | **Microbial Feature** | **Host Parameter** | **Direction** | **r-value** | **P value** |
| --- | --- | --- | --- | --- | --- |
| **PBS-UA** | SCFA | Skeletal muscle ammonia | + | 0.78 | 0.02 |
|  | SCFA | Gastroc weight | + | 0.69 | 0.05 |
|  | Urease | Skeletal muscle ammonia | - | -0.59 | 0.12 |
|  | SCFA+Urease | Plasma ammonia | - | -0.54 | 0.17 |
| **AmAc-UA** | SCFA | Running distance | + | 0.71 | 0.04 |
|  | SCFA | eIF2α phosphorylation | + | 0.66 | 0.07 |
|  | SCFA | Plasma ammonia | + | 0.57 | 0.14 |
|  | Urease | Grip strength | - | -0.76 | 0.03 |
|  | SCFA+Urease | AST expression | + | 0.86 | 0.01 |
| **PBS-VWR** | SCFA | Gastroc weight | - | -0.64 | 0.08 |
|  | SCFA | eIF2α phosphorylation | - | -0.67 | 0.07 |
|  | Urease | Running distance | + | 0.68 | 0.06 |
|  | Urease | Puromycin incorporation | + | 0.88 | 0.04 |
|  | Urease | Gastroc weight | + | 0.77 | 0.02 |
|  | SCFA+Urease | Complex II expression | * | 0.85 | 0.01 |
|  |  |  |  |  |  |
| **AmAc-VWR** | SCFA | Grip strength | + | 0.55 | 0.27 |
|  | Urease | Running distance | + | 0.88 | 0.01 |
|  | Urease | Gastroc weight | + | 0.89 | 0.01 |
|  | Urease | Grip Strength | + | 0.55 | 0.04 |
|  | Urease | Complex I | + | 0.44 | 0.04 |
|  | Urease | Complex II | - | -0.79 | 0.03 |
|  | SCFA+Urease | Skeletal muscle ammonia | - | -0.65 | 0.29 |
|  | SCFA+Urease | ADP | + | 0.79 | 0.04 |
