# Supplementary material for: Voluntary wheel running modulates murine gut microbiome during hyperammonemic stress
Source: Microbiol Spectr. 2026 Jul 15;14(8):e03194-25. doi: 10.1128/spectrum.03194-25 (PMC13435939; doi:10.1128/spectrum.03194-25)
Supplement: Supplementary file 4 [file spectrum.03194-25-s0006.docx]

| **Feature / Domain** | **PICRUSt2** | **Tax4Fun2** | **Overlap** | **Complementarity** |
| --- | --- | --- | --- | --- |
| Core Algorithm | Phylogenetic placement + hidden-state prediction; copy-number correction | Taxonomic mapping (SILVA → KEGG genomes) | Both infer gene content from taxonomy | Phylogeny captures novel taxa; mapping confirms known taxa |
| Prediction Confidence | NSTI score (distance to reference genomes) | Mapping efficiency / fraction of mapped reads | Both depend on reference genome proximity | Orthogonal quality control metrics improve interpretation |
| Functional Resolution | Gene families → pathway inference (multi-level) | Primarily KEGG pathway aggregation | Both generate KO-based profiles | Concordance across results strengthen confidence in predicted functional changes |
| Sensitivity vs Specificity | Higher sensitivity; detects more differences | More conservative; fewer significant pathways | Both identify core pathways | PICRUSt2 provides greater sensitivity; Tax4Fun2 higher specificity |
| Functional Outputs | KEGG Orthologs, EC numbers, pathways | KEGG Orthologs and pathways | Comparable functional space | Cross-validation increases confidence |
| Performance with Novel Taxa | Stronger via phylogenetic inference | Weaker if no close genome match | Both limited by databases | PICRUSt2 fills gaps when mapping fails, infers function for unmatched taxa. Tax4Fun2 relies on direct mapping but can use custom databases. |
| Functional Redundancy | Not primary feature | Explicit redundancy metrics | Community-level inference | Tax4Fun2 adds ecological interpretation |
| Interpretation of Concordance | Concordant results indicate robust functional signals | Discordance = hypothesis-generating | Both detect overlapping biology | Guides validation strategy |
| Major Limitation | Predicts functional potential, not activity | Predicts functional potential, not activity | Inference-based methods | Requires metagenomics/metabolomics validation |

**S. Table 3. Comparison of PICRUSt2 and Tax4Fun2 for Prediction of Microbial Functional Potential from 16S rRNA Data**

EC: Enzyme Commission; KEGG: Kyoto Encyclopedia of Genes and Genomes; KO: KEGG Ortholog; NSTI: Nearest Sequenced Taxon Index; PICRUSt2: Phylogenetic Investigation of Communities by Reconstruction of Unobserved States 2; QC: Quality Control; SILVA: Ribosomal RNA Gene Database Project; Tax4Fun2: Taxonomy-based Functional Profiling 2
